# Caspase-8 auto-cleavage regulates programmed cell death and collaborates with RIPK3/MLKL to prevent lymphopenia

**DOI:** 10.1038/s41418-022-00938-9

**Published:** 2022-01-21

**Authors:** Xiaoming Li, Fang Li, Xixi Zhang, Haiwei Zhang, Qun Zhao, Ming Li, Xiaoxia Wu, Lingxia Wang, Jianling Liu, Xuanhui Wu, Yangjing Ou, Mingyan Xing, Yue Zhang, Jiangshan Deng, Xiuzhe Wang, Yan Luo, Jinbao Li, Yuwu Zhao, Haibing Zhang

**Affiliations:** 1 CAS Key Laboratory of Nutrition, Metabolism and Food Safety, Shanghai Institute of Nutrition and Health, University of Chinese Academy of Sciences, Chinese Academy of Sciences, Shanghai, China; 2 Department of Anesthesiology, Shanghai General Hospital, Shanghai Jiao tong University School of Medicine, Shanghai, China; 3 The Second Affiliated Hospital, School of Medicine, Southern University of Science and Technology, Shenzhen, China; 4 Department of Anesthesiology, Ruijin Hospital, Shanghai Jiao Tong University School of Medicine, Shanghai, China; 5 Department of Neurology, Shanghai Jiao Tong University Affiliated Sixth People’s Hospital, Shanghai, China

**Keywords:** Cell death and immune response, Immune cell death

## Abstract

Caspase-8 is an initiator of death receptor-induced apoptosis and an inhibitor of RIPK3-MLKL-dependent necroptosis. In addition, caspase-8 has been implicated in diseases such as lymphoproliferation, immunodeficiency, and autoimmunity in humans. Although auto-cleavage is indispensable for caspase-8 activation, its physiological functions remain poorly understood. Here, we generated a caspase-8 mutant lacking E385 in auto-cleavage site knock-in mouse (*Casp8*^*ΔE385/ΔE385*^). *Casp8*^*ΔE385/ΔE385*^ cells were expectedly resistant to Fas-induced apoptosis, however, *Casp8*^Δ*E385/ΔE385*^ cells could switch TNF-α-induced apoptosis to necroptosis by attenuating RIPK1 cleavage. More importantly, CASP8(ΔE385) sensitized cells to RIPK3-MLKL-dependent necroptosis through promoting complex II formation and RIPK1-RIPK3 activation. Notably, *Casp8*^Δ*E385/ΔE385*^*Ripk3*^*−/−*^ mice partially rescued the perinatal death of *Ripk1*^*−/−*^ mice by blocking apoptosis and necroptosis. In contrast to the *Casp8*^*−/−*^*Ripk3*^*−/−*^ and *Casp8*^*−/−*^*Mlkl*^*−/−*^ mice appearing autoimmune lymphoproliferative syndrome (ALPS), both *Casp8*^Δ*E385/ΔE385*^*Ripk3*^*−/−*^ and *Casp8*^Δ*E385/ΔE385*^*Mlkl*^*−/−*^ mice developed transplantable lymphopenia that could be significantly reversed by RIPK1 heterozygosity, but not by RIPK1 kinase dead mutation. Collectively, these results demonstrate previously unappreciated roles for caspase-8 auto-cleavage in regulating necroptosis and maintaining lymphocytes homeostasis.

## Introduction

Caspase-8 is a cysteinyl aspartate-specific protease that critically mediates extrinsic apoptosis [1–5] but also inhibits necroptosis [6–11]. In addition, caspase-8 is known to be crucially involved in the inflammatory response by acting as a scaffolding protein [12–16]. Previous studies demonstrated the death of *Casp8*^*−/−*^ mice from RIPK3-MLKL mediated necroptosis [9, 10]. This result highlights the critical role of the catalytic activity of caspase-8-cFLIP complex in necroptosis inhibition [9]. In accordance with that, the conditional ablation of caspase-8 in the intestinal epithelial cells or keratinocytes also leads to the inflammation and aberrant cell death in the intestine and skin, respectively [17–21]. This can be prevented by the co-deletion of *Ripk3* [17–21], indicating that caspase-8 is required for tissue homeostasis by suppressing necroptosis. Moreover, mice expressing catalytically inactive RIPK3 D161N exhibit caspase-8-dependent embryonic lethality [22], suggesting that caspase-8 mediated apoptosis plays an essential role during embryonic development. In addition, caspase-8 phosphorylation mimic T265E knock-in mice were lately reported to be embryonically lethal [23], which indicated the phosphorylation of caspase-8 impaired the blockade of necroptosis during embryo development. Furthermore, caspase-8-mediated apoptosis in association with caspase-11 and gasdermin-D-mediated epithelial cell death to regulate gut homeostasis and inflammation [20, 24]. Recent studies have demonstrated that deficiency of the enzymatic activity of CASP8(C362S)/CASP8(C362A) not only promotes necroptosis but also triggers pyroptosis when necroptosis is inhibited in vivo [6, 8]. The expression of catalytically inactive caspase-8 leads to embryonic lethality in mice that can be prevented by deletion of *Ripk3* or co-ablation of *Mlkl* and *Casp1* [6, 8], suggesting that the enzymatic activity of caspase-8 plays a critical role in the regulation of pyroptosis when apoptosis and necroptosis are compromised. In addition to the regulation of cell death, caspase-8 contributes to the maintenance of immune homeostasis [11, 25–27]. When embryonic lethality in *Casp8*-deficient mice is rescued by *Ripk3* or *Mlkl* ablation, the *Casp8*^*−/−*^*Ripk3*^*−/−*^ and *Casp8*^*−/−*^*Mlkl*^*−/−*^ mice develop lymphadenopathy [11] that resembles the abnormality observed in Fas ligand (FasL, CD95L) [28] or FAS [29, 30] deficient mice and human autoimmune lymphoproliferative syndrome (ALPS) [31, 32]. *Casp8*^*C362A/C362A*^*Ripk3*^*−/−*^ mice also develop splenomegaly [8], indicating the potential of catalytic activity of caspase-8 in immune homeostasis. Besides, caspase-8 mutation in humans causes immunodeficiency [26] in addition to ALPS, which can be explained by the mechanisms that caspase-8 cleaves and inactivates a cytokine production suppressor NEDD4-binding protein 1 (N4BP1) [33]. However, caspase-8 mutations in humans have also been linked to inflammatory bowel disease (IBD) [34] and multi-organ lymphocytic infiltration with granulomas [27], and the precise mechanisms underlying this relationship remain elusive.

On ligating with the death receptor, auto-cleavage leads to the activation of caspase-8 [35–40]. This initiates apoptosis, and in turn, inhibits necroptosis by cleaving critical necroptotic mediators such as CYLD [41], c-FLIP [42], RIPK1 [7, 43] and RIPK3 [44]. Furthermore, complete caspase-8 activation requires dimerization and auto-cleavage of procaspase-8 to unlock the enzymatic activity [35–37, 45]. The mice harboring mutation of caspase-8 auto-cleavage site at D387 developed normally and was impaired in extrinsic apoptosis in vivo [7, 13, 46, 47], and recent study showed that the non-cleavable caspase-8 caused inflammation and induced ASC oligomerization in the lack of FADD [13]. However, the role of auto-cleavage of caspase-8 in regulating necroptosis and cell death-independent function remains undefined.

Here, we generated knock-in mouse bearing caspase-8 mutation lacking E385 in the auto-cleavage site (*Casp8*^Δ*E385/ΔE385*^) and found that caspase-8 mutation CASP8(ΔE385) not only switches TNF-α induced apoptosis to necroptosis by suppressing RIPK1 cleavage, but also unexpectedly promoted necroptosis through promoting complex II and RIPK1-RIPK3 activation. In addition, *Casp8*^Δ*E385/ΔE385*^*Ripk3*^*−/−*^ and *Casp8*^Δ*E385/ΔE385*^*Mlkl*^*−/−*^ mice developed lymphopenia with severe splenomegaly instead of the lymphoproliferative disease as observed in *Casp8*^*−/−*^*Ripk3*^*−/−*^ and *Casp8*^*−/−*^*Mlkl*^*−/−*^ mice. Collectively, these results suggest that caspase-8 auto-cleavage is not only required to mediate apoptosis but also inhibit necroptosis by negatively regulating complex II formation and stabilization and cooperates with RIPK3/MLKL maintaining lymphocytes homeostasis.

## Results

### *Casp8*^Δ*E385/ΔE385*^ mice are viable but develop a slight CD8^+^ T cell lymphopenia in the spleen

**Fig. 1:**
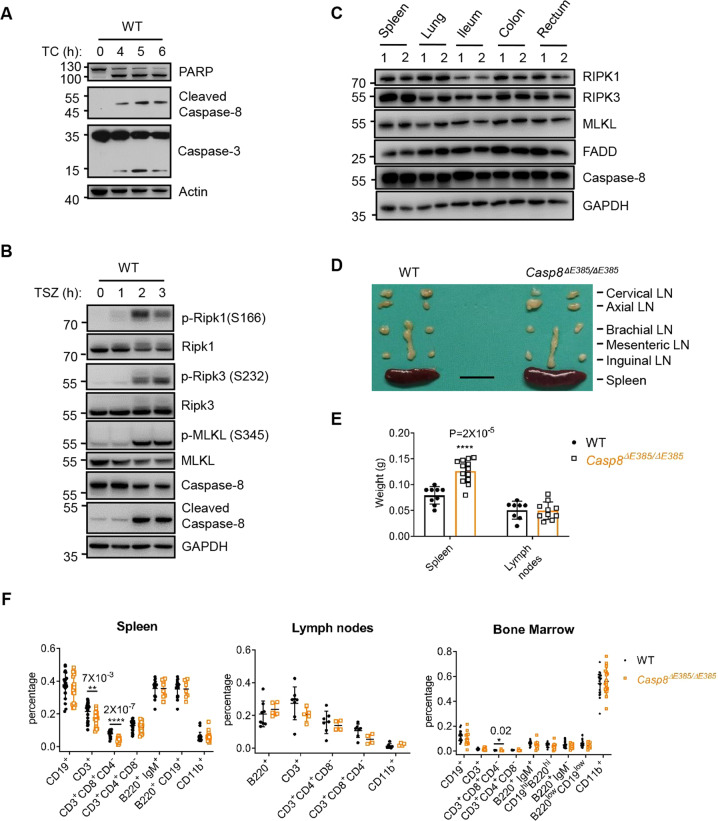
*Casp8*^*ΔE385/ΔE385*^ mice are viable and develop a slight lymphopenia. **A** Western blot of primary wild-type (WT) MDFs which were treated with TNF-α (40 ng/ml) +Cycloheximide (CHX) (40 μg/ml) (TC) for the indicated time. **B** Western blot of primary WT MDFs which were treated with TNF-α (20 ng/ml) +Smac mimetic (Smac) (1 μM) +zVAD (20 μM) (TSZ). **C** Western blot of RIPK1, RIPK3, MLKL, FADD, caspase-8, and GAPDH in the indicated organs of WT (1) and *Casp8*^*ΔE385/ΔE385*^ (2) mice. **D** Lymph nodes and spleens removed from 16-week old mice of indicated genotypes (scale bar, 1 cm). **E** Dot plot of weight of lymph nodes (parts showed in Fig. 1D) and spleens of 12- to 16-week old WT, *Casp8*^*ΔE385/ΔE385*^ mice. Bars, mean ± SD. *P* values above the asterisk (unpaired, two-tailed *t* test) ****p < 0.0001, compared to the WT mice. **F** Different cell subsets from spleen, lymph nodes (parts showed in Fig. 1D) and bone marrow of 12- to 16-week old WT and *Casp8*^*ΔE385/ΔE385*^ mice were analyzed by flow cytometry using the following markers: B cells (B220^+^ or CD19^+^), T cells (CD3^+^), CD4^+^ T cells (CD3^+^CD4^+^CD8^−^), CD8^+^ T cells (CD3^+^CD8^+^CD4^−^), Granulocytes and Macrophages (CD11b^+^), mature B cells in spleen (B220^+^IgM^+^ or B220^+^CD19^+^), immature and mature B cells in bone marrow (B220^+^ IgM^+^ or B220^hi^ CD19^hi^), progenitor B cells (pro-B) and precursor B cells (pre-B) in bone marrow (B220^+^ IgM^−^ or B220^low^ CD19^low^). Bars, mean ± SD. *P* values above the asterisk (unpaired, two-tailed *t* test) *p < 0.05, **p < 0.05, ****p < 0.0001.

Previous studies have demonstrated that auto-cleavage of caspase-8 is required for mediating apoptosis but not for inhibiting necroptosis during development because the mice expressing none-cleavable Caspase-8 are viable [7, 13, 46]. As expected, we observed that caspase-8 cleavage was gradually enhanced when apoptosis was induced by tumor necrosis factor α (TNF-α) plus cycloheximide (CHX) in wild-type mouse dermal fibroblasts (MDFs) (Fig. 1A). Notably, caspase-8 cleavage was also increased in response to necroptotic stimulation with TNF-α plus Smac mimetics (Smac) and the pan-caspase inhibitor Z-VAD-FMK (zVAD). This finding was verified by observing the increased levels of phosphorylated RIPK1, RIPK3, and MLKL necroptotic markers (Fig. 1B). Therefore, in addition to its role in mediating apoptosis, caspase-8 cleavage is also hypothesized to regulate necroptosis.

Previous studies established transgenic mice expressing caspase-8 D387A [7, 13, 46], which cannot be cleaved between the large and small catalytic subunits. Caspase-8 has a substrate preference for the tetrapeptide (Leu/Val)-Glu-X-Asp [48], which corresponds closely to the caspase-8 auto-processing substrate sequence, L384/E385/V386/D387. We therefore hypothesized that E385 of caspase-8 would also contribute to its auto-cleavage. To explore the contribution of caspase-8 (E385) in its auto-processing in vitro and in vivo, we generated a knock-in mouse that expressed caspase-8 lacking E385 in the auto-cleavage site between the large and small catalytic subunits (Fig. S1A). In contrast to the embryonic lethality observed in caspase-8 deficiency [49] and catalytically inactive caspase-8 mice [6, 8], *Casp8*^Δ*E385/ΔE385*^ mice were viable and matured normally (Fig. S1B), which was consistent with previously reported mouse lines expressing caspase-8(D387A) [7, 13, 46]. To test whether CASP8(ΔE385) is indeed unable to auto-process between the large and small catalytic subunits, we treated primary WT and *Casp8*^Δ*E385/ΔE385*^ BMDMs with LPS/BV6 to induce apoptosis. Compared with the dramatic caspase-8 cleavage in wild-type BMDMs, caspase-8 cleavage between the large and small catalytic subunits was confirmed to be blocked in *Casp8*^Δ*E385/ΔE385*^ BMDMs utilizing two different antibodies (Fig. S1C). Besides, it was observed that the expression of CASP8(ΔE385) in multiple tissues including spleen, lung, liver, kidney, colon, heart, ileum, and rectum was normal in *Casp8*^Δ*E385/ΔE385*^ mice (Fig. 1C and S1D), suggesting that the cleavage of caspase-8 is dispensable for its expression and stability in vivo. Next, we examined the effect of CASP8(ΔE385) on the pathologies. Histopathological examination demonstrated that the appearance of multiple tissues was indistinguishable in *Casp8*^Δ*E385/ΔE385*^ mice in comparison with the tissue appearance in WT mice (Fig. S1E). However, we observed that the *Casp8*^Δ*E385/ΔE385*^ mice developed slight splenomegaly with a mild decrease in the percentage of the CD8^+^ T cells in the spleen and bone marrow (Fig. 1D–F). However, no differences were observed between *Casp8*^Δ*E385/ΔE385*^ and WT mice with respect to the B cells and the myeloid cell subsets obtained from the spleen, lymph nodes, and bone marrow (Fig. 1F). These results show that the *Casp8*^Δ*E385/ΔE385*^ mice are viable but develop a slight CD8^+^ T cell lymphopenia with splenomegaly.

### Apoptosis induced by TNF-α was switched to necroptosis by attenuating RIPK1 cleavage in *Casp8*^Δ*E385/ΔE385*^ cells

**Fig. 2:**
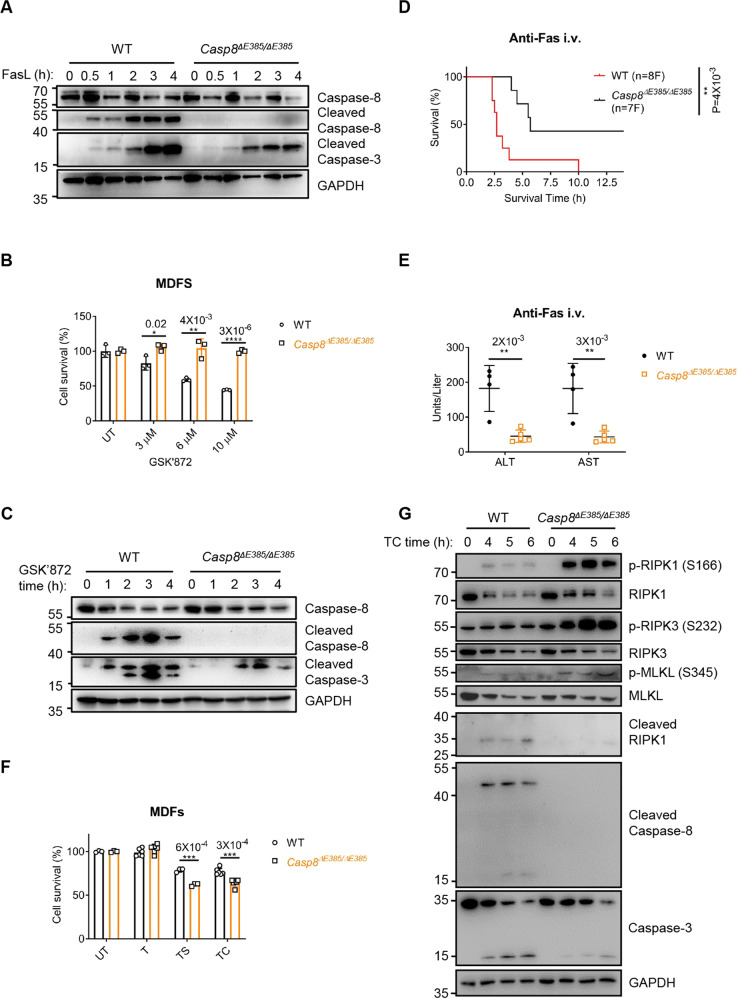
The CASP8(ΔE385) compromises apoptosis, particularly switches TNF-α induced apoptosis to necroptosis. **A** Western blotting analysis of the indicated protein in primary WT and *Casp8*^*ΔE385/ΔE385*^ thymocytes which were treated with FasL (Jo-2) (1 μg/ml) for the indicated time. The data are representative of three independent experiments. **B** Primary WT and *Casp8*^*ΔE385/ΔE385*^ MDFs were treated with GSK’872 in different concentration for the indicated time respectively. Bars, mean ± SD. *P* values above the asterisk (unpaired, two-tailed *t* test) *p < 0.05, **p < 0.01, ****p < 0.0001. **C** Western blotting analysis of protein expression of caspase-8, cleaved caspase-8, cleaved caspase-3 and GAPDH in primary WT and *Casp8*^*ΔE385/ΔE385*^ MDFs which were treated with GSK’872 (20 μM) for the indicated time. **D** Mouse survival curve of 8- to 12-week old mice after challenged by anti-Fas antibody (Jo-2, 0.5 μg/g, i.v.). F, female. *P* values alongside the asterisk, two-sided Log-rank (Mantel-Cox) test, **p < 0.01. **E** The alanine transaminase (ALT) and aspartate transaminase (AST) levels in serum of the 16-week old WT, *Casp8*^*ΔE385/ΔE385*^ mice 2.5 h after anti-Fas injection. Bars, mean ± SD. *P* values above the asterisk (unpaired, two-tailed *t* test), **p < 0.01. **F** Primary WT and *Casp8*^*ΔE385/ΔE385*^ MDFs were treated with TNF-α (20 ng/ml), TNF-α + Smac (1 μM) (TS), TNF-α + CHX (20 μg/ml) (TC) for 5 h. Bars, mean ± SD. *P* values above the asterisk (unpaired, two-tailed *t* test) ***p < 0.001. **G** Immunoblotting of the indicated protein expression in primary WT and *Casp8*^*ΔE385/ΔE385*^ MDFs which were treated with TNF-α (40 ng/ml) +CHX (40 μg/ml) (TC) for the indicated time.

Previous studies have demonstrated that the auto-cleavage of caspase-8 is essential for the apoptosis induced by the anti-Fas antibody Jo2, in vitro [7, 13, 46] and in vivo [13, 46]. Consistently, we observed that the thymocyte apoptosis induced by anti-Fas from *Casp8*^Δ*E385/ΔE385*^ mice was compromised compared to that from WT mice (Fig. S2A), and anti-Fas antibody also induced less caspase-3 cleavage in *Casp8*^Δ*E385/ΔE385*^ thymocytes (Fig. 2A). To further investigate the role of caspase-8 cleavage in apoptosis, we treated *Casp8*^Δ*E385/ΔE385*^ MDFs with a RIPK3 kinase inhibitor, GSK’872, to induce apoptosis [50]. We observed that *Casp8*^Δ*E385/ΔE385*^ MDFs were strongly resistant to apoptosis induced by GSK’872 (Fig. 2B). This finding was confirmed by attenuating the cleavage of caspase-3 in *Casp8*^Δ*E385/ΔE385*^ MDFs (Fig. 2C). To further verify the contribution of caspase-8 cleavage in apoptosis in vivo, we challenged the anti-Fas antibody, Jo2, by intravenous injection in *Casp8*^Δ*E385/ΔE385*^ and WT mice. In accordance with previous studies [13, 46], *Casp8*^Δ*E385/ΔE385*^ mice were significantly protected from the Jo2-induced lethal effects compared to WT mice (Fig. 2D). Accordingly, *Casp8*^Δ*E385/ΔE385*^ mice exhibited alleviated liver damage and decreased alanine aminotransferase (ALT)/aspartate aminotransferase (AST) concentrations in the plasma compared to the liver function in WT control mice (Fig. 2E and S2B). In line with these data, we observed the absence of caspase-8 cleavage and a significant decrease in caspase-3 cleavage in the livers of *Casp8*^Δ*E385/ΔE385*^ mice (Fig. S2C), suggesting that the lethal effects exerted by the anti-Fas antibody Jo2-induced apoptosis were decreased in *Casp8*^Δ*E385/ΔE385*^ mice in vivo. These results suggested that blocking cleavage between the large and small catalytic subunits by CASP8(ΔE385) is enough to prevent apoptosis in vitro and in vivo.

To further investigate whether CASP8(ΔE385) is required for TNF-α-induced apoptosis, we treated the *Casp8*^Δ*E385/ΔE385*^ MDFs with TNF-α plus Smac. In contrast to the WT MDFs showing increased caspase-8 cleavage, *Casp8*^Δ*E385/ΔE385*^ MDFs showed no detectable caspase-8 auto-cleavage between the large and small catalytic subunits (Fig. S3A). However, in contrast with the previous findings that the apoptosis induced by GSK’872 decreased in *Casp8*^Δ*E385/ΔE385*^ MDFs, we observed that increased cell death in *Casp8*^Δ*E385/ΔE385*^ MDFs upon stimulation with TNF-α plus Smac/CHX compared to the death in WT MDFs (Fig. 2F). Interestingly, we further observed that caspase-3 cleavage induced by TNF-α plus Smac in WT MDFs was decreased in *Casp8*^Δ*E385/ΔE385*^ MDFs (Fig. S3A). Given that the necroptosis suppression function of caspase-8 [51], we speculated that CASP8(ΔE385) could switch apoptosis to necroptosis under certain conditions. Therefore, we measured the markers of cell death pathways in MDFs in response to the stimulation by TNF-α/CHX and TNF-α/Smac. The *Casp8*^Δ*E385/ΔE385*^ MDFs showed upregulation in RIPK1, RIPK3, and MLKL phosphorylation but a decrease in RIPK1 and caspase-3 cleavage (Fig. 2G and S3A). This indicates that the CASP8(ΔE385) switched TNF-α/CHX and TNF-α/Smac induced caspase-3-dependent apoptosis to RIPK1-RIPK3-MLKL-mediated necroptosis owing to the attenuation of RIPK1 cleavage. Collectively, these results demonstrate that caspase-8 cleavage between the large and small catalytic subunits is required for mediating apoptosis, but CASP8(ΔE385) promotes cell death switch from apoptosis to RIPK1-RIPK3-MLKL-dependent necroptosis under certain conditions.

### CASP8(ΔE385) promotes necroptosis upon various necroptotic stimuli both in vitro and in vivo

**Fig. 3:**
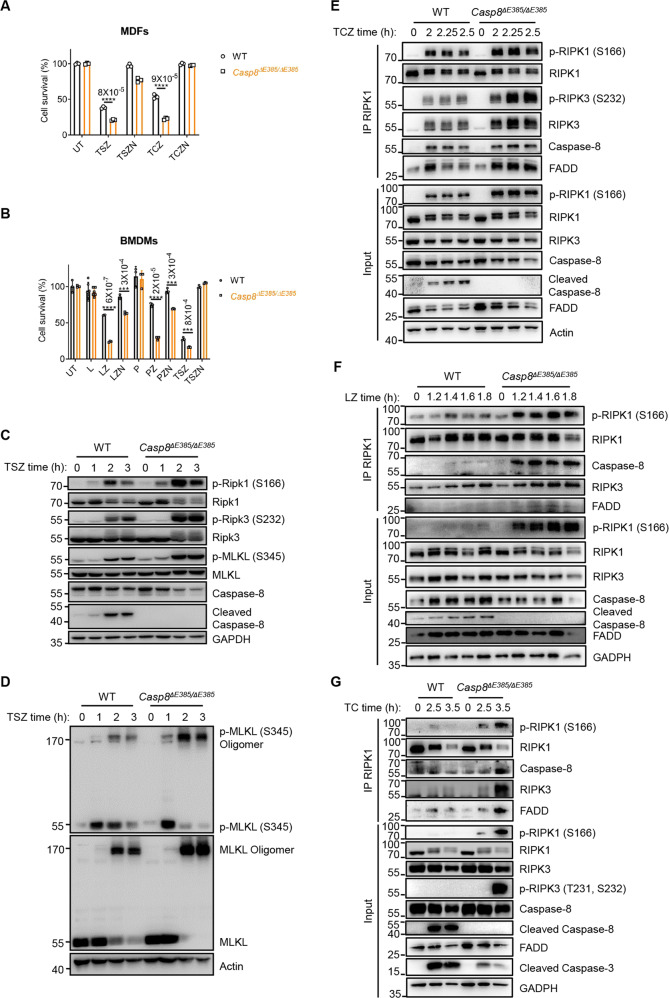
The CASP8(ΔE385) promotes necroptosis in vitro. **A** Primary WT and *Casp8*^*ΔE385/ΔE385*^ MDFs were treated with TNF-α (20 ng/ml)+Smac (1 μM)+zVAD (20 μM) (TSZ) and TNF-α + Smac + zVAD + Nec-1 (30 μM) (TSZN) for 6.45 h, TNF-α + CHX (20 μg/ml)+zVAD (20 μM) (TCZ) and TNF-α + CHX + zVAD + Nec-1 (30 μM) (TCZN) for 4.45 h. Bars, mean ± SD. *P* values above the asterisk (unpaired, two-tailed *t* test) ****p < 0.0001. **B** Primary WT and *Casp8*^*ΔE385/ΔE385*^ bone marrow derived macrophages (BMDMs) were treated with LPS (100 ng/ml), LPS + zVAD (20 μM) (LZ), LPS + zVAD + Nec-1 (30 μM) (LZN), poly(I:C) (100 μg/ml), poly(I:C) + zVAD (20 μM) (PZ), poly(I:C) + zVAD + Nec-1 (30 μM) (PZN), TNF-α + Smac + zVAD (TSZ), TNF-α + Smac + zVAD + Nec-1 (TSZN) for 3 h. Bars, mean ± SD. *P* values above the asterisk (unpaired, two-tailed *t* test) ***p < 0.001, ****p < 0.0001. **C** Immunoblotting of the indicated protein expression in primary WT and *Casp8*^*ΔE385/ΔE385*^ MDFs which were treated with TNF-α (20 ng/ml) +Smac (1 μM) +zVAD (20 μM) (TSZ) for the indicated time. **D** Immunoblotting of primary WT and *Casp8*^*ΔE385/ΔE385*^ MDFs which were treated with TNF-α (20 ng/ml) +Smac (1 μM) +zVAD (20 μM) (TSZ) for the indicated time. **E** WT and *Casp8*^*ΔE385/ΔE385*^ MDFs were treated with TNF-α (40 ng/ml)+CHX (40 μg/ml)+zVAD (50 μM) for the indicated time, complex II was immunoprecipitated using anti-RIPK1, the recruitment of RIPK3, FADD and caspase-8 were detected by western blotting. **F** Primary WT and *Casp8*^*ΔE385/ΔE385*^ BMDMs were treated with LPS (200 ng/ml)+zVAD (40 μM) followed by western blot and immunoprecipitation. **G** Primary WT and *Casp8*^*ΔE385/ΔE385*^ MDFs were treated with TNF-α (40 ng/ml)+CHX (40 μg/ml) followed by western blot and immunoprecipitation.

Caspase-8 suppresses RIPK3-MLKL mediated necroptosis [9–11], and caspase-8 catalytic activity is essential for inhibiting necroptosis during development, as demonstrated recently [6, 8]. To investigate the role of caspase-8 auto-cleavage in necroptosis regulation, we induced necroptosis in MDFs via TNF-α plus Smac and zVAD and in bone marrow-derived macrophages (BMDMs) via stimulation with LPS or poly(I:C) plus zVAD. Notably, we observed that *Casp8*^Δ*E385/ΔE385*^ MDFs and BMDMs showed excessive cell death compared to their WT counterparts, which could also be rescued by Nec-1 (Figs. 3A, B). RIPK1 [7], RIPK3 [52–54] and MLKL [55, 56] are the main executors of programmed necroptosis via cascade phosphorylation. To further investigate the mechanism by which caspase-8 cleavage regulates necroptosis, we first examined RIPK1-RIPK3-MLKL axis signaling. Indeed, compared with the WT MDFs, the *Casp8*^Δ*E385/ΔE385*^ MDFs showed significant increase in the phosphorylation of RIPK1, RIPK3, and MLKL and oligomerization of MLKL after TNF-α plus Smac/CHX and zVAD stimulation (Figs. 3C, D, and S3B). Similar results were observed in *Casp8*^Δ*E385/ΔE385*^ BMDMs in LPS plus zVAD-induced necroptosis (Fig. S3C).

As the pan-caspase inhibitor Z-VAD-FMK blocked the caspase-8 enzymatic activity both in wild-type and *Casp8*^Δ*E385/ΔE385*^ cells, we wondered why *Casp8*^Δ*E385/ΔE385*^ cells still showed excessive necroptosis compared with WT cells in the presence of zVAD. Previous study demonstrated that TNF-α induced cell death depends on complex II, which contains RIPK1, FADD, caspase-8, RIPK3 and MLKL [52–54, 57–61]. Regulated by several pro- and anti-apoptotic and pro- and anti-necroptotic proteins [6–8, 41, 43, 44, 62–64], complex-II can trigger apoptosis or necroptosis. Thus, we analyzed whether complex II was enhanced in *Casp8*^Δ*E385/ΔE385*^ MDFs under necroptotic stimulation. When MDFs were stimulated by TNF-α/CHX/zVAD, we found obviously sustained and enhanced interaction of RIPK3 and FADD with RIPK1 in *Casp8*^Δ*E385/ΔE385*^ MDFs than in wild-type cells (Fig. 3E). Furthermore, we detected dramatically increased p-Ser166 RIPK1 and p-Ser232 RIPK3 within complex II in TNFα/CHX/zVAD treated *Casp8*^Δ*E385/ΔE385*^ MDFs compared to WT MDFs (Fig. 3E). Besides, we also found enhanced complex II assembly in *Casp8*^Δ*E385/ΔE385*^ BMDMs under LPS/zVAD treatment (Fig. 3F).

**Fig. 4:**
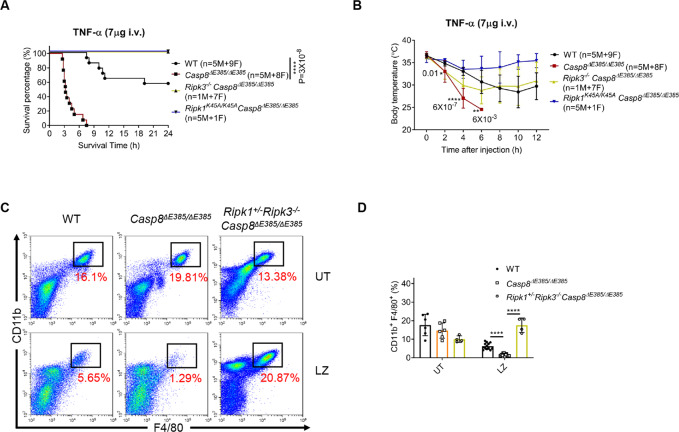
The CASP8(ΔE385) promotes necroptosis in vivo. **A** Mouse survival curve of 8- to 16-week old mice after injection by TNF-α (7 μg each mouse, i.v.). M, male, F, female. *P* values alongside the asterisk, by two-sided Log-rank (Mantel-Cox) test. ****p < 0.0001. **B** Body temperature of 8- to 16-week old mice after injection by TNF-α (7 μg each mouse, i.v.). M, male, F, female. Bars, mean ± SD. The significance of body temperature between WT and *Casp8*^*ΔE385/ΔE385*^ mice in the indicated time was described by *P* values below the asterisk (unpaired, two-tailed *t* test) *p < 0.05, **p < 0.01, ****p < 0.0001. **C** Representative peritoneal macrophages flow cytometric dot plots along CD11b versus F4/80 parameters. Untreated (UT), LPS + zVAD (LZ). **D** Dot plots of CD11b^+^ F4/80^+^ peritoneal macrophages of 8- to 12-week old WT, *Casp8*^*ΔE385/ΔE385*^ and *Ripk1*^*+/−*^*Ripk3*^*−/−*^*Casp8*^*ΔE385/ΔE385*^ mice. Bars, mean + SD. *P* values (unpaired, two-tailed *t* test) ****p < 0.0001.

As zVAD itself can promote complex II formation, we wondered whether *Casp8*^Δ*E385/ΔE385*^ cells still showed enhanced complex II assembly in the absence of zVAD. Thus, we stimulated wild-type and *Casp8*^Δ*E385/ΔE385*^ MDFs with TNF-α/CHX. We found that TNF-α/CHX treatment can still induce increased interaction of caspase-8, FADD and RIPK3 with RIPK1 in *Casp8*^Δ*E385/ΔE385*^ cells instead of the WT cells (Fig. 3G), suggesting that caspase-8 cleavage can negatively regulated complex II assembly. Besides, we also found increased phosphorylation of RIPK1, RIPK3 and decreased caspase-3 cleavage in TNF-α/CHX treated *Casp8*^Δ*E385/ΔE385*^ MDFs, which also indicated that CASP8(ΔE385) switched TNF-α/CHX induced apoptosis to necroptosis (Fig. 3G). Prior evidence showed that stimulation of the Toll-like receptor (TLR) and an IAP inhibitor, can also trigger complex II assembly [15, 65, 66]. Thus, we treated BMDMs with TLR4 agonist LPS plus BV6 to induce complex II formation. Consistently, LPS/BV6 treatment also induced markedly enhanced complex II formation in *Casp8*^Δ*E385/ΔE385*^ BMDMs compared with WT counterpart (Fig. S3D). Collectively, these results demonstrate that CASP8(ΔE385) functions as a scaffold to promote complex II formation in order that the recruitment of RIPK3-caspase-8-FADD and RIPK1-RIPK3 cascade phosphorylation were significantly increased and prolonged, which results in excess necroptosis in *Casp8*^Δ*E385/ΔE385*^ cells. In addition, TNF-α-induced lethal systemic inflammatory syndrome has been wildly recognized as a mouse model to confirm necroptosis in vivo [7, 23]. Therefore, we tested whether CASP8(ΔE385) affects the lethal SIRS model in *Casp8*^Δ*E385/ΔE385*^ mice. In comparison to WT, *Casp8*^Δ*E385/ΔE385*^ mice showed significantly sensitized death accompanied by severe hypothermia (Figs. 4A, B). Furthermore, *Ripk3*^*−/−*^*Casp8*^Δ*E385/ΔE385*^ and *Ripk1*^*K45A/K45A*^*Casp8*^Δ*E385/ΔE385*^ mice were protected to a large extent from the lethal shock (Figs. 4A, B). Moreover, to examine whether caspase-8 cleavage suppresses necroptosis in macrophages in vivo, WT, *Casp8*^Δ*E385/ΔE385*^ and *Ripk1*^*+/−*^*Ripk3*^*−/−*^*Casp8*^Δ*E385/ΔE385*^ mice were pretreated with zVAD followed by challenging with LPS administration. After 24 h, the CD11b^+^F4/80^+^ intraperitoneal macrophages (PMs) were detected by flow cytometry. The CD11b^+^F4/80^+^ PMs harvested from *Casp8*^Δ*E385/ΔE385*^ mice were dramatically decreased compared to those observed in WT mice after the LPS plus zVAD treatment, and the excessive peripheral macrophages loss in *Casp8*^Δ*E385/ΔE385*^ mice was largely protected in *Ripk1*^*+/−*^*Ripk3*^*−/−*^*Casp8*^Δ*E385/ΔE385*^ mice (Figs. 4C, D). Collectively, these data reveal that caspase-8 cleavage is essential for suppressing RIPK1-RIPK3-MLKL-mediated necroptotic death in vitro and in vivo.

### *Casp8*^Δ*E385/ΔE385*^*Ripk3*^*−/−*^ mice develop serious lymphopenia and myeloid bias but prevent postnatal lethality in *Ripk1*^*−/−*^ mice

**Fig. 5:**
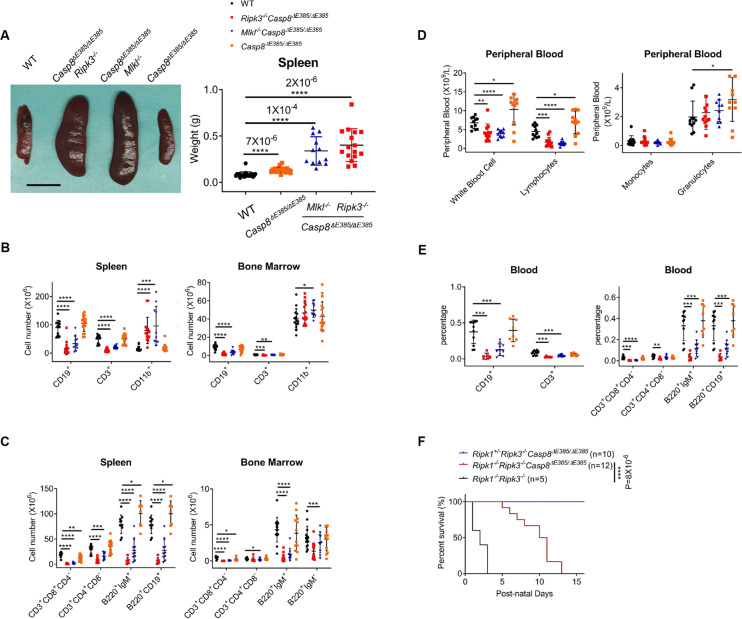
*Casp8*^*ΔE385/ΔE385*^*Ripk3*^*−/−*^ mice develop serious lymphopenia and myeloid bias but prevent the postnatal lethality of *Ripk1*^*−/−*^ mice. **A** Spleens images (15 week) (left) and total spleen weight (14- to 17-week old) (right) of the indicated genotype mice. Scale bar, 1 cm. Bars, mean ± SD. *P* values above the asterisk (unpaired, two-tailed *t* test) ****p < 0.0001. **B** The immunocytes cell number in spleen and bone marrow (per tibia and femur) of 14- to 17-week old mice. Bars, mean ± SD. *P* values (unpaired, two-tailed *t* test) *p < 0.05, **p < 0.01, ***p < 0.001, ****p < 0.0001. **C** The B cell and T cell subsets cellularity in spleen and bone marrow (per tibia and femur) of 14- to 17-week old mice. Bars, mean ± SD. *P* values (unpaired, two-tailed *t* test) *p < 0.05, **p < 0.01, ***p < 0.001, ****p < 0.0001. **D** The absolute cell number of the white blood cells and their subsets in the peripheral blood of 14- to 17-week old mice. Bars, mean ± SD. *P* values (unpaired, two-tailed *t* test) *p < 0.05, **p < 0.01, ***p < 0.001, ****p < 0.0001. **E** The B cell and T cell subsets cellularity in the peripheral blood of 14- to 17-week old mice. Bars, mean ± SD. *P* values (unpaired, two-tailed *t* test) **p < 0.01, ***p < 0.001, ****p < 0.0001. **F** Mouse survival curve of the given genotypes after birth. *P* values alongside the asterisk, two-sided Log-rank (Mantel-Cox) test, ****p < 0.0001.

*Casp8*^*−/−*^*Ripk3*^*−/−*^ and *Casp8*^*−/−*^*Mlkl*^*−/−*^ mice [11], characterized by splenomegaly with a marked accumulation of CD3^+^CD4^−^CD8^−^B220^+^ T cells, resemble the deficiency of FAS ligand (FasL, CD95L) [28] or FAS (CD95) [29, 30] in mice or the autoimmune lymphoproliferative syndrome (ALPS) in humans [31]. To investigate the role of caspase-8 auto-cleavage in this disease, we generated *Casp8*^Δ*E385/ΔE385*^*Ripk3*^*−/−*^ and *Casp8*^Δ*E385/ΔE385*^*Mlkl*^*−/−*^ mice by crossing *Casp8*^Δ*E385/ΔE385*^ mice to *Ripk3*^*−/−*^ or *Mlkl*^*−/−*^ background. Remarkably, *Casp8*^Δ*E385/ΔE385*^*Ripk3*^*−/−*^ and *Casp8*^Δ*E385/ΔE385*^*Mlkl*^*−/−*^ mice develop severe lymphopenia characterized by fewer lymphocytes in multiple organs (Figs. 5B, D, and S4A, B). Although *Casp8*^Δ*E385/ΔE385*^ mice developed slight splenomegaly and CD8^+^ T cell lymphopenia in the spleen, *Casp8*^Δ*E385/ΔE385*^*Ripk3*^*−/−*^ and *Casp8*^Δ*E385/ΔE385*^*Mlkl*^*−/−*^ mice developed more severe splenomegaly and showed a dramatically decreased percentage of B cells (CD19^+^) and T cells (CD3^+^) as well as an increased percentage of myeloid-derived cells (CD11b^+^) in the spleen and bone marrow (Fig. 5A, and S4A, B). This result indicates the possibility of lymphopenia and myeloid bias/myeloproliferative disease in these mice. Next, we counted the absolute cell number and observed a plummeted number of B cells (CD19^+^) and T cells (CD3^+^) in the spleen and bone marrow (Fig. 5B). The macrophages and granulocytes (CD11b^+^) were rapidly increased in the spleen but were approximately normal in the bone marrow (Fig. 5B). These results characterized the lymphopenia and myeloid bias disease but excluded the possibility of myeloproliferative disease in these mice. Furthermore, we analyzed the subsets of B cells and T cells in the spleen and bone marrow. Consistent with the percentage results (Fig. S4C), immature and mature B cells (B220^+^IgM^+^/B220^hi^CD19^hi^), progenitor B cells (pro-B) and precursor B cells (pre-B) (B220^+^IgM^−^/B220^low^CD19^low^), and CD8^+^ T cells showed a dramatic decrease in the bone marrow of *Casp8*^Δ*E385/ΔE385*^*Ripk3*^*−/−*^ and *Casp8*^Δ*E385/ΔE385*^*Mlkl*^*−/−*^ mice (Fig. 5C and S4D). In the spleen, the absolute cell numbers of peripheral B cells and T cells were also decreased (Fig. 5C). Taken together, these data showed that the *Casp8*^Δ*E385/ΔE385*^*Ripk3*^*−/−*^ and *Casp8*^Δ*E385/ΔE385*^*Mlkl*^*−/−*^ mice develop severe myeloid bias and lymphopenia in the spleen and bone marrow.

To further confirm the presence of lymphopenia in these mice, we next analyzed the peripheral blood. Indeed, *Casp8*^Δ*E385/ΔE385*^*Ripk3*^*−/−*^ and *Casp8*^Δ*E385/ΔE385*^*Mlkl*^*−/−*^ mice showed a distinct decrease in white blood cell (WBC) and lymphocytes but normal numbers of monocytes and granulocytes in the blood (Fig. 5D). Interestingly, *Casp8*^Δ*E385/ΔE385*^ mice showed a minor increase in the WBC and lymphocyte counts (Fig. 5D). Furthermore, the total levels of B cells (CD19^+^), T cells (CD3^+^) as well as mature B cells (B220^+^IgM^+^/B220^+^CD19^+^) and T cells subsets (CD3^+^CD4^+^CD8^−^/CD3^+^CD8^+^CD4^−^) sharply decreased in the blood of *Casp8*^Δ*E385/ΔE385*^*Ripk3*^*−/−*^ and *Casp8*^Δ*E385/ΔE385*^*Mlkl*^*−/−*^ mice (Fig. 5E). Collectively, these results demonstrate that caspase-8 cleavage associated with RIPK3 or MLKL plays a critical role in maintaining immune cell homeostasis.

In addition, *Ripk3*^*−/−*^*Casp8*^*−/−*^ can rescue the postnatal lethality of *Ripk1*^*−/−*^ mice by inhibiting both apoptosis and necroptosis [67, 68]. Therefore, we examined whether CASP8(ΔE385) combined with the ablation of *Ripk3* contributed to the perinatal death of *Ripk1*^*−/−*^ mice. We generated *Ripk1*^*−/−*^*Ripk3*^*−/−*^*Casp8*^Δ*E385/ΔE385*^ mice by intercrossing *Ripk1*^*+/−*^*Ripk3*^*−/−*^*Casp8*^Δ*E385/ΔE385*^ mice. *Ripk1*^*−/−*^*Ripk3*^*−/−*^*Casp8*^Δ*E385/ΔE385*^ mice survived normally at birth; however, they were runted with apparent focal cutaneous lesions and scaling on the skin, and eventually died around two weeks after birth (Fig. 5F and S5A). These data suggest that caspase-8 cleavage mediated apoptosis combined with RIPK3 dependent necroptosis was partially responsible for the perinatal lethality of RIPK1 deficiency mice. This observation further confirmed that Caspase-8 cleavage is essential for apoptosis during development.

### Halve the expression of RIPK1 rescues transplantable lymphopenia in *Casp8*^Δ*E385/ΔE385*^*Ripk3*^*−/−*^ mice

**Fig. 6:**
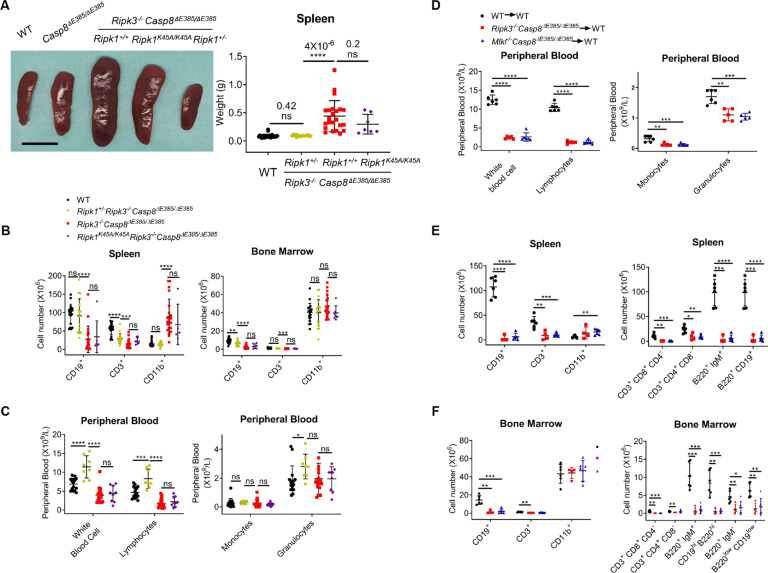
Halving the RIPK1 dosage rescues transplantable lymphopenia and myeloid bias in *Ripk3*^*−/−*^*Casp8*^*ΔE385/ΔE385*^ mice. **A** Spleen images (12 week) (left) and total spleen weight (14–17 week) (right) showed normal sized spleen in the *Ripk1*^*+/−*^ *Ripk3*^*−/−*^ *Casp8*^*ΔE385/ΔE385*^ mice. Scale bar, 1 cm. Bars, mean ± SD. *P* values above the asterisk (unpaired, two-tailed *t* test) ****p < 0.0001; ns, no significance. **B** The absolute cell number of indicated immunocytes in spleen and bone marrow (per tibia and femur) of 14- to 17-week old age matched mice. Bars, mean ± SD. *P* values (unpaired, two-tailed *t* test) **p < 0.01, ***p < 0.001, ****p < 0.0001; ns, no significance. **C** The cell number of white blood cells and their subsets in the peripheral blood of 14- to 17-week old mice. Bars, mean ± SD. *P* values (unpaired, two-tailed *t* test) *p < 0.05, ***p < 0.001, ****p < 0.0001; ns, no significance. **D** The absolute cell number and percentage of white blood cells and their subsets in the peripheral blood of 6-month old recipients. Bars, mean ± SD. *P* values (unpaired, two-tailed *t* test) *p < 0.05, **p < 0.01, ***p < 0.001, ****p < 0.0001. **E** The absolute cell number of the immunocytes and their subsets in the spleen of 6-month old recipients. Bars, mean ± SD. *P* values (unpaired, two-tailed *t* test) *p < 0.05, **p < 0.01, ***p < 0.001, ****p < 0.0001. **F** The absolute cellularity of the immunocytes and their subsets in the bone marrow per tibia and femur of 6-month old recipients. Bars, mean ± SD. *P* values (unpaired, two-tailed *t* test) *p < 0.05, **p < 0.01, ***p < 0.001.

Although *Ripk1*^*−/−*^*Ripk3*^*−/−*^*Casp8*^Δ*E385/ΔE385*^ mice did not survive to adulthood, we found that *Ripk1*^*+/−*^*Ripk3*^*−/−*^*Casp8*^Δ*E385/ΔE385*^ mice were viable beyond weaned and fertile. Furthermore, splenomegaly in *Ripk3*^*−/−*^*Casp8*^Δ*E385/ΔE385*^ mice was largely suppressed in *Ripk1*^*+/−*^*Ripk3*^*−/−*^*Casp8*^Δ*E385/ΔE385*^ mice (Fig. 6A). Consistently, myeloid bias and lymphopenia in the spleen and lymphopenia in the bone marrow were also significantly relieved in *Ripk1*^*+/−*^*Ripk3*^*−/−*^*Casp8*^Δ*E385/ΔE385*^ mice compared to those in *Ripk3*^*−/−*^*Casp8*^Δ*E385/ΔE385*^ mice (Fig. 6B). In addition, *Ripk1*^*+/−*^*Ripk3*^*−/−*^*Casp8*^Δ*E385/ΔE385*^ mice exhibited normal immature and mature B cells (B220^+^IgM^+^/B220^hi^CD19^hi^) in the bone marrow (Fig. S5B, D). Importantly, the complete blood count results showed increased WBC and lymphocyte in the peripheral blood of *Ripk1*^*+/−*^*Ripk3*^*−/−*^*Casp8*^Δ*E385/ΔE385*^ mice compared to WT mice (Fig. 6C), suggesting that lymphopenia and myeloid bias in *Ripk3*^*−/−*^*Casp8*^Δ*E385/ΔE385*^ mice were largely alleviated by halving RIPK1 dosage.

To further test whether RIPK1 kinase activity contributed to lymphopenia in *Ripk3*^*−/−*^*Casp8*^Δ*E385/ΔE385*^ mice, we generated *Ripk1*^*K45A/K45A*^*Ripk3*^*−/−*^*Casp8*^Δ*E385/ΔE385*^ mice and observed that *Ripk1*^*K45A/K45A*^*Ripk3*^*−/−*^*Casp8*^Δ*E385/ΔE385*^ mice showed lymphopenia and myeloid bias similar to *Ripk3*^*−/−*^*Casp8*^Δ*E385/ΔE385*^ mice (Figs. 6A–C and S5B–D). Collectively, these results demonstrate that RIPK1 dosage-dependent and RIPK1 kinase-independent scaffold function contributes to lymphopenia and myeloid bias in *Ripk3*^*−/−*^*Casp8*^Δ*E385/ΔE385*^ mice.

Next, we asked whether lymphopenia was intrinsic to *Ripk3*^*−/−*^*Casp8*^Δ*E385/ΔE385*^ and *Mlkl*^*−/−*^*Casp8*^Δ*E385/ΔE385*^ hematopoietic stem cells (HSCs). The complete bone marrow of *Ripk3*^*−/−*^*Casp8*^Δ*E385/ΔE385*^ and *Mlkl*^*−/−*^*Casp8*^Δ*E385/ΔE385*^ mice was transplanted into lethally irradiated syngeneic WT recipients (Fig. S6A). After hematopoiesis was reestablished, we observed that the mice receiving *Mlkl*^*−/−*^*Casp8*^Δ*E385/ΔE385*^ bone marrow developed splenomegaly, whereas the spleen of *Ripk3*^*−/−*^*Casp8*^Δ*E385/ΔE385*^ recipients showed no difference (Fig. S6B). In the peripheral blood, the *Ripk3*^*−/−*^*Casp8*^Δ*E385/ΔE385*^ and *Mlkl*^*−/−*^*Casp8*^Δ*E385/ΔE385*^ recipients showed leucopenia and deficiency in every WBC subset (Fig. 6D), while the red blood cells, platelets, and hemoglobin levels showed a minor decrease (Fig. S6C). Consistently, lymphopenia was recapitulated in the *Ripk3*^*−/−*^*Casp8*^Δ*E385/ΔE385*^ and *Mlkl*^*−/−*^*Casp8*^Δ*E385/ΔE385*^ recipients characterized by CD8^+^ T cell deficiency in blood and decreased B cells, T cells, and their subsets in the spleen, bone marrow, and blood (Figs. 6E, F and S6D). Collectively, caspase-8 cleavage together with RIPK3 or MLKL suppresses the intrinsic lymphopenia of hematopoietic stem cells.

## Discussion

Caspase-8 is a key regulator of apoptosis and necroptosis, as well as the inflammatory response through its dimerization and enzymatic activity [1, 5, 16]. The auto-cleavage activity of Caspase-8 has also been shown to be involved in mediating apoptosis and regulating inflammation [13].

In this study, we demonstrated that CASP8(ΔE385) not only compromised Fas-induced apoptosis and switched TNF-a induced apoptosis to necroptosis but also promoted necroptosis both in vitro and in vivo. However, in contrast to the embryonic lethality observed in caspase-8 deficient [49] or with catalytically inactive caspase-8 mice [6, 8], *Casp8*^Δ*E385/ΔE385*^ mice survived normally, suggesting that primarily caspase-8 catalytic activity rather than caspase-8 cleavage contributes to the suppression of RIPK3-MLKL mediating necroptosis during embryo development.

In the current study, we observed that caspase-8 cleavage between the large and small subunits was increased under TNF-α/Smac/zVAD (Fig. 1B), which is consistent with results from TNF-α plus zVAD stimulation in previous studies [69, 70]. It has also been suggested that pro-caspase-8 and activated caspase-8 have divergent substrate specificities [71, 72], and the substrate specificities of procaspase-8 change when it heterodimerizes with cFLIP_L_ in complex II [42]. It has also been shown that Z-VAD-FMK is less efficacious at inhibiting the caspase-8 homodimer than the caspase-8/cFLIP_L_ heterodimer [73]. Thus, one possible explanation is that zVAD promotes complex II formation, but its ability to inhibit the catalytic activity of pro-caspase-8 is not as efficacious as to inhibit the activated caspase-8, which contributes to more caspase-8 auto-processing.

Earlier studies have demonstrated that perinatal death in *Ripk1*^*−/−*^ mice is prevented by co-ablation of FADD/caspase-8 dependent apoptosis and RIPK3/MLKL dependent necroptosis [67, 68]. Here, we generated *Ripk1*^*−/−*^*Ripk3*^*−/−*^*Casp8*^Δ*E385/ΔE385*^ mice that died around two weeks to strongly prolong the survival of *Ripk1*^*−/−*^ *Ripk3*^*−/−*^ mice. During the manuscript preparation, a recent paper reported that *Fadd*^*−/−*^*Mlkl*^*−/−*^*Casp8*^*DA/DA*^ also died around two weeks after birth due to the exacerbation of inflammation [13], suggesting that caspase-8 exhibits a FADD-independent inflammatory function that is inhibited by caspase-8 cleavage. Therefore, whether lethal inflammation in *Ripk1*^*−/−*^*Ripk3*^*−/−*^*Casp8*^Δ*E385/ΔE385*^ mice can be prevented by the additional ablation of caspase-1 as *Fadd*^*−/−*^*Mlkl*^*−/−*^*Casp8*^*DA/DA*^ mice remain to be determined.

The role of caspase-8, RIPK3, and MLKL in non-programmed cell death has been reported to regulate lymphadenopathy [11], lymphoproliferation [25] and immunodeficiency [26, 33]. We demonstrated an unexpected role of caspase-8 auto-cleavage cooperating with RIPK3 or MLKL and RIPK1 in lymphopenia regulation. Unlike *Casp8*^*−/−*^*Ripk3*^*−/−*^ and *Casp8*^*−/−*^*Mlkl*^*−/−*^ mice, which resemble the human ALPS [11] and impair cytokine response [33], we found that *Casp8*^Δ*E385/ΔE385*^*Ripk3*^*−/−*^ and *Casp8*^Δ*E385/ΔE385*^*Mlkl*^*−/−*^ mice develop hematopoietic cell-intrinsic lymphopenia and myeloid bias (Figs. 5, 6). We observed that the circulating mature B cells (B220^+^IgM^+^) and T cells in the peripheral blood and spleen of *Casp8*^Δ*E385/ΔE385*^*Ripk3*^*−/−*^ and *Casp8*^Δ*E385/ΔE385*^*Mlkl*^*−/−*^ mice were dramatically decreased. This can be explained by decreased immature and mature B cells and T cells in the bone marrow (Fig. 5B–E). Moreover, Lymphopenia and myeloid bias in *Ripk3*^*−/−*^*Casp8*^Δ*E385/ΔE385*^ mice were largely suppressed in *Ripk1*^*+/−*^*Ripk3*^*−/−*^*Casp8*^Δ*E385/ΔE385*^ mice but not in *Ripk1*^*K45A/K45A*^*Ripk3*^*−/−*^*Casp8*^Δ*E385/ΔE385*^ mice, revealing a previously unknown role of the dosage of RIPK1 instead of RIPK1 kinase activity administered to the mice in maintaining immune cell homeostasis in *Ripk3*^*−/−*^*Casp8*^Δ*E385/ΔE385*^ mice.

In this study, we identified the phenotypes of *Casp8*^Δ*E385/ΔE385*^ mice which resemble those of the *Casp8*^*DA/DA*^ mice from a recent study [13]. Moreover, we also confirmed the enzymatic activity of CASP8(ΔE385) by examining caspase-3 cleavage in thymocytes with FasL treatment [46]. We found that *Casp8*^Δ*E385/ΔE385*^ thymocytes showed comparable level of caspase-3 cleavage and cell death to that in *Casp8*^*D387A/D387A*^ thymocytes after FasL treatment (Figs. 2A and S2A), which indicated CASP8(ΔE385) has comparable enzymatic activity as caspase-8(D387A) [46]. However, we still cannot exclude the possibility that deletion of one amino acid in caspase-8 alters other caspase-8-mediated cellular signaling, therefore, whether E385 deletion influences other functions of caspase-8, in addition to its auto-cleavage, needs to be investigated further.

In summary, caspase-8 auto-cleavage plays an important role in regulating cell death and immune cell homeostasis, that is, mediating apoptosis, suppressing necroptosis, and protecting from lymphopenia (Fig. S7). Although CASP8(ΔE385) is sufficient to suppress necroptosis during embryonic development, CASP8(ΔE385) can induce excessive necroptosis by switching apoptosis to necroptosis and promoting complex II assembly and stabilization. Accordingly, *Casp8*^Δ*E385/ΔE385*^ mice are strongly sensitized to TNF-α induced necroptosis in vivo. In addition, *Casp8*^Δ*E385/ΔE385*^*Ripk3*^*−/−*^ and *Casp8*^Δ*E385/ΔE385*^*Mlkl*^*−/−*^ mice develop severe lymphopenia that can be prevented by reducing the RIPK1 dosage by half, not by RIPK1 kinase inactive mutant. This indicates that caspase-8 cleavage cooperating RIPK3/MLKL to regulate RIPK1 scaffold-dependent but RIPK1 kinase-independent function contributes to the maintenance of immune cell homeostasis. The exact signaling pathway and mechanism require further investigation.

## Materials and methods

### Mice

All mice utilized in this study were C57BL/6 background and housed in a specific pathogen-free (SPF) facility. Both male and female mice were used in this study. For all studies mice were age- and sex-matched. *Ripk1*^*+/−*^, *Ripk3*^*−/−*^, *Ripk1*^*K45A/K45A*^ and *Mlkl*^*−/−*^ mouse lines have been described previously [74, 75]. *Casp8*^Δ*E385/ΔE385*^ mice were generated by CRISPR-Cas9 mutation system (Bioray Laboratories Inc., Shanghai, China). Three adjacent nucleotides AAG was removed in the exon 8 of the *Casp8* gene locus resulted in the deletion of Glutamic acid (Glu, E) in 385 position of caspase-8 protein sequence. The *Casp8* (ID: 12370) gene region corresponds to genomic position chr1: 58844689-58844691. *Casp8*^Δ*E385/ΔE385*^ mice genotyping primers: 5′-CAGAGGCTCTGAGTAAGACC-3′ and 5′-CTGAGGACATCTTTCCCTCAG-3′ amplified 506 bp DNA fragments for sequencing. Additional information is provided upon request. Animal experiments were conducted in accordance with the guidelines of the Institutional Animal Care and Use Committee of the Institute of Nutrition and Health, Shanghai Institutes for Biological Sciences, University of Chinese Academy of Sciences.

### Isolation and culture of thymocytes, mouse dermal fibroblasts (MDFs) and bone marrow derived macrophages (BMDMs)

Both male and female mice were used to generate MDFs and BMDMs. MDFs were separated from the skin of newborn mice (P0-P1), and cultured in DMEM medium (SH30243.01B, HyClone) supplemented with 10% of Fetal Bovine Serum (04-001-1 A, Bioind) and 1% of penicillin/streptomycin (15140122, Gibco). BMDMs were isolated from the bone marrow of mouse femurs and tibias followed by inducing to differentiate in vitro. Bone marrow cells were cultured for 7 days in RPMI-1640 medium (SH30809.01B, HyClone) containing 10% of Fetal Bovine Serum (04-001-1 A, Bioind) and 1% of penicillin/streptomycin (15140122, Gibco) and 50 ng/ml M-CSF (AF-315-02, PeproTech), and medium was refreshed each 3 days. Cells were cultivated at 37 °C with 5% CO_2_.

### Cell death stimulation and cell survival assay

MDFs were plated in 96-well plates 12 h before stimulation at a concentration of 1 × 10^4^ cells per well. For TNF-α induced apoptosis and necroptosis stimulation, MDFs were treated with TNF-α (20 ng/ml) (T) for 10 h, TNF-α (20 ng/ml) + Smac (1 μM) (TS), TNF-α + Smac +Necrostatin-1 (30 μM) (TSN), TNF-α + Smac+zVAD (20 μM) (TSZ), TNF-α + Smac+zVAD+Nec-1 (TSZN) for 6.45 h, TNF-α(20 ng/ml)+CHX (20 μg/ml) (TC), TNF-α + CHX + Necrostatin-1 (30 μM)(TCN), TNF-α + CHX + zVAD (20 μM) (TCZ), and TNF-α + CHX + zVAD+Nec-1 (TCZN) for 4.45 h. For GSK’872 induced apoptosis, MDFs were treated with GSK’872 in concentration of 3 μM, 6 μM and 10 μM for 10 h, respectively.

BMDMs were plated in 96-well plates 12 h before stimulation at a concentration of 2 × 10^4^ cells per well. For TNF-α, LPS and poly(I:C) induced apoptosis and necroptosis stimulation, BMDMs were treated with TNF-α (20 ng/ml) + Smac (1 μM) + zVAD (20 μM) (TSZ), TNF-α + Smac + zVAD + Nec-1 (30 μM) (TSZN), LPS (100 ng/ml) (L), LPS (100 ng/ml) + zVAD (20 μM) (LZ), LPS + zVAD + Nec-1 (30 μM) (LZN), poly(I:C) (100 μg/ml) (P), poly(I:C) (100 μg/ml) + zVAD (20 μM) (PZ), poly(I:C) + zVAD + Nec-1 (30 μM) (PZN) for 3 h.

Thymocytes were plated in 96-well plates 12 h before stimulation at a concentration of 4 × 10^4^ cells per well. For Fas-induced apoptosis, thymocytes were treated with anti-Fas antibody (Jo-2, 100 ng/ml) + CHX (30 μg/ml) (FC) for 12 h, 15 h, 18 h and 21 h, respectively.

Cell survival was determined using the CellTiter-Glo Luminescent Cell Viability Assay kit (G7572, Promega) and the luminescence was recorded with a microplate luminometer (5300170, Thermo Scientific).

### Cell death analysis by western blot (WB) and complex II immunoprecipitation

MDFs were plated in 6-cm dishes 12 h before stimulation at a concentration of 2 × 10^6^ cells per dish. For TNF-α induced apoptosis and necroptosis stimulation, MDFs were treated with TNF-α (40 ng/ml) + Smac (2 μM) (TS), TNF-α (20 ng/ml) + Smac (1 μM) + zVAD (20 μM) (TSZ), TNF-α (40 ng/ml) + CHX (40 μg/ml) (TC), TNF-α (40 ng/ml) + CHX (40 μg/ml) + zVAD (20 μM) (TCZ) for the indicated time. For GSK’872 induced apoptosis, MDFs were treated with GSK’872 (20 μM) for the indicated time.

BMDMs were plated in 6-cm dishes 12 h before stimulation at a concentration of 2 × 10^6^ cells per dish. For LPS induced necroptosis stimulation, BMDMs were treated with LPS (200 ng/ml) (L), LPS + zVAD (40 μM) (LZ) for 6 h.

Cells were harvested after stimulation, washed with PBS and lysates with RIPA lysis buffer (50 mM Tris-HCl (pH7.4), 150 mM NaCl, 2 mM EDTA, 1% NP-40, 0.1% SDS, Protease inhibitor Cocktail (4693132001, Roche), Phosphatase inhibitor Cocktail 3 (P0044-1ML, Sigma)) for 30–45 min on ice. The lysates were centrifuged for 20 min at 13,200 g, 4 °C, quantified by BCA kit (P0010S, Beyotime) and then mixed with SDS sample buffer (250 mM Tris-Cl (PH 6.8), 10% SDS, 30% Glycerol, 5% β-mercapitalethanol, 0.02% Bromophenol blue) followed by boiling at 100 °C for 10 min. The proteins were separated by SDS-PAGE, and then transferred to PVDF membrane (IPVH00010, Millipore) at 110 v for 3 h. Membranes were blocked with 5% skimmed milk in PBST 0.1% for 1 h. Membranes were washed three times with PBST 0.1% for 7 min. Membranes were incubated in PBST 0.1% containing primary antibodies at 4 °C overnight. The proteins were detected by chemiluminescent substrate (34080, Thermo Scientific) using Tanon 5200 Multi Luminescent Imaging Workstation (Tanon). For mouse tissue protein extraction, the indicated tissues were ground into powder by pestle and mortar with liquid nitrogen, and the protein was extracted with RIPA lysis buffer followed by centrifugation, quantification, SDS-PAGE and transmembrane as above. For GSK’872 induced apoptosis detection in Fig. 2B, the MDFs were harvested by RIPA lysis buffer with 6 M Urea.

For complex II immunoprecipitation (IP), cells were lysed with lysis buffer (20 mM Tris-HCl (pH 7.5), 1% Triton X-100, 0.2% NP-40, 120 mM NaCl, 0.27 M sucrose, 1 mM EDTA, 1 mM EGTA, 50 mM NaF, 10 mM β-glycerophosphate, 5 mM sodium pyrophosphate, 2 mM PMSF, Protease inhibitor Cocktail (4693132001, Roche), Phosphatase inhibitor Cocktail 3 (P0044-1ML, Sigma)). Cell lysates were overnight incubated with 1 μg of anti-RIPK1 (610459, BD Biosciences) at 4 °C followed by 4 h incubation with 50 μl of Protein A agarose (16-125, Millipore). Beads were washed and proteins were eluted with 2X SDS sample buffer followed by boiling at 100 °C for 10 min.

The primary antibodies used for western blot: anti-RIPK1 (610459, BD Biosciences), anti-phosphorylated RIPK1 (31122 S, Cell Signaling Technology), anti-RIPK3 (2283, Prosci), anti-phosphorylated RIPK3 (ab195117, Abcam), anti-caspase-8 (ALX-804-447-C100, Enzo Life Science), anti-cleaved caspase-8 (9429 S, Cell Signaling Technology), anti-caspase-8 (4927 S, Cell Signaling Technology), anti-MLKL (AP14272b, Abgent), anti-phosphorylated MLKL (ab196436, Abcam), anti-FADD (ab124812, Abcam), anti-PARP (9542 S, Cell Signaling Technology), anti-caspase-3 (9662 S, Cell Signaling Technology), anti-cleaved caspase-3 (9661 S, Cell Signaling Technology), anti-β-actin (3779, Prosci), anti-GAPDH (G9545, Sigma).

### Anti-Fas induced thymocytes apoptosis analyzed by flow cytometry and western blot

Both male and female mice were used to harvest thymocytes. Thymocytes were harvested from wild-type and *Casp8*^Δ*E385/ΔE385*^ mice of 1-month old, and cultured in DMEM medium (SH30243.01B, HyClone) supplemented with 10% of heat-inactivated Fetal Bovine Serum (04-001-1 A, Bioind), 1% of penicillin/streptomycin (15140122, Gibco), 200 mM L-glutamine (25030-081, Gibco), 1X MEM non-essential amino acids (NEAA) (11140-050, Gibco) and 55 mM 2-Mercaptoethanol (M6250, Sigma). Cells were cultivated at 37 °C with 5% CO_2_.

For flowcytometry analysis, thymocytes were plated in 6-well plates followed by stimulation at a concentration of 1 × 10^6^ cells per well, and thymocytes were treated with 2 μg/ml anti-Fas antibody (Jo-2, 554255, BD) for 24 h followed by staining with FITC-Annexin V and PI utilizing apoptosis detection kit (C1062L, Beyotime). After staining, cells were analyzed in cytoflex S flow cytometer (cytoflex S, Beckman Coulter). All analyses were performed using CytExpert software (CytExpert, Beckman Coulter, Inc.).

For western blot analysis, thymocytes were plated in 10-cm dish followed by stimulation at a concentration of 2 × 10^7^ cells per well, and thymocytes were treated with 1 μg/ml anti-Fas antibody (Jo-2, 554255, BD) for the indicated time followed by washing with 1XPBS and lysates with RIPA lysis buffer (50 mM Tris-HCl (pH7.4), 150 mM NaCl, 2 mM EDTA, 1% NP-40, 0.1% SDS, Protease inhibitor Cocktail (4693132001, Roche), Phosphatase inhibitor Cocktail 3 (P0044-1ML, Sigma)).

### MLKL oligomerization detection

MDFs were cultured in 6-cm dishes at a concentration of 2 × 10^6^ cells per dish and challenged by TNF-α (20 ng/ml) +Smac (1 μM) +zVAD (20 μM) for the indicated time. MDFs were harvested at different time points and lysed with non-reducing sample buffer (125 mM Tris-Cl (PH 6.8), 20% Glycerol, 0.02% Bromophenol blue) immediately. Total cell lysates were separated using SDS-PAGE, transferred to PVDF membrane (IPVH00010, Millipore), and detected with the indicated antibodies.

### Anti-Fas induced hepatocellular apoptosis and analysis of the serum and liver damage

The wild-type and *Casp8*^Δ*E385/ΔE385*^ mice of 8- to 12-week old were injected intravenously with anti-Fas antibody (Jo-2, 554255, BD) in the dose of 0.5 μg/g and their survival time was followed for 20 h. At the indicated times, their livers and peripheral blood were harvested followed by processing for histological analysis, western blot and analyzing the alanine transaminase (ALT) and aspartate transaminase (AST) levels in serum. To analyze the ALT and AST levels in serum, the peripheral blood of the indicated mice were collected in anticoagulation tube followed by centrifugation at 7000 g, 4 °C for 30 min. The serum was collected to detect ALT (3040280, Shanghai Shensuo UNF Medical Diagnostic Articles Co.) and AST (3050280, Shanghai Shensuo UNF Medical Diagnostic Articles Co.) level utilizing the kit.

### TNF-α induced mice toxicity and analysis of the body temperature

The WT, *Casp8*^Δ*E385/ΔE385*^, *Casp8*^Δ*E385/ΔE385*^*Ripk3*^*−/−*^ and *Casp8*^Δ*E385/ΔE385*^*Ripk1*^*K45A/K45A*^ mice of 8- to 16-week old were injected intravenously with TNF-α (CRT192C, Cell sciences and obtained from Dr. Yi Zhang at Shanghai Institute of Nutrition and Health, CAS) in the dose of 7 μg each mouse and their body temperature was measured every 2 h until the twelfth hour after injection.

### Flow cytometry analyses

Lymphocytes were isolated from the peripheral blood, spleen, bone marrow and lymph nodes of the indicated mice. Total cell numbers were counted using counting slides (SD-100, Nexcelom) in Cellometer Mini Automated Cell Counter (Nexcelom). Surface antigens were stained with indicated conjugated primary antibodies in the staining buffer (1 × PBS, 3% BSA, 1 mM EDTA, 0.1%NaN_3_) at 4 °C for 30 min. Antibodies used are asfollows: FITC anti-CD3 (11-0031-82, eBioscience), APC Cy7 anti-CD4 (552051, BD Biosciences), PerCp anti-CD8 (100732, Biolegend), PE anti-B220 (12-0452-83, eBioscience), APC anti-B220 (17-0452-83, eBioscience), APC anti-CD11b (17-0112-83, eBioscience), Brilliant Violet 421 anti-CD11b (562605, BD Biosciences), PE Cy7 anti-CD19 (25-0193-82, eBioscience), FITC anti-IgM (115-097-020, Jackson Laboratories), FITC anti-F4/80 (11-4801-85, eBioscience) were used for flow cytometry analysis in this study. After staining, cells were washed once with 1XPBS and immediately analyzed by in cytoflex S flow cytometer (cytoflex S, Beckman Coulter). All analyses were performed using CytExpert software (CytExpert, Beckman Coulter, Inc.).

### Analyses of CD11b^+^ F4/80^+^ peritoneal macrophages in vivo

Wild-type, *Casp8*^Δ*E385/ΔE385*^ and *Ripk1*^*+/−*^*Ripk3*^*−/−*^*Casp8*^Δ*E385/ΔE385*^ mice were injected intraperitoneally with vehicle or zVAD (20 mg/kg) 1 h before intraperitoneal injection with PBS or LPS (10 mg/kg). Animals were killed at twenty fourth hour after the first injection, resident peritoneal cells were harvested by lavage of the peritoneal cavity with 8 ml PBS. CD11b^+^F4/80^+^ peritoneal macrophages were analyzed by flow cytometry.

### Bone marrow transplantation assay

All of the recipient mice were wild type with C57BL/6 background, which received 11 Gy of total body irradiation in a split dose (550 rads) with 4-hour rest between doses using a Cesium-137 irradiator. Irradiated recipients were reconstituted by intravenous injection of 2.5 × 10^6^ bone marrow cells from femurs and tibias of the 6-week old indicated genotype mice. Recipients were sacrificed at fourth months after reconstitution.

### Whole blood count analysis

The whole peripheral blood of the indicated mice was collected in anticoagulation tube followed by diluting in EDTA buffer (0.5 M EDTA pH8.0) at a ratio of 1:1, and then diluted peripheral blood was analyzed on an auto hematology analyzer (BC-2800Vet, Mindray).

### Quantification and statistical analysis

Please refer to the figure legends for description of sample size (*n*) and statistical significance. Data were analyzed with GraphPad Prism 8.0 software using the two-tailed unpaired Student *t* test or two-sided Log-rank (Mantel-Cox) test. Bars, mean ± standard deviation (mean ± SD). Differences were considered statistically significant when the *P* < 0.05, where ∗∗∗∗*p* < 0.0001, ∗∗∗*p* < 0.001, ∗∗*p* < 0.01, ∗*p* < 0.05, ns, not significant.

## Supplementary information

Figure S1

Figure S2

Figure S3

Figure S4

Figure S5

Figure S6

Figure S7

Detailed Attribution of Authorship

Reproducibility Checklist

Merged Files

Supplemental Figure Legends

## Data Availability

The authors declare that all data supporting the findings of this study are present in the paper and/or the Supplementary Materials.
