# Supplementary figures and images for: Caspase-8 auto-cleavage regulates programmed cell death and collaborates with RIPK3/MLKL to prevent lymphopenia
Source: Cell Death Differ. 2022 Jan 21;29(8):1500–12. doi: 10.1038/s41418-022-00938-9 (PMC9345959; doi:10.1038/s41418-022-00938-9)

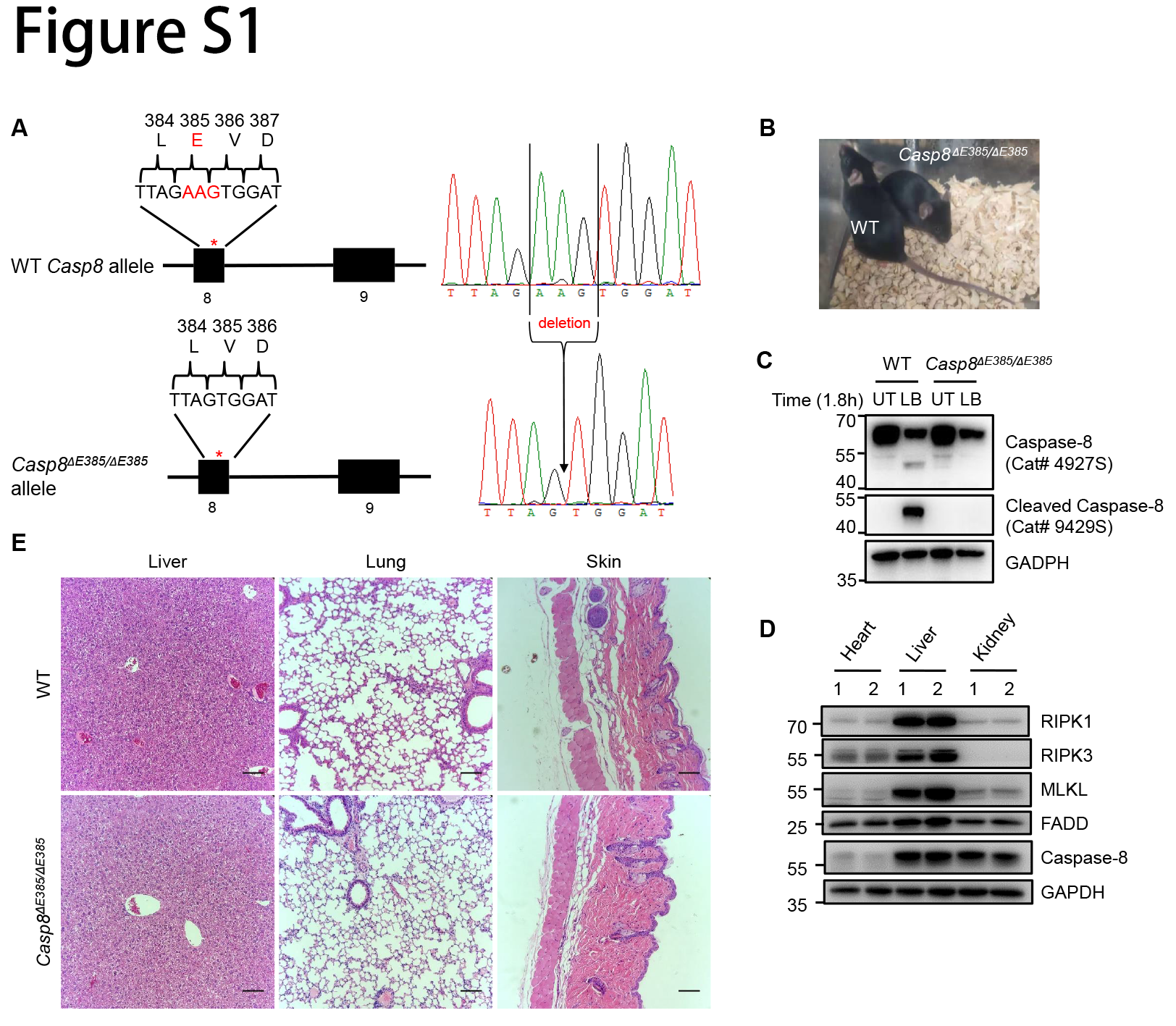

Supplement: Supplementary file 1 — Figure S1 [file 41418_2022_938_MOESM1_ESM.tif]

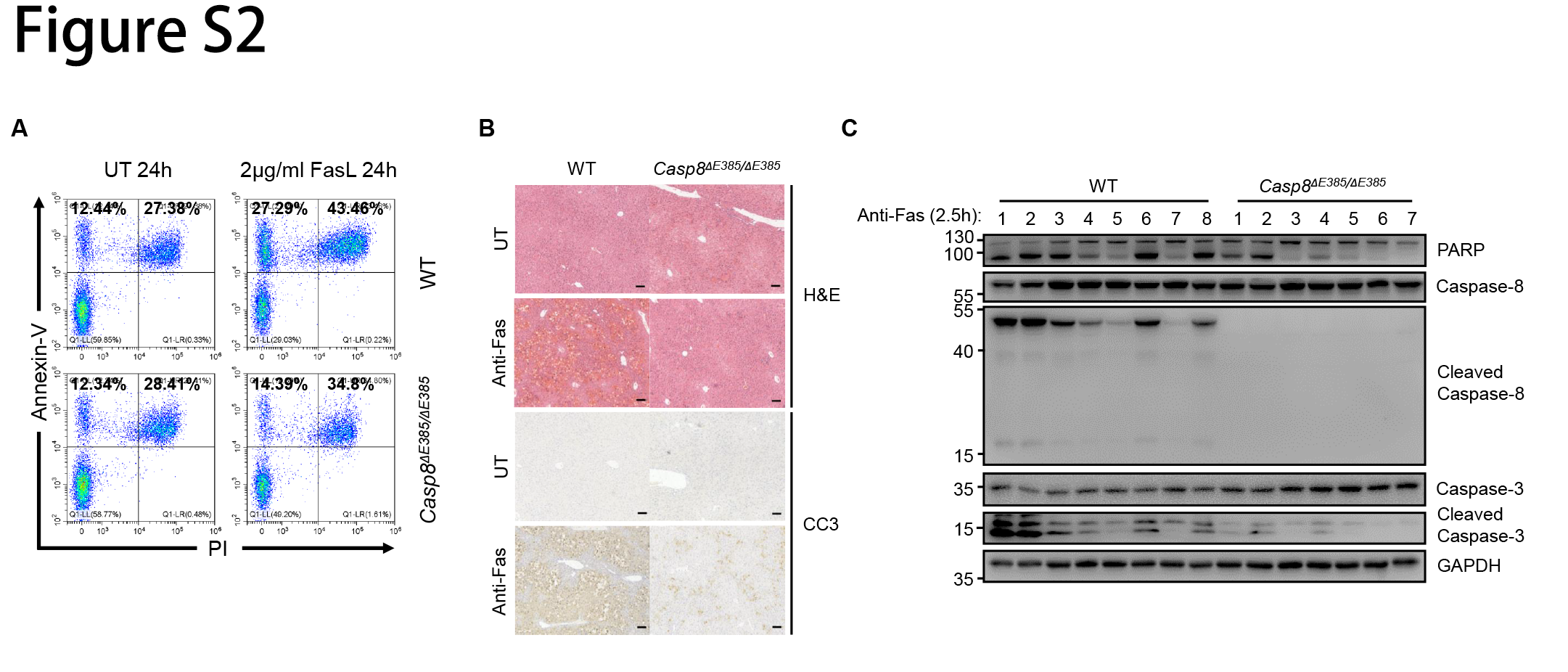

Supplement: Supplementary file 2 — Figure S2 [file 41418_2022_938_MOESM2_ESM.tif]

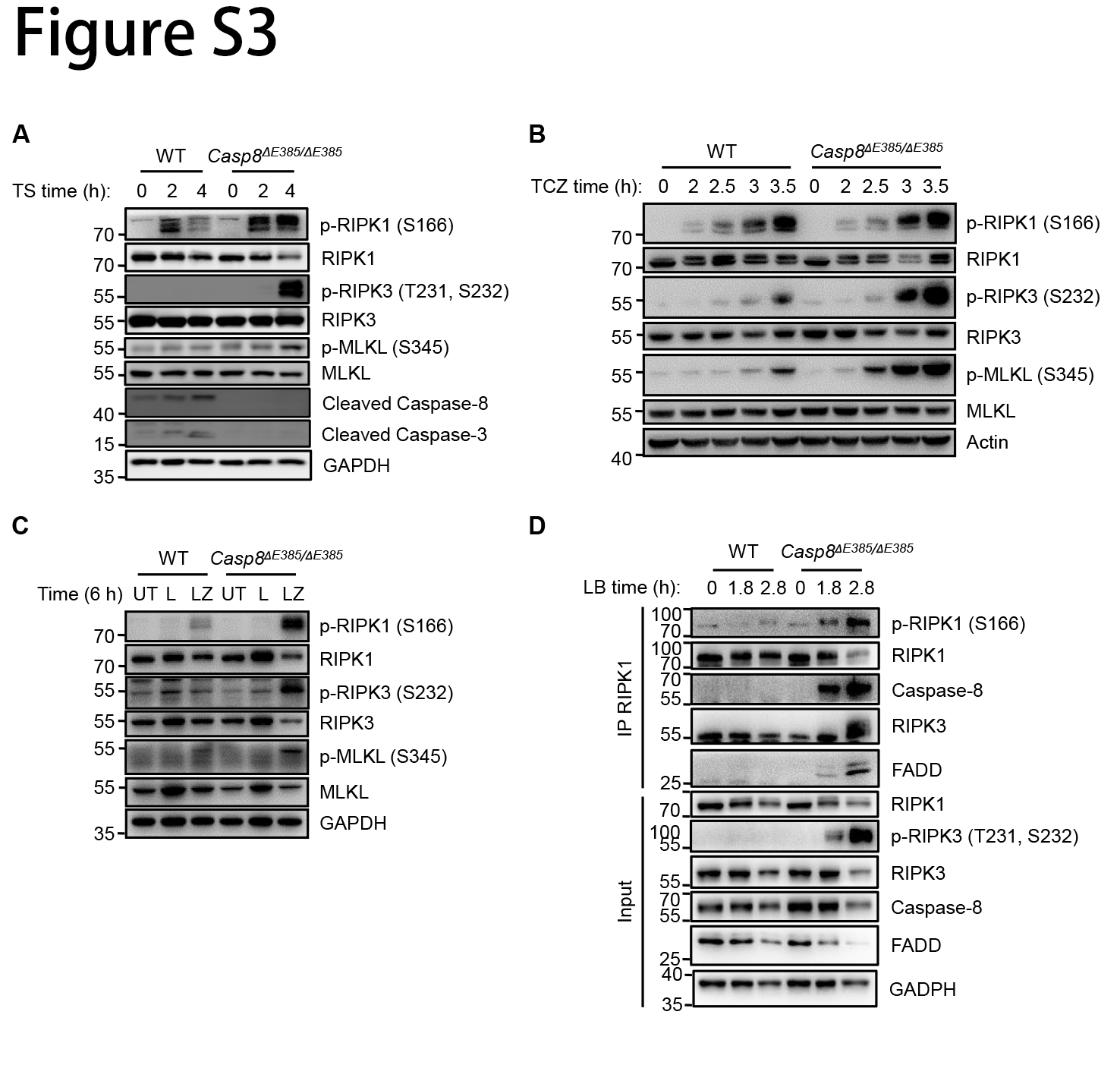

Supplement: Supplementary file 3 — Figure S3 [file 41418_2022_938_MOESM3_ESM.tif]

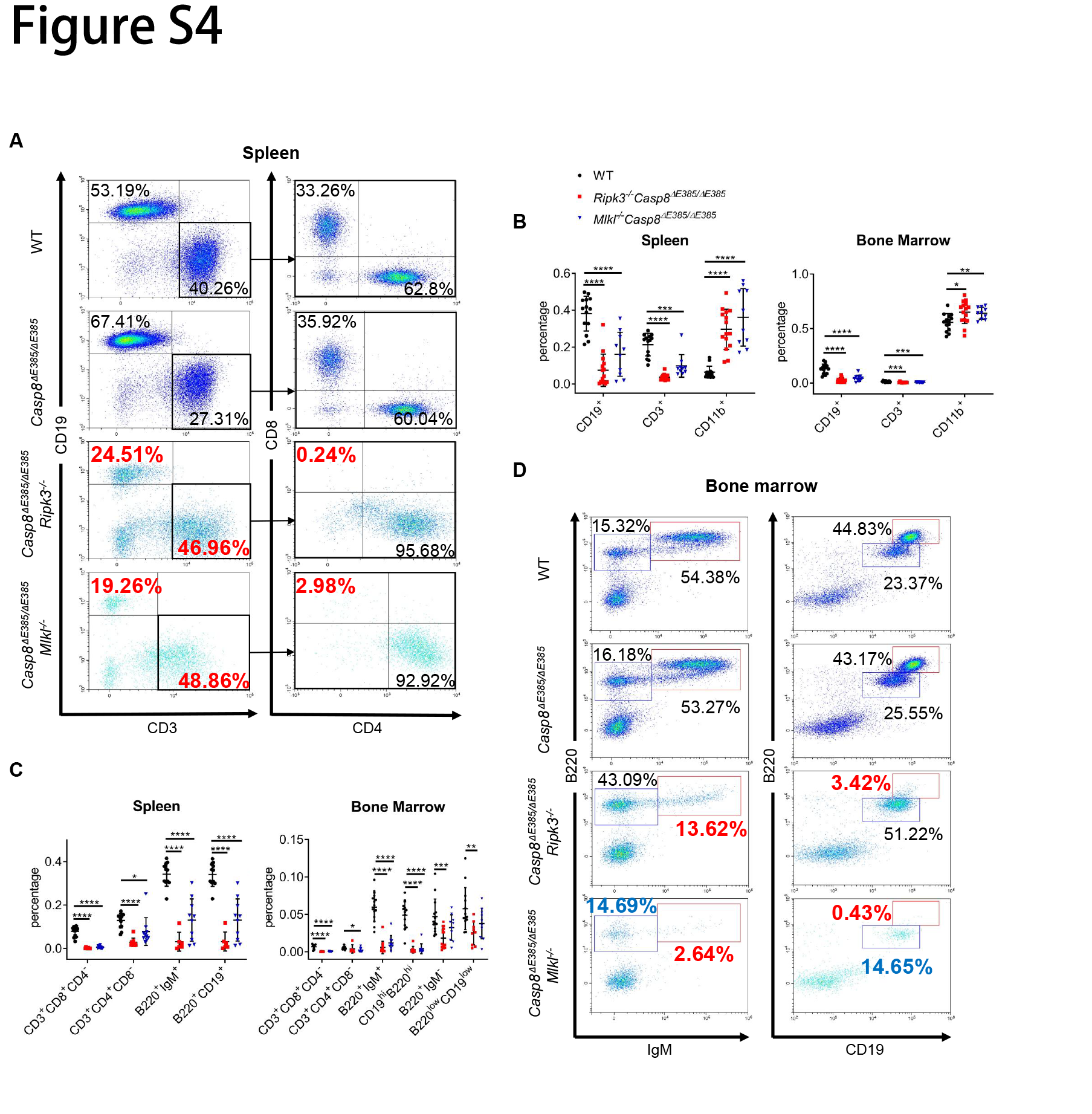

Supplement: Supplementary file 4 — Figure S4 [file 41418_2022_938_MOESM4_ESM.tif]

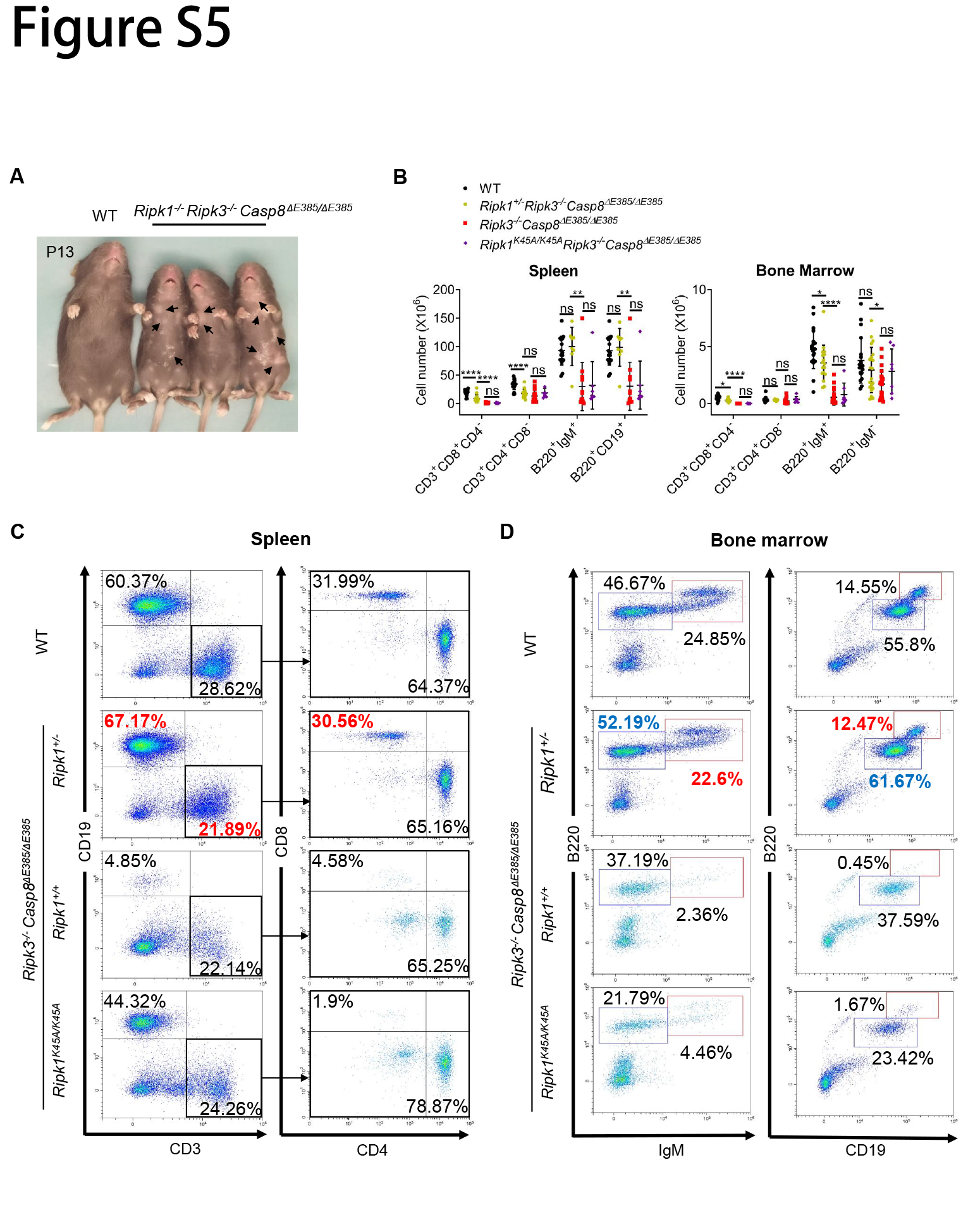

Supplement: Supplementary file 5 — Figure S5 [file 41418_2022_938_MOESM5_ESM.tif]

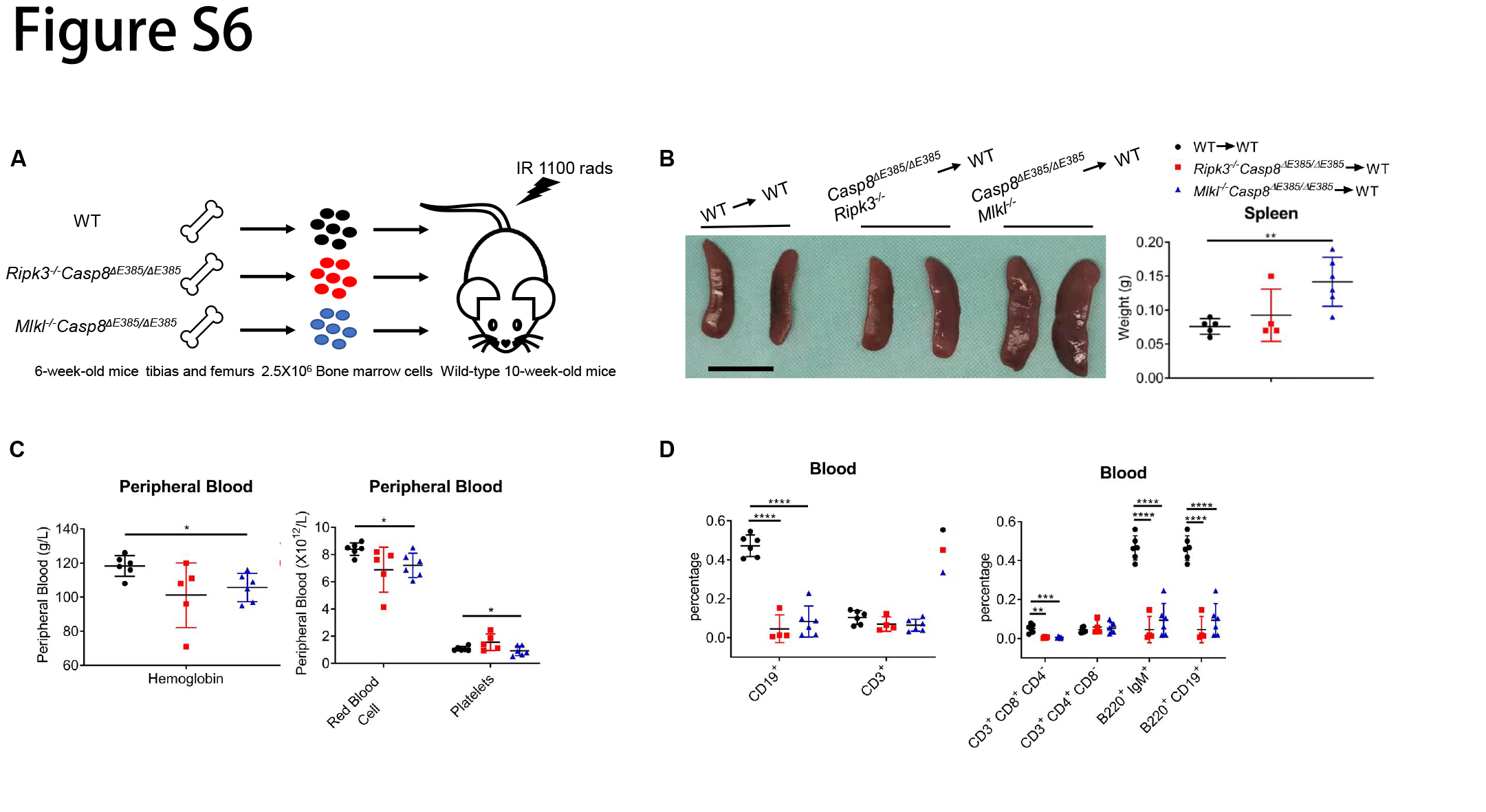

Supplement: Supplementary file 6 — Figure S6 [file 41418_2022_938_MOESM6_ESM.tif]

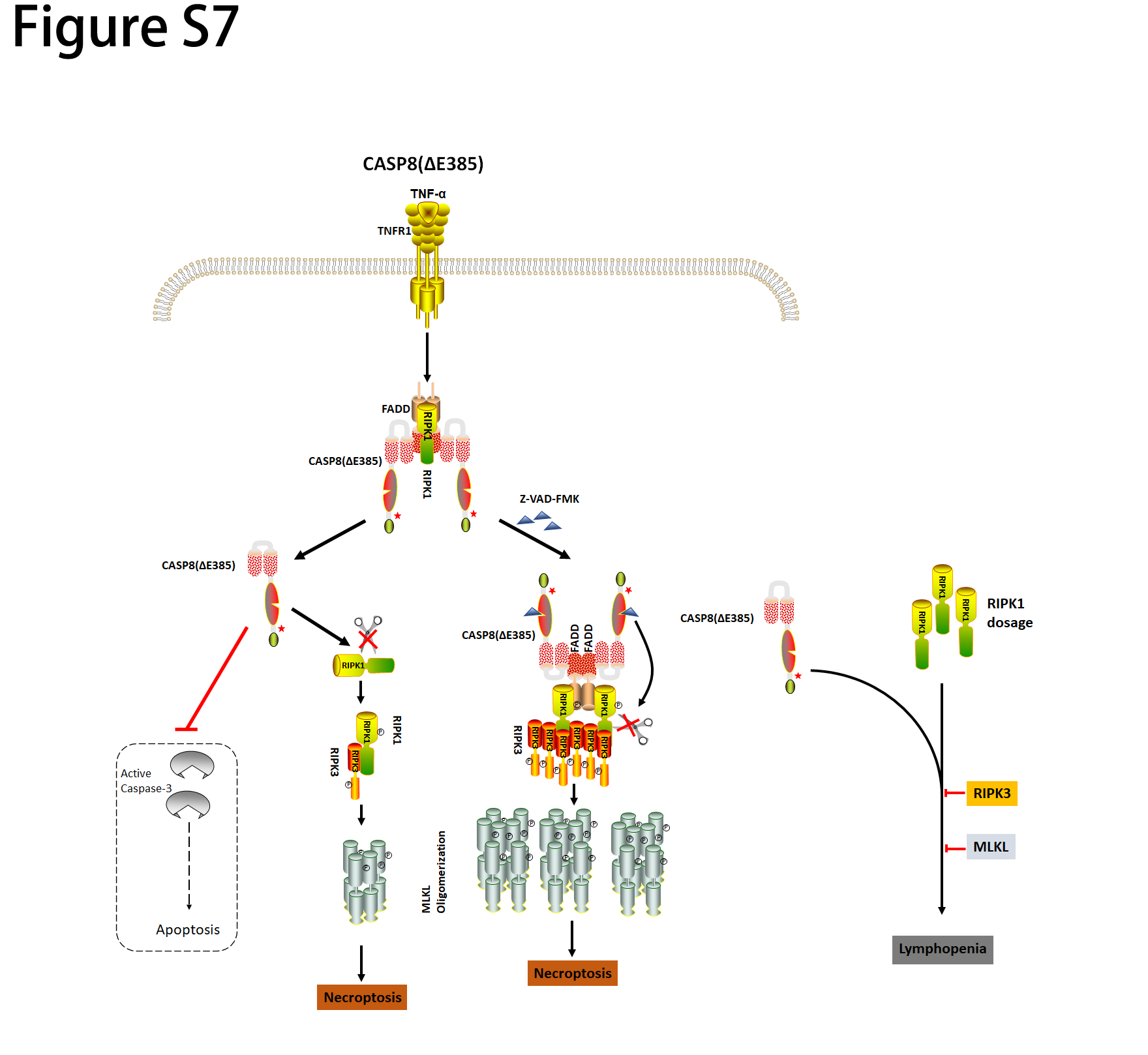

Supplement: Supplementary file 7 — Figure S7 [file 41418_2022_938_MOESM7_ESM.tif]
