# Supplementary material for: Caspase-8 auto-cleavage regulates programmed cell death and collaborates with RIPK3/MLKL to prevent lymphopenia
Source: Cell Death Differ. 2022 Jan 21;29(8):1500–12. doi: 10.1038/s41418-022-00938-9 (PMC9345959; doi:10.1038/s41418-022-00938-9)
Supplement: Supplementary file 8 — Detailed Attribution of Authorship [file 41418_2022_938_MOESM8_ESM.pdf]

DECLARATION OF CONTRIBUTIONS TO ARTICLE

ADMC

Manuscript Number:	Journal Name:	
CDD-20-2027RRRR	*Cell Death & Differentiation*	(the 'Journal')

Proposed Title of the Contribution:

Caspase-8 auto-cleavage regulates programmed cell death and collaborates with RIPK3/MLKL to prevent lymphopenia (the 'Contribution)

Author(s):

Xiaoming Li, Fang Li, Xixi Zhang, Haiwei Zhang, Qun Zhao, Ming Li, Xiaoxia Wu, Lingxia Wang, Jianling Liu, Xuanhui Wu, Yangjing Ou, Mingyan Xing, Yue Zhang, Jiangshan Deng, Xiuzhe Wang, Yan Luo, Jinbao Li, Yuwu Zhao, Haibing Zhang (the 'Authors')

For all *CDD* articles, each person named as an author in the published version must be able to show he or she has contributed substantially to the article.

Authorship credit should be based on 1) substantial contributions to conception and design, acquisition of data, or analysis and interpretation of data; 2) drafting the article or revising it critically for important intellectual content; and 3) final approval of the version to be published. Authors should meet conditions 1, 2 and 3.

Any person who cannot be shown to have made a substantial contribution to the article cannot be listed as an author in the final version. The name of any person who is deemed to have made a minor contribution can, however, appear in the Acknowledgments section of the article.

Please complete the table below to indicate the contributions of all named authors to the manuscript.

Author Full Name:	Specification of Contribution to the Manuscript:
Xiaoming Li	performed most experiments and data analyses and wrote the paper.
Fang Li	performed mouse cross and genotyping and contributed flow cytometry analysis,biochemistry analysis and manuscript writing.
Xixi Zhang,Ming Li,Lingxia Wang,Jianling Liu,Xuanhui Wu,Yangjing Ou	performed phenotype analysis and contribute to biochemistry analysis
Xiaoxia Wu	performed mouse cross and genotyping and contributed flow cytometry analysis
Mingyan Xing,Yue Zhang,Jiangshan Deng,Xiuzhe Wang	contribute to biochemistry data and cytokine analysis
Qun Zhao,Haiwei Zhang	contributed cell death analyses in vitro and Fas-induced apoptosis in vivo
Yan luo, Jinbao Li,Yuwu Zhao	contributed pathological analyses, bone marrow transfer analysis, provided essential reagent and scientific insight.
Haibing Zhang	designed the study, performed data analyses and wrote the paper

Please complete the table below to indicate the contributions of all named authors to the figures.

Figure 1:

Xiaoming Li, Fang Li, Jianling Liu, Xuanhui Wu, Yangjing Ou, Mingyan Xing, Haibing Zhang

Figure 2:

Xiaoming Li,Fang Li,Haiwei Zhang, Qun Zhao, Haibing Zhang

Figure 3:

Xiaoming Li, Xixi Zhang, Ming Li, Xiaoxia Wu, Lingxia Wang, Jianling Liu, Xuanhui Wu, Yangjing Ou, Mingyan Xing, Haibing Zhang

Figure 4:

Xiaoming Li, Haiwei Zhang, Qun Zhao, Yue Zhang, Jiangshan Deng, Xiuzhe Wang, Haibing Zhang

Figure 5:

Xiaoming Li, Fang Li, Xuanhui Wu, Yangjing Ou, Mingyan Xing, Yan Luo, Jinbao Li, Yuwu Zhao, Haibing Zhang

Figure 6:

Xiaoming Li, Yue Zhang, Jiangshan Deng, Xiuzhe Wang, Yan Luo, Jinbao Li, Yuwu Zhao, Haibing Zhang

Signed for and on behalf of the Author(s):	Print Name:	Date:
	Haibing Zhang	1/9/2022
