# Supplementary material for: Caspase-8 auto-cleavage regulates programmed cell death and collaborates with RIPK3/MLKL to prevent lymphopenia
Source: Cell Death Differ. 2022 Jan 21;29(8):1500–12. doi: 10.1038/s41418-022-00938-9 (PMC9345959; doi:10.1038/s41418-022-00938-9)
Supplement: Supplementary file 10 — Merged Files [file 41418_2022_938_MOESM10_ESM.pdf]

Caspase-8 auto-cleavage regulates programmed cell death and collaborates with RIPK3/MLKL to prevent lymphopenia

Xiaoming Li[1,6], Fang Li[2,5,6], Xixi Zhang[1], Haiwei Zhang[1], Qun Zhao[1], Ming Li[1], Xiaoxia Wu[1], Lingxia Wang[1], Jianling Liu[1], Xuanhui Wu[1], Yangjing Ou[1], Mingyan Xing[1], Yue Zhang[3], Jiangshan Deng[4], Xiuzhe Wang[4], Yan Luo[3], Jinbao Li[2], Yuwu Zhao[4], Haibing Zhang[1*]

[1] CAS Key Laboratory of Nutrition, Metabolism and Food Safety, Shanghai Institute of Nutrition and Health, University of Chinese Academy of Sciences, Chinese Academy of Sciences, Shanghai, China

[2] Department of Anesthesiology, Shanghai General Hospital, Shanghai Jiao tong University School of Medicine, Shanghai, China.

[3] Department of Anesthesiology, Ruijin Hospital, Shanghai Jiao Tong University School of Medicine, Shanghai, China.

[4] Department of Neurology, Shanghai Jiao Tong University Affiliated Sixth People's Hospital, Shanghai, China.

[5] The Second Affiliated Hospital, School of Medicine, Southern University of Science and Technology, Shenzhen, China.

[6] These authors contributed equally to this work

***Correspondence: hbzhang@sibs.ac.cn**

Abstract

Caspase-8 is an initiator of death receptor-induced apoptosis and an inhibitor of RIPK3-MLKL-dependent necroptosis. In addition, caspase-8 has been implicated in diseases such as lymphoproliferation, immunodeficiency, and autoimmunity in humans. Although auto-cleavage is indispensable for caspase-8 activation, its physiological functions remain poorly understood. Here, we generated a caspase-8 mutant lacking E385 in auto-cleavage site knock-in mouse (*Casp8*$^{\Delta E385/\Delta E385}$). *Casp8*$^{\Delta E385/\Delta E385}$ cells were expectedly resistant to Fas-induced apoptosis, however, *Casp8*$^{\Delta E385/\Delta E385}$ cells could switch TNF-α-induced apoptosis to necroptosis by attenuating RIPK1 cleavage. More importantly, CASP8(ΔE385) sensitized cells to RIPK3-MLKL-dependent necroptosis through promoting complex II formation and RIPK1-RIPK3 activation. Notably, *Casp8*$^{\Delta E385/\Delta E385}$*Ripk3*$^{-/-}$ mice partially rescued the perinatal death of *Ripk1*$^{-/-}$ mice by blocking apoptosis and necroptosis. In contrast to the *Casp8*$^{-/-}$*Ripk3*$^{-/-}$ and *Casp8*$^{-/-}$*Mlkl*$^{-/-}$ mice appearing autoimmune lymphoproliferative syndrome (ALPS), both *Casp8*$^{\Delta E385/\Delta E385}$*Ripk3*$^{-/-}$ and *Casp8*$^{\Delta E385/\Delta E385}$*Mlkl*$^{-/-}$ mice developed transplantable lymphopenia that could be significantly reversed by RIPK1 heterozygosity, but not by RIPK1 kinase dead mutation. Collectively, these results demonstrate previously unappreciated roles for caspase-8 auto-cleavage in regulating necroptosis and maintaining lymphocytes homeostasis.

Keywords:

Caspase-8, auto-cleavage, RIPK1, RIPK3, MLKL, apoptosis, necroptosis, lymphopenia, lymphocytes homeostasis

INTRODUCTION

Caspase-8 is a cysteinyl aspartate-specific protease that critically mediates extrinsic apoptosis[1, 2, 3, 4, 5] but also inhibits necroptosis[6, 7, 8, 9, 10, 11]. In addition, caspase-8 is known to be crucially involved in the inflammatory response by acting as a scaffolding protein[12, 13, 14, 15, 16]. Previous studies demonstrated the death of *Casp8*$^{-/-}$ mice from RIPK3-MLKL mediated necroptosis[9, 10]. This result highlights the critical role of the catalytic activity of caspase-8-cFLIP complex in necroptosis inhibition[9]. In accordance with that, the conditional ablation of caspase-8 in the intestinal epithelial cells or keratinocytes also leads to the inflammation and aberrant cell death in the intestine and skin, respectively[17, 18, 19, 20, 21]. This can be prevented by the co-deletion of *Ripk3*[17, 18, 19, 20, 21], indicating that caspase-8 is required for tissue homeostasis by suppressing necroptosis. Moreover, mice expressing catalytically inactive RIPK3 D161N exhibit caspase-8-dependent embryonic lethality[22], suggesting that caspase-8 mediated apoptosis plays an essential role during embryonic development. In addition, caspase-8 phosphorylation mimic T265E knock-in mice were lately reported to be embryonically lethal[23], which indicated the phosphorylation of caspase-8 impaired the blockade of necroptosis during embryo development. Furthermore, caspase-8-mediated apoptosis in association with caspase-11 and gasdermin-D-mediated epithelial cell death to regulate gut homeostasis and inflammation[20, 24]. Recent studies have demonstrated that deficiency of the enzymatic activity of CASP8(C362S)/CASP8(C362A) not only promotes necroptosis but also triggers pyroptosis when necroptosis is inhibited *in vivo*[6, 8]. The expression of catalytically inactive caspase-8 leads to embryonic lethality in mice that can be prevented by deletion of *Ripk3* or co-ablation of *Mlkl* and *Casp1*[6, 8], suggesting that the enzymatic activity of caspase-8 plays a critical role in the regulation of pyroptosis when apoptosis and necroptosis are compromised. In addition to the regulation of cell death, caspase-8 contributes to the maintenance of immune homeostasis[11, 25, 26, 27]. When embryonic lethality in *Casp8*-deficient mice is rescued by *Ripk3* or *Mlkl* ablation, the *Casp8*$^{-/-}$*Ripk3*$^{-/-}$ and *Casp8*$^{-/-}$*Mlkl*$^{-/-}$ mice develop lymphadenopathy[11] that resembles the abnormality observed in Fas ligand (FasL, CD95L)[28] or FAS[29, 30] deficient mice and

human autoimmune lymphoproliferative syndrome (ALPS)[31,32]. *Casp8*$^{C362A/C362A}$*Ripk3*$^{-/-}$ mice also develop splenomegaly[8], indicating the potential of catalytic activity of caspase-8 in immune homeostasis. Besides, caspase-8 mutation in humans causes immunodeficiency[26] in addition to ALPS, which can be explained by the mechanisms that caspase-8 cleaves and inactivates a cytokine production suppressor NEDD4-binding protein 1 (N4BP1)[33]. However, caspase-8 mutations in humans have also been linked to inflammatory bowel disease (IBD)[34] and multi-organ lymphocytic infiltration with granulomas[27], and the precise mechanisms underlying this relationship remain elusive.

On ligating with the death receptor, auto-cleavage leads to the activation of caspase-8[35,36,37,38,39,40]. This initiates apoptosis, and in turn, inhibits necroptosis by cleaving critical necroptotic mediators such as CYLD[41], c-FLIP[42], RIPK1[7,43] and RIPK3[44]. Furthermore, complete caspase-8 activation requires dimerization and auto-cleavage of procaspase-8 to unlock the enzymatic activity[35,36,37,45]. The mice harboring mutation of caspase-8 auto-cleavage site at D387 developed normally and was impaired in extrinsic apoptosis *in vivo*[7,13,46,47], and recent study showed that the non-cleavable caspase-8 caused inflammation and induced ASC oligomerization in the lack of FADD[13]. However, the role of auto-cleavage of caspase-8 in regulating necroptosis and cell death-independent function remains undefined.

Here, we generated knock-in mouse bearing caspase-8 mutation lacking E385 in the auto-cleavage site (*Casp8*$^{\Delta E385/\Delta E385}$) and found that caspase-8 mutation CASP8(ΔE385) not only switches TNF-α induced apoptosis to necroptosis by suppressing RIPK1 cleavage, but also unexpectedly promoted necroptosis through promoting complex II and RIPK1-RIPK3 activation. In addition, *Casp8*$^{\Delta E385/\Delta E385}$*Ripk3*$^{-/-}$ and *Casp8*$^{\Delta E385/\Delta E385}$*Mlkl*$^{-/-}$ mice developed lymphopenia with severe splenomegaly instead of the lymphoproliferative disease as observed in *Casp8*$^{-/-}$*Ripk3*$^{-/-}$ and *Casp8*$^{-/-}$*Mlkl*$^{-/-}$ mice. Collectively, these results suggest that caspase-8 auto-cleavage is not only required to mediate apoptosis but also inhibit necroptosis by negatively regulating

complex II formation and stabilization and cooperates with RIPK3/MLKL maintaining lymphocytes homeostasis.

RESULTS

1. *Casp8*$^{ΔE385/ΔE385}$ mice are viable but develop a slight CD8$^+$ T cell lymphopenia in the spleen.

Previous studies have demonstrated that auto-cleavage of caspase-8 is required for mediating apoptosis but not for inhibiting necroptosis during development because the mice expressing none-cleavable Caspase-8 are viable[7, 13, 46]. As expected, we observed that caspase-8 cleavage was gradually enhanced when apoptosis was induced by tumor necrosis factor α (TNF-α) plus cycloheximide (CHX) in wild-type mouse dermal fibroblasts (MDFs) (**Figure 1A**). Notably, caspase-8 cleavage was also increased in response to necroptotic stimulation with TNF-α plus Smac mimetics (Smac) and the pan-caspase inhibitor Z-VAD-FMK (zVAD). This finding was verified by observing the increased levels of phosphorylated RIPK1, RIPK3, and MLKL necroptotic markers (**Figure 1B**). Therefore, in addition to its role in mediating apoptosis, caspase-8 cleavage is also hypothesized to regulate necroptosis.

Previous studies established transgenic mice expressing caspase-8 D387A[7, 13, 46], which cannot be cleaved between the large and small catalytic subunits. Caspase-8 has a substrate preference for the tetrapeptide (Leu/Val)-Glu-X-Asp[48], which corresponds closely to the caspase-8 auto-processing substrate sequence, L384/E385/V386/D387. We therefore hypothesized that E385 of caspase-8 would also contribute to its auto-cleavage. To explore the contribution of caspase-8 (E385) in its auto-processing *in vitro* and *in vivo*, we generated a knock-in mouse that expressed caspase-8 lacking E385 in the auto-cleavage site between the large and small catalytic subunits (**Figure S1A**). In contrast to the embryonic lethality observed in caspase-8 deficiency[49] and catalytically inactive caspase-8 mice[6, 8], *Casp8*$^{ΔE385/ΔE385}$ mice were viable and matured normally (**Figure S1B**), which was consistent with previously reported mouse lines expressing caspase-8(D387A)[7, 13, 46]. To test whether CASP8(ΔE385) is indeed unable to auto-process between the large and small catalytic subunits, we treated primary WT and *Casp8*$^{ΔE385/ΔE385}$ BMDMs with LPS/BV6 to induce apoptosis. Compared with the dramatic caspase-8 cleavage in wild-type BMDMs, caspase-8 cleavage between the

large and small catalytic subunits was confirmed to be blocked in *Casp8*$^{\Delta E385/\Delta E385}$ BMDMs utilizing two different antibodies (**Figure S1C**). Besides, it was observed that the expression of CASP8(ΔE385) in multiple tissues including spleen, lung, liver, kidney, colon, heart, ileum, and rectum was normal in *Casp8*$^{\Delta E385/\Delta E385}$ mice (**Figures 1C and S1D**), suggesting that the cleavage of caspase-8 is dispensable for its expression and stability *in vivo*. Next, we examined the effect of CASP8(ΔE385) on the pathologies. Histopathological examination demonstrated that the appearance of multiple tissues was indistinguishable in *Casp8*$^{\Delta E385/\Delta E385}$ mice in comparison with the tissue appearance in WT mice (**Figure S1E**). However, we observed that the *Casp8*$^{\Delta E385/\Delta E385}$ mice developed slight splenomegaly with a mild decrease in the percentage of the CD8$^+$ T cells in the spleen and bone marrow (**Figures 1D-1F**). However, no differences were observed between *Casp8*$^{\Delta E385/\Delta E385}$ and WT mice with respect to the B cells and the myeloid cell subsets obtained from the spleen, lymph nodes, and bone marrow (**Figure 1F**). These results show that the *Casp8*$^{\Delta E385/\Delta E385}$ mice are viable but develop a slight CD8$^+$ T cell lymphopenia with splenomegaly.

2. Apoptosis induced by TNF-α was switched to necroptosis by attenuating RIPK1 cleavage in *Casp8*$^{\Delta E385/\Delta E385}$ cells.

Previous studies have demonstrated that the auto-cleavage of caspase-8 is essential for the apoptosis induced by the anti-Fas antibody Jo2, *in vitro*[7, 13, 46] and *in vivo*[13, 46]. Consistently, we observed that the thymocyte apoptosis induced by anti-Fas from *Casp8*$^{\Delta E385/\Delta E385}$ mice was compromised compared to that from WT mice (**Figure S2A**), and anti-Fas antibody also induced less caspase-3 cleavage in *Casp8*$^{\Delta E385/\Delta E385}$ thymocytes (**Figure 2A**). To further investigate the role of caspase-8 cleavage in apoptosis, we treated *Casp8*$^{\Delta E385/\Delta E385}$ MDFs with a RIPK3 kinase inhibitor, GSK'872, to induce apoptosis[50]. We observed that *Casp8*$^{\Delta E385/\Delta E385}$ MDFs were strongly resistant to apoptosis induced by GSK'872 (**Figure 2B**). This finding was confirmed by attenuating the cleavage of caspase-3 in *Casp8*$^{\Delta E385/\Delta E385}$ MDFs (**Figure 2C**). To further verify the contribution of caspase-8 cleavage in apoptosis *in vivo*, we challenged the anti-Fas antibody, Jo2, by intravenous injection in *Casp8*$^{\Delta E385/\Delta E385}$ and WT mice. In

accordance with previous studies[13, 46], *Casp8*$^{\Delta E385/\Delta E385}$ mice were significantly protected from the Jo2-induced lethal effects compared to WT mice (**Figure 2D**). Accordingly, *Casp8*$^{\Delta E385/\Delta E385}$ mice exhibited alleviated liver damage and decreased alanine aminotransferase (ALT)/aspartate aminotransferase (AST) concentrations in the plasma compared to the liver function in WT control mice (**Figures 2E and S2B**). In line with these data, we observed the absence of caspase-8 cleavage and a significant decrease in caspase-3 cleavage in the livers of *Casp8*$^{\Delta E385/\Delta E385}$ mice (**Figure S2C**), suggesting that the lethal effects exerted by the anti-Fas antibody Jo2-induced apoptosis were decreased in *Casp8*$^{\Delta E385/\Delta E385}$ mice *in vivo*. These results suggested that blocking cleavage between the large and small catalytic subunits by CASP8(ΔE385) is enough to prevent apoptosis *in vitro* and *in vivo*.

To further investigate whether CASP8(ΔE385) is required for TNF-α-induced apoptosis, we treated the *Casp8*$^{\Delta E385/\Delta E385}$ MDFs with TNF-α plus Smac. In contrast to the WT MDFs showing increased caspase-8 cleavage, *Casp8*$^{\Delta E385/\Delta E385}$ MDFs showed no detectable caspase-8 auto-cleavage between the large and small catalytic subunits (**Figure S3A**). However, in contrast with the previous findings that the apoptosis induced by GSK’872 decreased in *Casp8*$^{\Delta E385/\Delta E385}$ MDFs, we observed that increased cell death in *Casp8*$^{\Delta E385/\Delta E385}$ MDFs upon stimulation with TNF-α plus Smac/CHX compared to the death in WT MDFs (**Figure 2F**). Interestingly, we further observed that caspase-3 cleavage induced by TNF-α plus Smac in WT MDFs was decreased in *Casp8*$^{\Delta E385/\Delta E385}$ MDFs (**Figure S3A**). Given that the necroptosis suppression function of caspase-8[51], we speculated that CASP8(ΔE385) could switch apoptosis to necroptosis under certain conditions. Therefore, we measured the markers of cell death pathways in MDFs in response to the stimulation by TNF-α/CHX and TNF-α/Smac. The *Casp8*$^{\Delta E385/\Delta E385}$ MDFs showed upregulation in RIPK1, RIPK3, and MLKL phosphorylation but a decrease in RIPK1 and caspase-3 cleavage (**Figure 2G and S3A**). This indicates that the CASP8(ΔE385) switched TNF-α/CHX and TNF-α/Smac induced caspase-3-dependent apoptosis to RIPK1-RIPK3-MLKL-mediated necroptosis owing to the attenuation of RIPK1 cleavage. Collectively, these results demonstrate that caspase-8 cleavage between the large and small catalytic subunits is required for

mediating apoptosis, but CASP8(ΔE385) promotes cell death switch from apoptosis to RIPK1-RIPK3-MLKL-dependent necroptosis under certain conditions.

3. CASP8(ΔE385) promotes necroptosis upon various necroptotic stimuli both *in vitro* and *in vivo*.

Caspase-8 suppresses RIPK3-MLKL mediated necroptosis[9, 10, 11], and caspase-8 catalytic activity is essential for inhibiting necroptosis during development, as demonstrated recently[6, 8]. To investigate the role of caspase-8 auto-cleavage in necroptosis regulation, we induced necroptosis in MDFs via TNF-α plus Smac and zVAD and in bone marrow-derived macrophages (BMDMs) via stimulation with LPS or poly(I:C) plus zVAD. Notably, we observed that $Casp8^{\Delta E385/\Delta E385}$ MDFs and BMDMs showed excessive cell death compared to their WT counterparts, which could also be rescued by Nec-1 (**Figures 3A and 3B**). RIPK1[7], RIPK3[52, 53, 54] and MLKL[55, 56] are the main executors of programmed necroptosis via cascade phosphorylation. To further investigate the mechanism by which caspase-8 cleavage regulates necroptosis, we firstly examined RIPK1-RIPK3-MLKL axis signaling. Indeed, compared with the WT MDFs, the $Casp8^{\Delta E385/\Delta E385}$ MDFs showed significant increase in the phosphorylation of RIPK1, RIPK3, and MLKL and oligomerization of MLKL after TNF-α plus Smac/CHX and zVAD stimulation (**Figures 3C, 3D, S3B**). Similar results were observed in $Casp8^{\Delta E385/\Delta E385}$ BMDMs in LPS plus zVAD-induced necroptosis (**Figure S3C**).

As the pan-caspase inhibitor Z-VAD-FMK blocked the caspase-8 enzymatic activity both in wild-type and $Casp8^{\Delta E385/\Delta E385}$ cells, we wondered why $Casp8^{\Delta E385/\Delta E385}$ cells still showed excessive necroptosis compared with WT cells in the presence of zVAD. Previous study demonstrated that TNF-α induced cell death depends on complex II, which contains RIPK1, FADD, caspase-8, RIPK3 and MLKL[52, 53, 54, 57, 58, 59, 60, 61]. Regulated by several pro- and anti-apoptotic and pro- and anti-necroptotic proteins[6, 7, 8, 41, 43, 44, 62, 63, 64], complex-II can trigger apoptosis or necroptosis. Thus, we analyzed whether complex II was enhanced in $Casp8^{\Delta E385/\Delta E385}$ MDFs under necroptotic stimulation. When MDFs were stimulated by TNF-α/CHX/zVAD, we found obviously

sustained and enhanced interaction of RIPK3 and FADD with RIPK1 in *Casp8*$^{\Delta E385/\Delta E385}$ MDFs than in wild-type cells (**Figure 3E**). Furthermore, we detected dramatically increased p-Ser166 RIPK1 and p-Ser232 RIPK3 within complex II in TNFα/CHX/zVAD treated *Casp8*$^{\Delta E385/\Delta E385}$ MDFs compared to WT MDFs (**Figure 3E**). Besides, we also found enhanced complex II assembly in *Casp8*$^{\Delta E385/\Delta E385}$ BMDMs under LPS/zVAD treatment (**Figure 3F**).

As zVAD itself can promote complex II formation, we wondered whether *Casp8*$^{\Delta E385/\Delta E385}$ cells still showed enhanced complex II assembly in the absence of zVAD. Thus, we stimulated wild-type and *Casp8*$^{\Delta E385/\Delta E385}$ MDFs with TNF-α/CHX. We found that TNF-α/CHX treatment can still induce increased interaction of caspase-8, FADD and RIPK3 with RIPK1 in *Casp8*$^{\Delta E385/\Delta E385}$ cells instead of the WT cells (**Figure 3G**), suggesting that caspase-8 cleavage can negatively regulated complex II assembly. Besides, we also found increased phosphorylation of RIPK1, RIPK3 and decreased caspase-3 cleavage in TNF-α/CHX treated *Casp8*$^{\Delta E385/\Delta E385}$ MDFs, which also indicated that CASP8(ΔE385) switched TNF-α/CHX induced apoptosis to necroptosis (**Figure 3G**). Prior evidence showed that stimulation of the Toll-like receptor (TLR) and an IAP inhibitor, can also trigger complex II assembly[15, 65, 66]. Thus, we treated BMDMs with TLR4 agonist LPS plus BV6 to induce complex II formation. Consistently, LPS/BV6 treatment also induced markedly enhanced complex II formation in *Casp8*$^{\Delta E385/\Delta E385}$ BMDMs compared with WT counterpart (**Figure S3D**). Collectively, these results demonstrate that CASP8(ΔE385) functions as a scaffold to promote complex II formation in order that the recruitment of RIPK3-caspase-8-FADD and RIPK1-RIPK3 cascade phosphorylation were significantly increased and prolonged, which results in excess necroptosis in *Casp8*$^{\Delta E385/\Delta E385}$ cells. In addition, TNF-α-induced lethal systemic inflammatory syndrome has been wildly recognized as a mouse model to confirm necroptosis *in vivo*[7, 23]. Therefore, we tested whether CASP8(ΔE385) affects the lethal SIRS model in *Casp8*$^{\Delta E385/\Delta E385}$ mice. In comparison to WT, *Casp8*$^{\Delta E385/\Delta E385}$ mice showed significantly sensitized death accompanied by severe hypothermia (**Figures 4A and 4B**). Furthermore, *Ripk3*$^{-/-}$*Casp8*$^{\Delta E385/\Delta E385}$ and *Ripk1*$^{K45A/K45A}$*Casp8*$^{\Delta E385/\Delta E385}$ mice were protected to a large extent from the lethal

shock (**Figures 4A and 4B**). Moreover, to examine whether caspase-8 cleavage suppresses necroptosis in macrophages *in vivo*, WT, *Casp8*$^{\Delta E385/\Delta E385}$ and *Ripk1*$^{+/-}$*Ripk3*$^{-/-}$*Casp8*$^{\Delta E385/\Delta E385}$ mice were pretreated with zVAD followed by challenging with LPS administration. After 24 h, the CD11b$^+$F4/80$^+$ intraperitoneal macrophages (PMs) were detected by flow cytometry. The CD11b$^+$F4/80$^+$ PMs harvested from *Casp8*$^{\Delta E385/\Delta E385}$ mice were dramatically decreased compared to those observed in WT mice after the LPS plus zVAD treatment, and the excessive peripheral macrophages loss in *Casp8*$^{\Delta E385/\Delta E385}$ mice was largely protected in *Ripk1*$^{+/-}$*Ripk3*$^{-/-}$*Casp8*$^{\Delta E385/\Delta E385}$ mice (**Figures 4C, 4D**). Collectively, these data reveal that caspase-8 cleavage is essential for suppressing RIPK1-RIPK3-MLKL-mediated necroptotic death *in vitro* and *in vivo*.

4. *Casp8*$^{\Delta E385/\Delta E385}$*Ripk3*$^{-/-}$ mice develop serious lymphopenia and myeloid bias but prevent postnatal lethality in *Ripk1*$^{-/-}$ mice.

Casp8$^{-/-}$*Ripk3*$^{-/-}$ and *Casp8*$^{-/-}$*Mlkl*$^{-/-}$ mice[11], characterized by splenomegaly with a marked accumulation of CD3$^+$CD4$^-$CD8$^-$B220$^+$ T cells, resemble the deficiency of FAS ligand (FasL, CD95L)[28] or FAS (CD95)[29, 30] in mice or the autoimmune lymphoproliferative syndrome (ALPS) in humans[31]. To investigate the role of caspase-8 auto-cleavage in this disease, we generated *Casp8*$^{\Delta E385/\Delta E385}$*Ripk3*$^{-/-}$ and *Casp8*$^{\Delta E385/\Delta E385}$*Mlkl*$^{-/-}$ mice by crossing *Casp8*$^{\Delta E385/\Delta E385}$ mice to *Ripk3*$^{-/-}$ or *Mlkl*$^{-/-}$ background. Remarkably, *Casp8*$^{\Delta E385/\Delta E385}$*Ripk3*$^{-/-}$ and *Casp8*$^{\Delta E385/\Delta E385}$*Mlkl*$^{-/-}$ mice develop severe lymphopenia characterized by fewer lymphocytes in multiple organs (**Figures 5B, 5D, S4A, S4B**). Although *Casp8*$^{\Delta E385/\Delta E385}$ mice developed slight splenomegaly and CD8$^+$ T cell lymphopenia in the spleen, *Casp8*$^{\Delta E385/\Delta E385}$*Ripk3*$^{-/-}$ and *Casp8*$^{\Delta E385/\Delta E385}$*Mlkl*$^{-/-}$ mice developed more severe splenomegaly and showed a dramatically decreased percentage of B cells (CD19$^+$) and T cells (CD3$^+$) as well as an increased percentage of myeloid-derived cells (CD11b$^+$) in the spleen and bone marrow (**Figures 5A, S4A, and S4B**). This result indicates the possibility of lymphopenia and myeloid bias/myeloproliferative disease in these mice. Next, we counted the absolute cell number and observed a plummeted number of B cells (CD19$^+$) and T cells (CD3$^+$) in the spleen and bone marrow (**Figure 5B**). The macrophages and granulocytes

($CD11b^{+}$) were rapidly increased in the spleen but were approximately normal in the bone marrow (**Figure 5B**). These results characterized the lymphopenia and myeloid bias disease but excluded the possibility of myeloproliferative disease in these mice. Furthermore, we analyzed the subsets of B cells and T cells in the spleen and bone marrow. Consistent with the percentage results (**Figure S4C**), immature and mature B cells ($B220^{+}IgM^{+}$/$B220^{hi}CD19^{hi}$), progenitor B cells (pro-B) and precursor B cells (pre-B) ($B220^{+}IgM^{-}$/$B220^{low}CD19^{low}$), and $CD8^{+}$ T cells showed a dramatic decrease in the bone marrow of *Casp8*$^{\Delta E385/\Delta E385}$*Ripk3*$^{-/-}$ and *Casp8*$^{\Delta E385/\Delta E385}$*Mlkl*$^{-/-}$ mice (**Figures 5C and S4D**). In the spleen, the absolute cell numbers of peripheral B cells and T cells were also decreased (**Figure 5C**). Taken together, these data showed that the *Casp8*$^{\Delta E385/\Delta E385}$*Ripk3*$^{-/-}$ and *Casp8*$^{\Delta E385/\Delta E385}$*Mlkl*$^{-/-}$ mice develop severe myeloid bias and lymphopenia in the spleen and bone marrow.

To further confirm the presence of lymphopenia in these mice, we next analyzed the peripheral blood. Indeed, *Casp8*$^{\Delta E385/\Delta E385}$*Ripk3*$^{-/-}$ and *Casp8*$^{\Delta E385/\Delta E385}$*Mlkl*$^{-/-}$ mice showed a distinct decrease in white blood cell (WBC) and lymphocytes but normal numbers of monocytes and granulocytes in the blood (**Figure 5D**). Interestingly, *Casp8*$^{\Delta E385/\Delta E385}$ mice showed a minor increase in the WBC and lymphocyte counts (**Figure 5D**). Furthermore, the total levels of B cells ($CD19^{+}$), T cells ($CD3^{+}$) as well as mature B cells ($B220^{+}IgM^{+}$/$B220^{+}CD19^{+}$) and T cells subsets ($CD3^{+}CD4^{+}CD8^{-}$ /$CD3^{+}CD8^{+}CD4^{-}$) sharply decreased in the blood of *Casp8*$^{\Delta E385/\Delta E385}$*Ripk3*$^{-/-}$ and *Casp8*$^{\Delta E385/\Delta E385}$*Mlkl*$^{-/-}$ mice (**Figure 5E**). Collectively, these results demonstrate that caspase-8 cleavage associated with RIPK3 or MLKL plays a critical role in maintaining immune cell homeostasis.

In addition, *Ripk3*$^{-/-}$*Casp8*$^{-/-}$ can rescue the postnatal lethality of *Ripk1*$^{-/-}$ mice by inhibiting both apoptosis and necroptosis[67, 68]. Therefore, we examined whether CASP8(ΔE385) combined with the ablation of *Ripk3* contributed to the perinatal death of *Ripk1*$^{-/-}$ mice. We generated *Ripk1*$^{-/-}$*Ripk3*$^{-/-}$*Casp8*$^{\Delta E385/\Delta E385}$ mice by intercrossing *Ripk1*$^{+/-}$*Ripk3*$^{-/-}$*Casp8*$^{\Delta E385/\Delta E385}$ mice. *Ripk1*$^{-/-}$*Ripk3*$^{-/-}$*Casp8*$^{\Delta E385/\Delta E385}$ mice survived normally at birth; however, they were runted with apparent focal cutaneous lesions and

scaling on the skin, and eventually died around two weeks after birth (**Figures 5F and S5A**). These data suggest that caspase-8 cleavage mediated apoptosis combined with RIPK3 dependent necroptosis was partially responsible for the perinatal lethality of RIPK1 deficiency mice. This observation further confirmed that Caspase-8 cleavage is essential for apoptosis during development.

5. Halve the expression of RIPK1 rescues transplantable lymphopenia in $Casp8^{\Delta E385/\Delta E385}Ripk3^{-/-}$ mice.

Although $Ripk1^{-/-}Ripk3^{-/-}Casp8^{\Delta E385/\Delta E385}$ mice did not survive to adulthood, we found that $Ripk1^{+/-}Ripk3^{-/-}Casp8^{\Delta E385/\Delta E385}$ mice were viable beyond weaned and fertile. Furthermore, splenomegaly in $Ripk3^{-/-}Casp8^{\Delta E385/\Delta E385}$ mice was largely suppressed in $Ripk1^{+/-}Ripk3^{-/-}Casp8^{\Delta E385/\Delta E385}$ mice (**Figure 6A**). Consistently, myeloid bias and lymphopenia in the spleen and lymphopenia in the bone marrow were also significantly relieved in $Ripk1^{+/-}Ripk3^{-/-}Casp8^{\Delta E385/\Delta E385}$ mice compared to those in $Ripk3^{-/-}Casp8^{\Delta E385/\Delta E385}$ mice (**Figures 6B**). In addition, $Ripk1^{+/-}Ripk3^{-/-}Casp8^{\Delta E385/\Delta E385}$ mice exhibited normal immature and mature B cells ($B220^{+}IgM^{+}/B220^{hi}CD19^{hi}$) in the bone marrow (**Figures S5B and S5D**). Importantly, the complete blood count results showed increased WBC and lymphocyte in the peripheral blood of $Ripk1^{+/-}Ripk3^{-/-}Casp8^{\Delta E385/\Delta E385}$ mice compared to WT mice (**Figures 6C**), suggesting that lymphopenia and myeloid bias in $Ripk3^{-/-}Casp8^{\Delta E385/\Delta E385}$ mice were largely alleviated by halving RIPK1 dosage.

To further test whether RIPK1 kinase activity contributed to lymphopenia in $Ripk3^{-/-}Casp8^{\Delta E385/\Delta E385}$ mice, we generated $Ripk1^{K45A/K45A}Ripk3^{-/-}Casp8^{\Delta E385/\Delta E385}$ mice and observed that $Ripk1^{K45A/K45A}Ripk3^{-/-}Casp8^{\Delta E385/\Delta E385}$ mice showed lymphopenia and myeloid bias similar to $Ripk3^{-/-}Casp8^{\Delta E385/\Delta E385}$ mice (**Figures 6A-6C and S5B-S5D**). Collectively, these results demonstrate that RIPK1 dosage-dependent and RIPK1 kinase-independent scaffold function contributes to lymphopenia and myeloid bias in $Ripk3^{-/-}Casp8^{\Delta E385/\Delta E385}$ mice.

Next, we asked whether lymphopenia was intrinsic to $Ripk3^{-/-}Casp8^{\Delta E385/\Delta E385}$ and $Mlkl^{-}$

$^{-/-}$*Casp8*$^{ΔE385/ΔE385}$ hematopoietic stem cells (HSCs). The complete bone marrow of *Ripk3*$^{-/-}$*Casp8*$^{ΔE385/ΔE385}$ and *Mlkl*$^{-/-}$*Casp8*$^{ΔE385/ΔE385}$ mice was transplanted into lethally irradiated syngeneic WT recipients (**Figure S6A**). After hematopoiesis was reestablished, we observed that the mice receiving *Mlkl*$^{-/-}$*Casp8*$^{ΔE385/ΔE385}$ bone marrow developed splenomegaly, whereas the spleen of *Ripk3*$^{-/-}$*Casp8*$^{ΔE385/ΔE385}$ recipients showed no difference (**Figure S6B**). In the peripheral blood, the *Ripk3*$^{-/-}$*Casp8*$^{ΔE385/ΔE385}$ and *Mlkl*$^{-/-}$*Casp8*$^{ΔE385/ΔE385}$ recipients showed leucopenia and deficiency in every WBC subset (**Figure 6D**), while the red blood cells, platelets, and hemoglobin levels showed a minor decrease (**Figure S6C**). Consistently, lymphopenia was recapitulated in the *Ripk3*$^{-/-}$*Casp8*$^{ΔE385/ΔE385}$ and *Mlkl*$^{-/-}$*Casp8*$^{ΔE385/ΔE385}$ recipients characterized by $CD8^+$ T cell deficiency in blood and decreased B cells, T cells, and their subsets in the spleen, bone marrow, and blood (**Figures 6E, 6F and S6D**). Collectively, caspase-8 cleavage together with RIPK3 or MLKL suppresses the intrinsic lymphopenia of hematopoietic stem cells.

Discussion

Caspase-8 is a key regulator of apoptosis and necroptosis, as well as the inflammatory response through its dimerization and enzymatic activity[1, 5, 16]. The auto-cleavage activity of Caspase-8 has also been shown to be involved in mediating apoptosis and regulating inflammation[13].

In this study, we demonstrated that CASP8(ΔE385) not only compromised Fas-induced apoptosis and switched TNF-a induced apoptosis to necroptosis but also promoted necroptosis both *in vitro* and *in vivo*. However, in contrast to the embryonic lethality observed in caspase-8 deficient[49] or with catalytically inactive caspase-8 mice[6, 8], $Casp8^{\Delta E385/\Delta E385}$ mice survived normally, suggesting that primarily caspase-8 catalytic activity rather than caspase-8 cleavage contributes to the suppression of RIPK3-MLKL mediating necroptosis during embryo development.

In the current study, we observed that caspase-8 cleavage between the large and small subunits was increased under TNF-α/Smac/zVAD (**Figure 1B**), which is consistent with results from TNF-α plus zVAD stimulation in previous studies[69, 70]. It has also been suggested that pro-caspase-8 and activated caspase-8 have divergent substrate specificities[71, 72], and the substrate specificities of procaspase-8 change when it heterodimerizes with $cFLIP_L$ in complex II[42]. It has also been shown that Z-VAD-FMK is less efficacious at inhibiting the caspase-8 homodimer than the caspase-8/$cFLIP_L$ heterodimer[73]. Thus, one possible explanation is that zVAD promotes complex II formation, but its ability to inhibit the catalytic activity of pro-caspase-8 is not as efficacious as to inhibit the activated caspase-8, which contributes to more caspase-8 auto-processing.

Earlier studies have demonstrated that perinatal death in $Ripk1^{-/-}$ mice is prevented by co-ablation of FADD/caspase-8 dependent apoptosis and RIPK3/MLKL dependent necroptosis[67, 68]. Here, we generated $Ripk1^{-/-}Ripk3^{-/-}Casp8^{\Delta E385/\Delta E385}$ mice that died around two weeks to strongly prolong the survival of $Ripk1^{-/-}$ $Ripk3^{-/-}$ mice. During the manuscript preparation, a recent paper reported that $Fadd^{-/-}Mlkl^{-/-}Casp8^{DA/DA}$ also died around two weeks after birth due to the exacerbation of inflammation[13], suggesting that caspase-8 exhibits a FADD-independent inflammatory function that is inhibited by

caspase-8 cleavage. Therefore, whether lethal inflammation in *Ripk1*$^{-/-}$*Ripk3*$^{-/-}$*Casp8*$^{\Delta E385/\Delta E385}$ mice can be prevented by the additional ablation of caspase-1 as *Fadd*$^{-/-}$*Mlkl*$^{-/-}$*Casp8*$^{DA/DA}$ mice remain to be determined.

The role of caspase-8, RIPK3, and MLKL in non-programmed cell death has been reported to regulate lymphadenopathy[11], lymphoproliferation[25] and immunodeficiency[26, 33]. We demonstrated an unexpected role of caspase-8 auto-cleavage cooperating with RIPK3 or MLKL and RIPK1 in lymphopenia regulation. Unlike *Casp8*$^{-/-}$*Ripk3*$^{-/-}$ and *Casp8*$^{-/-}$*Mlkl*$^{-/-}$ mice, which resemble the human ALPS[11] and impair cytokine response[33], we found that *Casp8*$^{\Delta E385/\Delta E385}$*Ripk3*$^{-/-}$ and *Casp8*$^{\Delta E385/\Delta E385}$*Mlkl*$^{-/-}$ mice develop hematopoietic cell-intrinsic lymphopenia and myeloid bias (**Figures 5, 6**). We observed that the circulating mature B cells (B220^{+}IgM^{+}) and T cells in the peripheral blood and spleen of *Casp8*$^{\Delta E385/\Delta E385}$*Ripk3*$^{-/-}$ and *Casp8*$^{\Delta E385/\Delta E385}$*Mlkl*$^{-/-}$ mice were dramatically decreased. This can be explained by decreased immature and mature B cells and T cells in the bone marrow (**Figures 5B-5E**). Moreover, Lymphopenia and myeloid bias in *Ripk3*$^{-/-}$*Casp8*$^{\Delta E385/\Delta E385}$ mice were largely suppressed in *Ripk1*$^{+/-}$*Ripk3*$^{-/-}$*Casp8*$^{\Delta E385/\Delta E385}$ mice but not in *Ripk1*$^{K45A/K45A}$*Ripk3*$^{-/-}$*Casp8*$^{\Delta E385/\Delta E385}$ mice, revealing a previously unknown role of the dosage of RIPK1 instead of RIPK1 kinase activity administered to the mice in maintaining immune cell homeostasis in *Ripk3*$^{-/-}$*Casp8*$^{\Delta E385/\Delta E385}$ mice.

In this study, we identified the phenotypes of *Casp8*$^{\Delta E385/\Delta E385}$ mice which resemble those of the *Casp8*$^{DA/DA}$ mice from a recent study[13]. Moreover, we also confirmed the enzymatic activity of CASP8(ΔE385) by examining caspase-3 cleavage in thymocytes with FasL treatment[46]. We found that *Casp8*$^{\Delta E385/\Delta E385}$ thymocytes showed comparable level of caspase-3 cleavage and cell death to that in *Casp8*$^{D387A/D387A}$ thymocytes after FasL treatment (**Figure 2A and S2A**), which indicated CASP8(ΔE385) has comparable enzymatic activity as caspase-8(D387A)[46]. However, we still cannot exclude the possibility that deletion of one amino acid in caspase-8 alters other caspase-8-mediated cellular signaling, therefore, whether E385 deletion influences other functions of caspase-8, in addition to its auto-cleavage, needs to be investigated further.

In summary, caspase-8 auto-cleavage plays an important role in regulating cell death

and immune cell homeostasis, that is, mediating apoptosis, suppressing necroptosis, and protecting from lymphopenia (**Figure S7**). Although CASP8(ΔE385) is sufficient to suppress necroptosis during embryonic development, CASP8(ΔE385) can induce excessive necroptosis by switching apoptosis to necroptosis and promoting complex II assembly and stabilization. Accordingly, *Casp8*$^{\Delta E385/\Delta E385}$ mice are strongly sensitized to TNF-α induced necroptosis *in vivo*. Additionally, *Casp8*$^{\Delta E385/\Delta E385}$*Ripk3*$^{-/-}$ and *Casp8*$^{\Delta E385/\Delta E385}$*Mlkl*$^{-/-}$ mice develop severe lymphopenia that can be prevented by reducing the RIPK1 dosage by half, not by RIPK1 kinase inactive mutant. This indicates that caspase-8 cleavage cooperating RIPK3/MLKL to regulate RIPK1 scaffold-dependent but RIPK1 kinase-independent function contributes to the maintenance of immune cell homeostasis. The exact signaling pathway and mechanism require further investigation.

Materials and Methods

Mice

All mice utilized in this study were C57BL/6 background and housed in a specific pathogen-free (SPF) facility. Both male and female mice were used in this study. For all studies mice were age- and sex-matched. *Ripk1*$^{+/-}$, *Ripk3*$^{-/-}$, *Ripk1*$^{K45A/K45A}$ and *Mlkl*$^{-/-}$ mouse lines have been described previously[74, 75]. *Casp8*$^{\Delta E385/\Delta E385}$ mice were generated by CRISPR-Cas9 mutation system (Bioray Laboratories Inc., Shanghai, China). Three adjacent nucleotides AAG was removed in the exon 8 of the *Casp8* gene locus resulted in the deletion of Glutamic acid (Glu, E) in 385 position of caspase-8 protein sequence. The *Casp8* (ID: 12370) gene region corresponds to genomic position chr1: 58844689-58844691. *Casp8*$^{\Delta E385/\Delta E385}$ mice genotyping primers: 5′-CAGAGGCTCTGAGTAAGACC-3′ and 5′-CTGAGGACATCTTTCCCTCAG-3′ amplified 506bp DNA fragments for sequencing. Additional information is provided upon request. Animal experiments were conducted in accordance with the guidelines of the Institutional Animal Care and Use Committee of the Institute of Nutrition and Health, Shanghai Institutes for Biological Sciences, University of Chinese Academy of Sciences.

Isolation and culture of thymocytes, mouse dermal fibroblasts (MDFs) and bone marrow derived macrophages (BMDMs)

Both male and female mice were used to generate MDFs and BMDMs. MDFs were separated from the skin of newborn mice (P0-P1), and cultured in DMEM medium (SH30243.01B, HyClone) supplemented with 10% of Fetal Bovine Serum (04-001-1A, Bioind) and 1% of penicillin/streptomycin (15140122, Gibco). BMDMs were isolated from the bone marrow of mouse femurs and tibias followed by inducing to differentiate *in vitro*. Bone marrow cells were cultured for 7 days in RPMI-1640 medium (SH30809.01B, HyClone) containing 10% of Fetal Bovine Serum (04-001-1A, Bioind) and 1% of penicillin/streptomycin (15140122, Gibco) and 50 ng/ml M-CSF (AF-315-02, PeproTech), and medium was refreshed each 3 days. Cells were cultivated at 37°C with 5% CO_2.

Cell death stimulation and Cell survival assay

MDFs were plated in 96-well plates 12 hours before stimulation at a concentration of $1X10^4$ cells per well. For TNF-α induced apoptosis and necroptosis stimulation, MDFs were treated with TNF-α (20 ng/ml) (T) for 10 hours, TNF-α (20 ng/ml)+Smac (1 μM) (TS), TNF-α+Smac +Necrostatin-1 (30 μM) (TSN), TNF-α+Smac+zVAD (20 μM) (TSZ), TNF-α+Smac+zVAD+Nec-1 (TSZN) for 6.45 hours, TNF-α(20 ng/ml)+CHX (20 μg/ml) (TC), TNF-α+CHX+Necrostatin-1 (30 μM)(TCN), TNF-α+CHX+zVAD (20 μM) (TCZ), and TNF-α+CHX+zVAD+Nec-1 (TCZN) for 4.45 hours. For GSK'872 induced apoptosis, MDFs were treated with GSK'872 in concentration of 3 μM, 6 μM and 10 μM for 10 hours respectively.

BMDMs were plated in 96-well plates 12 hours before stimulation at a concentration of $2X10^4$ cells per well. For TNF-α, LPS and poly(I:C) induced apoptosis and necroptosis stimulation, BMDMs were treated with TNF-α (20 ng/ml)+Smac (1 μM)+zVAD (20 μM) (TSZ), TNF-α+Smac+zVAD+Nec-1 (30 μM) (TSZN), LPS (100 ng/ml) (L), LPS (100 ng/ml)+zVAD (20 μM) (LZ), LPS+zVAD+Nec-1 (30 μM) (LZN), poly(I:C) (100 μg/ml) (P), poly(I:C) (100 μg/ml)+zVAD (20 μM) (PZ), poly(I:C)+zVAD+Nec-1 (30 μM) (PZN) for 3 hours.

Thymocytes were plated in 96-well plates 12 hours before stimulation at a concentration of $4X10^4$ cells per well. For Fas-induced apoptosis, thymocytes were treated with anti-Fas antibody (Jo-2, 100ng/ml)+CHX (30 μg/ml) (FC) for 12h, 15h, 18h and 21h respectively.

Cell survival was determined using the CellTiter-Glo Luminescent Cell Viability Assay kit (G7572, Promega) and the luminescence was recorded with a microplate luminometer (5300170, Thermo Scientific).

Cell death Analysis by Western blot (WB) and complex II immunoprecipitation

MDFs were plated in 6-cm dishes 12 hours before stimulation at a concentration of $2X10^6$ cells per dish. For TNF-α induced apoptosis and necroptosis stimulation, MDFs were treated with TNF-α (40 ng/ml)+Smac (2 μM) (TS), TNF-α (20 ng/ml)+Smac (1 μM)+zVAD (20 μM) (TSZ), TNF-α (40 ng/ml)+CHX (40 μg/ml) (TC), TNF-α (40 ng/ml)+CHX (40 μg/ml) +zVAD (20 μM) (TCZ) for the indicated time. For GSK'872

induced apoptosis, MDFs were treated with GSK’872 (20 μM) for the indicated time. BMDMs were plated in 6-cm dishes 12 hours before stimulation at a concentration of 2X10^{6} cells per dish. For LPS induced necroptosis stimulation, BMDMs were treated with LPS (200 ng/ml) (L), LPS+zVAD (40 μM) (LZ) for 6 hours.

Cells were harvested after stimulation, washed with PBS and lysates with RIPA lysis buffer (50 mM Tris-HCl (pH7.4), 150 mM NaCl, 2 mM EDTA, 1% NP-40, 0.1% SDS, Protease inhibitor Cocktail (4693132001, Roche), Phosphatase inhibitor Cocktail 3 (P0044-1ML, Sigma)) for 30-45 minutes on ice. The lysates were centrifuged for 20 min at 13,200g, 4°C, quantified by BCA kit (P0010S, Beyotime) and then mixed with SDS sample buffer (250 mM Tris-Cl (PH 6.8), 10% SDS, 30% Glycerol, 5% β-mercapitalethanol, 0.02% Bromophenol blue) followed by boiling at 100°C for 10 min. The proteins were separated by SDS-PAGE, and then transferred to PVDF membrane (IPVH00010, Millipore) at 110v for 3h. Membranes were blocked with 5% skimmed milk in PBST 0.1% for 1h. Membranes were washed three times with PBST 0.1% for 7 minutes. Membranes were incubated in PBST 0.1% containing primary antibodies at 4°C overnight. The proteins were detected by chemiluminescent substrate (34080, Thermo Scientific) using Tanon 5200 Multi Luminescent Imaging Workstation (Tanon). For mouse tissue protein extraction, the indicated tissues were ground into powder by pestle and mortar with liquid nitrogen, and the protein was extracted with RIPA lysis buffer followed by centrifugation, quantification, SDS-PAGE and transmembrane as above. For GSK’872 induced apoptosis detection in **Figure 2B**, the MDFs were harvested by RIPA lysis buffer with 6M Urea.

For complex II immunoprecipitation (IP), cells were lysed with lysis buffer (20 mM Tris-HCl (pH 7.5), 1% Triton X-100, 0.2% NP-40, 120mM NaCl, 0.27M sucrose, 1mM EDTA, 1mM EGTA, 50mM NaF, 10mM β-glycerophosphate, 5mM sodium pyrophosphate, 2mM PMSF, Protease inhibitor Cocktail (4693132001, Roche), Phosphatase inhibitor Cocktail 3 (P0044-1ML, Sigma)). Cell lysates were overnight incubated with 1 μg of anti-RIPK1 (610459, BD Biosciences) at 4°C followed by 4h incubation with 50μl of Protein A agarose (16-125, Millipore). Beads were washed and proteins were eluted with 2X SDS sample buffer followed by boiling at 100°C for

10 min.

The primary antibodies used for western blot: anti-RIPK1 (610459, BD Biosciences), anti-phosphorylated RIPK1 (31122S, Cell Signaling Technology), anti-RIPK3 (2283, Prosci), anti-phosphorylated RIPK3 (ab195117, Abcam), anti-caspase-8 (ALX-804-447-C100, Enzo Life Science), anti-cleaved caspase-8 (9429S, Cell Signaling Technology), anti-caspase-8 (4927S, Cell Signaling Technology), anti-MLKL (AP14272b, Abgent), anti-phosphorylated MLKL (ab196436, Abcam), anti-FADD (ab124812, Abcam), anti-PARP (9542S, Cell Signaling Technology), anti-caspase-3 (9662S, Cell Signaling Technology), anti-cleaved caspase-3 (9661S, Cell Signaling Technology), anti-β-actin (3779, Prosci), anti-GAPDH (G9545, Sigma).

Anti-Fas induced thymocytes apoptosis analyzed by Flow cytometry and western blot

Both male and female mice were used to harvest thymocytes. Thymocytes were harvested from wild-type and *Casp8*$^{\Delta E385/\Delta E385}$ mice of 1-month old, and cultured in DMEM medium (SH30243.01B, HyClone) supplemented with 10% of heat-inactivated Fetal Bovine Serum (04-001-1A, Bioind), 1% of penicillin/streptomycin (15140122, Gibco), 200 mM L-glutamine (25030-081, Gibco), 1X MEM non-essential amino acids (NEAA) (11140-050, Gibco) and 55 mM 2-Mercaptoethanol (M6250, Sigma). Cells were cultivated at 37°C with 5% CO_2.

For flowcytometry analysis, thymocytes were plated in 6-well plates followed by stimulation at a concentration of 1X10^6 cells per well, and thymocytes were treated with 2μg/ml anti-Fas antibody (Jo-2, 554255, BD) for 24 hours followed by staining with FITC-Annexin V and PI utilizing apoptosis detection kit (C1062L, Beyotime). After staining, cells were analyzed in cytoflex S flow cytometer (cytoflex S, Beckman Coulter). All analyses were performed using CytExpert software (CytExpert, Beckman Coulter, Inc.).

For western blot analysis, thymocytes were plated in 10-cm dish followed by stimulation at a concentration of 2X10^7 cells per well, and thymocytes were treated with 1μg/ml anti-Fas antibody (Jo-2, 554255, BD) for the indicated time followed by

washing with 1XPBS and lysates with RIPA lysis buffer (50 mM Tris-HCl (pH7.4), 150 mM NaCl, 2 mM EDTA, 1% NP-40, 0.1% SDS, Protease inhibitor Cocktail (4693132001, Roche), Phosphatase inhibitor Cocktail 3 (P0044-1ML, Sigma)).

MLKL oligomerization detection

MDFs were cultured in 6-cm dishes at a concentration of 2X10^{6} cells per dish and challenged by TNF-α (20 ng/ml) +Smac (1 μM) +zVAD (20 μM) for the indicated time. MDFs were harvested at different time points and lysed with non-reducing sample buffer (125 mM Tris-Cl (PH 6.8), 20% Glycerol, 0.02% Bromophenol blue) immediately. Total cell lysates were separated using SDS-PAGE, transferred to PVDF membrane (IPVH00010, Millipore), and detected with the indicated antibodies.

Anti-Fas induced hepatocellular apoptosis and analysis of the serum and liver damage

The wild-type and $Casp8^{\Delta E385/\Delta E385}$ mice of 8- to 12-week old were injected intravenously with anti-Fas antibody (Jo-2, 554255, BD) in the dose of 0.5 μg/g and their survival time was followed for 20 hours. At the indicated times, their livers and peripheral blood were harvested followed by processing for histological analysis, western blot and analyzing the alanine transaminase (ALT) and aspartate transaminase (AST) levels in serum. To analyze the ALT and AST levels in serum, the peripheral blood of the indicated mice were collected in anticoagulation tube followed by centrifugation at 7000g, 4℃ for 30 minutes. The serum was collected to detect ALT (3040280, Shanghai Shensuo UNF Medical Diagnostic Articles Co.) and AST (3050280, Shanghai Shensuo UNF Medical Diagnostic Articles Co.) level utilizing the kit.

TNF-α induced mice toxicity and analysis of the body temperature

The WT, $Casp8^{\Delta E385/\Delta E385}$, $Casp8^{\Delta E385/\Delta E385}Ripk3^{-/-}$ and $Casp8^{\Delta E385/\Delta E385}Ripk1^{K45A/K45A}$ mice of 8- to 16-week old were injected intravenously with TNF-α (CRT192C, Cell sciences and obtained from Dr. Yi Zhang at Shanghai Institute of Nutrition and Health, CAS) in the dose of 7 μg each mouse and their body temperature was measured every 2 hours until the twelfth hour after injection.

Flow cytometry analyses

Lymphocytes were isolated from the peripheral blood, spleen, bone marrow and lymph nodes of the indicated mice. Total cell numbers were counted using counting slides (SD-100, Nexcelom) in Cellometer Mini Automated Cell Counter (Nexcelom). Surface antigens were stained with indicated conjugated primary antibodies in the staining buffer (1×PBS, 3% BSA, 1 mM EDTA, 0.1%NaN_3) at 4°C for 30 minutes. Antibodies used are asfollows: FITC anti-CD3 (11-0031-82, eBioscience), APC Cy7 anti-CD4 (552051, BD Biosciences), PerCp anti-CD8 (100732, Biolegend), PE anti-B220 (12-0452-83, eBioscience), APC anti-B220 (17-0452-83, eBioscience), APC anti-CD11b (17-0112-83, eBioscience), Brilliant Violet 421 anti-CD11b (562605, BD Biosciences), PE Cy7 anti-CD19 (25-0193-82, eBioscience), FITC anti-IgM (115-097-020, Jackson Laboratories), FITC anti-F4/80 (11-4801-85, eBioscience) were used for flow cytometry analysis in this study. After staining, cells were washed once with 1XPBS and immediately analyzed by in cytoflex S flow cytometer (cytoflex S, Beckman Coulter). All analyses were performed using CytExpert software (CytExpert, Beckman Coulter, Inc.).

Analyses of CD11b$^+$ F4/80$^+$ peritoneal macrophages *in vivo*

Wild-type, *Casp8*$^{\Delta E385/\Delta E385}$ and *Ripk1*$^{+/-}$*Ripk3*$^{-/-}$*Casp8*$^{\Delta E385/\Delta E385}$ mice were injected intraperitoneally with vehicle or zVAD (20 mg/kg) 1h before intraperitoneal injection with PBS or LPS (10 mg/kg). Animals were killed at twenty fourth hour after the first injection, resident peritoneal cells were harvested by lavage of the peritoneal cavity with 8 ml PBS. CD11b$^+$F4/80$^+$ peritoneal macrophages were analyzed by flow cytometry.

Bone marrow transplantation Assay

All of the recipient mice were wild type with C57BL/6 background, which received 11 Gy of total body irradiation in a split dose (550 rads) with 4-hour rest between doses using a Cesium-137 irradiator. Irradiated recipients were reconstituted by intravenous injection of 2.5X10^6 bone marrow cells from femurs and tibias of the 6-week old indicated genotype mice. Recipients were sacrificed at fourth months after reconstitution.

Whole blood count analysis

The whole peripheral blood of the indicated mice was collected in anticoagulation tube followed by diluting in EDTA buffer (0.5 M EDTA pH8.0) at a ratio of 1:1, and then diluted peripheral blood was analyzed on an auto hematology analyzer (BC-2800Vet, Mindray).

Quantification and Statistical Analysis

Please refer to the figure legends for description of sample size (n) and statistical significance. Data were analyzed with GraphPad Prism 8.0 software using the two-tailed unpaired Student t test or two-sided Log-rank (Mantel-Cox) test. Bars, mean$\pm$standard deviation (mean$\pm$SD). Differences were considered statistically significant when the *P* value was less than 0.05, where ****$p < 0.0001$, ***$p < 0.001$, **$p < 0.01$, *$p < 0.05$, ns, not significant.

DATA AVAILABILITY

The authors declare that all data supporting the findings of this study are present in the paper and/or the Supplementary Materials.

References

1. Kesavardhana S, Malireddi RKS, Kanneganti TD. Caspases in Cell Death, Inflammation, and Pyroptosis. *Annu Rev Immunol* 2020, **38:** 567-595.

2. Lavrik IN, Golks A, Krammer PH. Caspases: pharmacological manipulation of cell death. *J Clin Invest* 2005, **115**(10)**:** 2665-2672.

3. Salvesen GS, Walsh CM. Functions of caspase 8: the identified and the mysterious. *Semin Immunol* 2014, **26**(3)**:** 246-252.

4. Tummers B, Green DR. Caspase-8: regulating life and death. *Immunol Rev* 2017, **277**(1)**:** 76-89.

5. Van Opdenbosch N, Lamkanfi M. Caspases in Cell Death, Inflammation, and Disease. *Immunity* 2019, **50**(6)**:** 1352-1364.

6. Fritsch M, Gunther SD, Schwarzer R, Albert MC, Schorn F, Werthenbach JP*, et al.* Caspase-8 is the molecular switch for apoptosis, necroptosis and pyroptosis. *Nature* 2019, **575**(7784)**:** 683-687.

7. Newton K, Wickliffe KE, Dugger DL, Maltzman A, Roose-Girma M, Dohse M*, et al.* Cleavage of RIPK1 by caspase-8 is crucial for limiting apoptosis and necroptosis. *Nature* 2019, **574**(7778)**:** 428-431.

8. Newton K, Wickliffe KE, Maltzman A, Dugger DL, Reja R, Zhang Y*, et al.* Activity of caspase-8 determines plasticity between cell death pathways. *Nature* 2019, **575**(7784)**:** 679-682.

9. Kaiser WJ, Upton JW, Long AB, Livingston-Rosanoff D, Daley-Bauer LP, Hakem R*, et al.* RIP3 mediates the embryonic lethality of caspase-8-deficient mice. *Nature* 2011, **471**(7338)**:** 368-372.

10. Oberst A, Dillon CP, Weinlich R, McCormick LL, Fitzgerald P, Pop C*, et al.* Catalytic activity of the caspase-8-FLIP(L) complex inhibits RIPK3-dependent necrosis. *Nature* 2011, **471**(7338)**:** 363-367.

11. Alvarez-Diaz S, Dillon CP, Lalaoui N, Tanzer MC, Rodriguez DA, Lin A*, et al.* The Pseudokinase MLKL and the Kinase RIPK3 Have Distinct Roles in Autoimmune Disease Caused by Loss of Death-Receptor-Induced Apoptosis. *Immunity* 2016, **45**(3)**:** 513-526.

12. Kang S, Fernandes-Alnemri T, Rogers C, Mayes L, Wang Y, Dillon C*, et al.* Caspase-8 scaffolding function and MLKL regulate NLRP3 inflammasome activation downstream of TLR3. *Nat Commun* 2015, **6:** 7515.

13. Tummers B, Mari L, Guy CS, Heckmann BL, Rodriguez DA, Ruhl S, *et al.* Caspase-8-Dependent Inflammatory Responses Are Controlled by Its Adaptor, FADD, and Necroptosis. *Immunity* 2020.

14. Henry CM, Martin SJ. Caspase-8 Acts in a Non-enzymatic Role as a Scaffold for Assembly of a Pro-inflammatory "FADDosome" Complex upon TRAIL Stimulation. *Mol Cell* 2017, **65**(4)**:** 715-729 e715.

15. Kang TB, Yang SH, Toth B, Kovalenko A, Wallach D. Caspase-8 blocks kinase RIPK3-mediated activation of the NLRP3 inflammasome. *Immunity* 2013, **38**(1)**:** 27-40.

16. Monie TP, Bryant CE. Caspase-8 functions as a key mediator of inflammation and pro-IL-1beta processing via both canonical and non-canonical pathways. *Immunol Rev* 2015, **265**(1)**:** 181-193.

17. Gunther C, Buchen B, He GW, Hornef M, Torow N, Neumann H, *et al.* Caspase-8 controls the gut response to microbial challenges by Tnf-alpha-dependent and independent pathways. *Gut* 2015, **64**(4)**:** 601-610.

18. Gunther C, Martini E, Wittkopf N, Amann K, Weigmann B, Neumann H, *et al.* Caspase-8 regulates TNF-alpha-induced epithelial necroptosis and terminal ileitis. *Nature* 2011, **477**(7364)**:** 335-339.

19. Weinlich R, Oberst A, Dillon CP, Janke LJ, Milasta S, Lukens JR, *et al.* Protective roles for caspase-8 and cFLIP in adult homeostasis. *Cell Rep* 2013, **5**(2)**:** 340-348.

20. Schwarzer R, Jiao H, Wachsmuth L, Tresch A, Pasparakis M. FADD and Caspase-8 Regulate Gut Homeostasis and Inflammation by Controlling MLKL- and GSDMD-Mediated Death of Intestinal Epithelial Cells. *Immunity* 2020.

21. Kovalenko A, Kim JC, Kang TB, Rajput A, Bogdanov K, Dittrich-Breiholz O, *et al.* Caspase-8 deficiency in epidermal keratinocytes triggers an inflammatory skin disease. *J Exp Med* 2009, **206**(10)**:** 2161-2177.

22. Newton K, Dugger DL, Wickliffe KE, Kapoor N, de Almagro MC, Vucic D, *et al.* Activity of protein kinase RIPK3 determines whether cells die by necroptosis or apoptosis. *Science* 2014, **343**(6177)**:** 1357-1360.

23. Yang ZH, Wu XN, He P, Wang X, Wu J, Ai T, *et al.* A Non-canonical PDK1-RSK Signal Diminishes Pro-caspase-8-Mediated Necroptosis Blockade. *Mol Cell* 2020.

24. Mandal P, Feng Y, Lyons JD, Berger SB, Otani S, DeLaney A, *et al.* Caspase-8 Collaborates with Caspase-11 to Drive Tissue Damage and Execution of Endotoxic Shock. *Immunity* 2018, **49**(1)**:** 42-55 e46.

25. Kanderova V, Grombirikova H, Zentsova I, Reblova K, Klocperk A, Fejtkova M, *et al.* Lymphoproliferation, immunodeficiency and early-onset inflammatory bowel disease associated with a novel mutation in Caspase 8. *Haematologica* 2019, **104**(1)**:** e32-e34.

26. Chun. HJ, Zheng. L, Ahmad. M, Wang. J, Speirs. CK, Siegel. RM, *et al.* Pleiotropic defects in lymphocyte activation caused by caspase-8 mutations lead to human immunodeficiency. *Nature* 2002, **419:** 395-399.

27. Niemela J, Kuehn HS, Kelly C, Zhang M, Davies J, Melendez J, *et al.* Caspase-8 Deficiency Presenting as Late-Onset Multi-Organ Lymphocytic Infiltration with Granulomas in two Adult Siblings. *J Clin Immunol* 2015, **35**(4)**:** 348-355.

28. LA OR, Tai L, Lee L, Kruse EA, Grabow S, Fairlie WD, *et al.* Membrane-bound Fas ligand only is essential for Fas-induced apoptosis. *Nature* 2009, **461**(7264)**:** 659-663.

29. Watanabe-Fukunaga R, Brannan CI, Copeland NG, Jenkins NA, Nagata S. Lymphoproliferation disorder in mice explained by defects in Fas antigen that mediates apoptosis. *Nature* 1992, **356**(6367)**:** 314-317.

30. Watanabe-Fukunaga R, Brannan CI, Copeland NG, Jenkins NA, Nagata S. Pillars article: lymphoproliferation disorder in mice explained by defects in Fas antigen that mediates apoptosis. 1992. *J Immunol* 2012, **189**(11)**:** 5101-5104.

31. Shah S, Wu E, Rao VK, Tarrant TK. Autoimmune lymphoproliferative syndrome: an update and review of the literature. *Curr Allergy Asthma Rep* 2014, **14**(9)**:** 462.

32. Rieux-Laucat F, Le Deist F, Hivroz C, Roberts IA, Debatin KM, Fischer A, *et al.* Mutations in Fas associated with human lymphoproliferative syndrome and autoimmunity. *Science* 1995, **268**(5215)**:** 1347-1349.

33. Gitlin AD, Heger K, Schubert AF, Reja R, Yan D, Pham VC, *et al.* Integration of innate immune signaling by caspase-8 cleavage of N4BP1. *Nature* 2020.

34. Lehle AS, Farin HF, Marquardt B, Michels BE, Magg T, Li Y, *et al.* Intestinal Inflammation and Dysregulated Immunity in Patients With Inherited Caspase-8 Deficiency. *Gastroenterology* 2019, **156**(1)**:** 275-278.

35. Boatright KM, Renatus M, Scott FL, Sperandio S, Shin H, Pedersen IM, *et al.* A unified model for apical caspase activation. *Mol Cell* 2003, **11**(2)**:** 529-541.

36. Donepudi M, Mac Sweeney A, Briand C, Grutter MG. Insights into the regulatory mechanism for caspase-8 activation. *Mol Cell* 2003, **11**(2)**:** 543-549.

37. Oberst A, Pop C, Tremblay AG, Blais V, Denault JB, Salvesen GS*, et al.* Inducible dimerization and inducible cleavage reveal a requirement for both processes in caspase-8 activation. *J Biol Chem* 2010, **285**(22)**:** 16632-16642.

38. Muzio M, Stockwell BR, Stennicke HR, Salvesen GS, Dixit VM. An induced proximity model for caspase-8 activation. *J Biol Chem* 1998, **273**(5)**:** 2926-2930.

39. Salvesen GS, Dixit VM. Caspase activation: the induced-proximity model. *Proc Natl Acad Sci U S A* 1999, **96**(20)**:** 10964-10967.

40. Pop C, Fitzgerald P, Green DR, Salvesen GS. Role of proteolysis in caspase-8 activation and stabilization. *Biochemistry* 2007, **46**(14)**:** 4398-4407.

41. O'Donnell MA, Perez-Jimenez E, Oberst A, Ng A, Massoumi R, Xavier R*, et al.* Caspase 8 inhibits programmed necrosis by processing CYLD. *Nat Cell Biol* 2011, **13**(12)**:** 1437-1442.

42. Pop C, Oberst A, Drag M, Van Raam BJ, Riedl SJ, Green DR*, et al.* FLIP(L) induces caspase 8 activity in the absence of interdomain caspase 8 cleavage and alters substrate specificity. *Biochem J* 2011, **433**(3)**:** 447-457.

43. Lin Y, Devin A, Rodriguez Y, Liu ZG. Cleavage of the death domain kinase RIP by caspase-8 prompts TNF-induced apoptosis. *Genes Dev* 1999, **13**(19)**:** 2514-2526.

44. Feng S, Yang Y, Mei Y, Ma L, Zhu DE, Hoti N*, et al.* Cleavage of RIP3 inactivates its caspase-independent apoptosis pathway by removal of kinase domain. *Cell Signal* 2007, **19**(10)**:** 2056-2067.

45. Pop C, Timmer J, Sperandio S, Salvesen GS. The apoptosome activates caspase-9 by dimerization. *Mol Cell* 2006, **22**(2)**:** 269-275.

46. Kang TB, Oh GS, Scandella E, Bolinger B, Ludewig B, Kovalenko A*, et al.* Mutation of a self-processing site in caspase-8 compromises its apoptotic but not its nonapoptotic functions in bacterial artificial chromosome-transgenic mice. *J Immunol* 2008, **181**(4)**:** 2522-2532.

47. Yao Z, Duan S, Hou D, Heese K, Wu M. Death effector domain DEDa, a self-cleaved product of caspase-8/Mch5, translocates to the nucleus by binding to ERK1/2 and upregulates procaspase-8 expression via a p53-dependent mechanism. *EMBO J* 2007, **26**(4)**:** 1068-1080.

48. Thornberry NA, Rano TA, Peterson EP, Rasper DM, Timkey T, Garcia-Calvo M*, et al.* A combinatorial approach defines specificities of members of the caspase family and granzyme B. Functional relationships established for key mediators of apoptosis. *J Biol Chem* 1997, **272**(29)**:** 17907-17911.

49. Varfolomeev EE, Schuchmann M, Luria V, Chiannilkulchai N, Beckmann JS, Mett IL*, et al.* Targeted disruption of the mouse Caspase 8 gene ablates cell death induction by the TNF receptors, Fas/Apo1, and DR3 and is lethal prenatally. *Immunity* 1998, **9**(2)**:** 267-276.

50. Mandal P, Berger SB, Pillay S, Moriwaki K, Huang C, Guo H*, et al.* RIP3 induces apoptosis independent of pronecrotic kinase activity. *Mol Cell* 2014, **56**(4)**:** 481-495.

51. Orning P, Lien E. Multiple roles of caspase-8 in cell death, inflammation, and innate immunity. *J Leukoc Biol* 2020.

52. He S, Wang L, Miao L, Wang T, Du F, Zhao L*, et al.* Receptor interacting protein kinase-3 determines cellular necrotic response to TNF-alpha. *Cell* 2009, **137**(6)**:** 1100-1111.

53. Cho YS, Challa S, Moquin D, Genga R, Ray TD, Guildford M*, et al.* Phosphorylation-driven assembly of the RIP1-RIP3 complex regulates programmed necrosis and virus-induced inflammation. *Cell* 2009, **137**(6)**:** 1112-1123.

54. Zhang DW, Shao J, Lin J, Zhang N, Lu BJ, Lin SC*, et al.* RIP3, an energy metabolism regulator that switches TNF-induced cell death from apoptosis to necrosis. *Science* 2009, **325**(5938)**:** 332-336.

55. Sun L, Wang H, Wang Z, He S, Chen S, Liao D*, et al.* Mixed lineage kinase domain-like protein mediates necrosis signaling downstream of RIP3 kinase. *Cell* 2012, **148**(1-2)**:** 213-227.

56. Wang H, Sun L, Su L, Rizo J, Liu L, Wang LF*, et al.* Mixed lineage kinase domain-like protein MLKL causes necrotic membrane disruption upon phosphorylation by RIP3. *Mol Cell* 2014, **54**(1)**:** 133-146.

57. He S, Wang X. RIP kinases as modulators of inflammation and immunity. *Nat Immunol* 2018, **19**(9)**:** 912-922.

58. Wang L, Du F, Wang X. TNF-alpha induces two distinct caspase-8 activation pathways. *Cell* 2008, **133**(4)**:** 693-703.

59. Micheau O, Tschopp J. Induction of TNF receptor I-mediated apoptosis via two sequential signaling complexes. *Cell* 2003, **114**(2)**:** 181-190.

60. Wilson NS, Dixit V, Ashkenazi A. Death receptor signal transducers: nodes of coordination in immune signaling networks. *Nat Immunol* 2009, **10**(4)**:** 348-355.

61. Zhao J, Jitkaew S, Cai Z, Choksi S, Li Q, Luo J*, et al.* Mixed lineage kinase domain-like is a key receptor interacting protein 3 downstream component of TNF-induced necrosis. *Proc Natl Acad Sci U S A* 2012, **109**(14)**:** 5322-5327.

62. Dondelinger Y, Jouan-Lanhouet S, Divert T, Theatre E, Bertin J, Gough PJ*, et al.* NF-kappaB-Independent Role of IKKalpha/IKKbeta in Preventing RIPK1 Kinase-Dependent Apoptotic and Necroptotic Cell Death during TNF Signaling. *Mol Cell* 2015, **60**(1)**:** 63-76.

63. Vanlangenakker N, Vanden Berghe T, Bogaert P, Laukens B, Zobel K, Deshayes K*, et al.* cIAP1 and TAK1 protect cells from TNF-induced necrosis by preventing RIP1/RIP3-dependent reactive oxygen species production. *Cell Death Differ* 2011, **18**(4)**:** 656-665.

64. Dziedzic SA, Su Z, Jean Barrett V, Najafov A, Mookhtiar AK, Amin P*, et al.* ABIN-1 regulates RIPK1 activation by linking Met1 ubiquitylation with Lys63 deubiquitylation in TNF-RSC. *Nat Cell Biol* 2018, **20**(1)**:** 58-68.

65. Feoktistova M, Geserick P, Kellert B, Dimitrova DP, Langlais C, Hupe M*, et al.* cIAPs block Ripoptosome formation, a RIP1/caspase-8 containing intracellular cell death complex differentially regulated by cFLIP isoforms. *Mol Cell* 2011, **43**(3)**:** 449-463.

66. He S, Liang Y, Shao F, Wang X. Toll-like receptors activate programmed necrosis in macrophages through a receptor-interacting kinase-3-mediated pathway. *Proc Natl Acad Sci U S A* 2011, **108**(50)**:** 20054-20059.

67. Rickard JA, O'Donnell JA, Evans JM, Lalaoui N, Poh AR, Rogers T*, et al.* RIPK1 regulates RIPK3-MLKL-driven systemic inflammation and emergency hematopoiesis. *Cell* 2014, **157**(5)**:** 1175-1188.

68. Dillon CP, Weinlich R, Rodriguez DA, Cripps JG, Quarato G, Gurung P*, et al.* RIPK1 blocks early postnatal lethality mediated by caspase-8 and RIPK3. *Cell* 2014, **157**(5)**:** 1189-1202.

69. Heger K, Wickliffe KE, Ndoja A, Zhang J, Murthy A, Dugger DL*, et al.* OTULIN limits cell death and inflammation by deubiquitinating LUBAC. *Nature* 2018, **559**(7712)**:** 120-124.

70. Lafont E, Draber P, Rieser E, Reichert M, Kupka S, de Miguel D*, et al.* TBK1 and IKKepsilon prevent TNF-induced cell death by RIPK1 phosphorylation. *Nat Cell Biol* 2018, **20**(12)**:** 1389-1399.

71. Hughes MA, Harper N, Butterworth M, Cain K, Cohen GM, MacFarlane M. Reconstitution of the death-inducing signaling complex reveals a substrate switch that determines CD95-mediated death or survival. *Mol Cell* 2009, **35**(3)**:** 265-279.

72. Chang DW, Xing Z, Capacio VL, Peter ME, Yang X. Interdimer processing mechanism of procaspase-8 activation. *EMBO J* 2003, **22**(16)**:** 4132-4142.

73. Brumatti G, Ma C, Lalaoui N, Nguyen NY, Navarro M, Tanzer MC*, et al.* The caspase-8 inhibitor emricasan combines with the SMAC mimetic birinapant to induce necroptosis

and treat acute myeloid leukemia. *Sci Transl Med* 2016, **8**(339)**:** 339ra369.

74. Zhang X, Zhang H, Xu C, Li X, Li M, Wu X*, et al.* Ubiquitination of RIPK1 suppresses programmed cell death by regulating RIPK1 kinase activation during embryogenesis. *Nat Commun* 2019, **10**(1)**:** 4158.

75. Zhang X, Fan C, Zhang H, Zhao Q, Liu Y, Xu C*, et al.* MLKL and FADD Are Critical for Suppressing Progressive Lymphoproliferative Disease and Activating the NLRP3 Inflammasome. *Cell Rep* 2016, **16**(12)**:** 3247-3259.

ACKNOWLEDGEMENTS

We thank Dr. Xiaodong Wang (National Institute of Biological Sciences, Beijing, China) for providing *Ripk3*$^{-/-}$ mice. The authors thank Zhonghui Weng (Shanghai Institute of Nutrition and Health, Chinese Academy of Sciences) for animal studies and Lin Qiu (Shanghai Institute of Nutrition and Health, Chinese Academy of Sciences) for flow cytometry technical support.

AUTHOR CONTRIBUTIONS

X.M.L and H.B.Z designed the study and performed data analyses; X.M.L and L.F carried out most of experiments with assistance from X.X.Z, M.L, L.X.W, J.L.L, X.H.W, Y.J.O, M.Y.X, Y.Z, J.S.D and X.Z.W. H.W.Z and Q.Z assisted with cell death analyses *in vitro* and Fas-induced apoptosis *in vivo*, X.X.W helped to perform mouse breeding. J.B.L, Y.L and Y.W.Z provided essential reagents and intellectual input. H.B.Z and X.M.L coordinated the project and wrote the paper with the help from L.F. H.B.Z supervised the project.

FUNDING

This work was supported by grants from the Strategic Priority Research Program of Chinese Academy of Sciences (Grant No. XDA26040306), the National Natural Science Foundation of China (31970688,31771537,82001684, 81871101) and the National Key Research and Development Program of China (2018YFC1200201, 2016YFC1304900). We also thank support from Shanghai Frontiers Science Center of Cellular Homeostasis and Human Diseases.

ETHIC STATEMENT

Our studies did not include human participates, human data or human tissues. Animal experiments were conducted in accordance with the guidelines of the Institutional Animal Care and Use Committee of the Institute of Nutrition and Health, Shanghai Institutes for Biological Sciences, University of Chinese Academy of Sciences.

COMPETING INTERESTS

The authors declare no competing interests.

Additional information

Supplementary information

Supplementary information includes seven figures.

Correspondence and requests for materials should be addressed to H.B.Z.

Figure Legends

Figure 1. *Casp8*$^{\Delta E385/\Delta E385}$ mice are viable and develop a slight lymphopenia.

(A) Western blot of primary wild-type (WT) MDFs which were treated with TNF-α (40 ng/ml) +Cycloheximide (CHX) (40 μg/ml) (TC) for the indicated time.

(B) Western blot of primary WT MDFs which were treated with TNF-α (20 ng/ml) +Smac mimetic (Smac) (1 μM) +zVAD (20 μM) (TSZ).

(C) Western blot of RIPK1, RIPK3, MLKL, FADD, caspase-8, and GAPDH in the indicated organs of WT (1) and *Casp8*$^{\Delta E385/\Delta E385}$ (2) mice.

(D) Lymph nodes and spleens removed from 16-week old mice of indicated genotypes (scale bar, 1cm).

(E) Dot plot of weight of lymph nodes (parts showed in **Figure 1D**) and spleens of 12- to 16-week old WT, *Casp8*$^{\Delta E385/\Delta E385}$ mice. Bars, mean±SD. *P* values above the asterisk (unpaired, two-tailed t-test) ****$p<0.0001$, compared to the WT mice.

(F) Different cell subsets from spleen, lymph nodes (parts showed in **Figure 1D**) and bone marrow of 12- to 16-week old WT and *Casp8*$^{\Delta E385/\Delta E385}$ mice were analyzed by flow cytometry using the following markers: B cells ($B220^+$ or $CD19^+$), T cells ($CD3^+$), $CD4^+$ T cells ($CD3^+CD4^+CD8^-$), $CD8^+$ T cells ($CD3^+CD8^+CD4^-$), Granulocytes and Macrophages ($CD11b^+$), mature B cells in spleen ($B220^+IgM^+$ or $B220^+CD19^+$), immature and mature B cells in bone marrow ($B220^+$ IgM^+ or $B220^{hi}$ $CD19^{hi}$), progenitor B cells (pro-B) and precursor B cells (pre-B) in bone marrow ($B220^+$ IgM^- or $B220^{low}$ $CD19^{low}$). Bars, mean±SD. *P* values above the asterisk (unpaired, two-tailed t-test) *$p<0.05$, **$p<0.05$, ****$p<0.0001$.

Figure 2. The CASP8(ΔE385) compromises apoptosis, particularly switches TNF-α induced apoptosis to necroptosis.

(A) Western blotting analysis of the indicated protein in primary WT and Casp8ΔE385/ΔE385 thymocytes which were treated with FasL (Jo-2) (1 μg/ml) for the indicated time. The data are representative of three independent experiments.

(B) Primary WT and *Casp8*$^{\Delta E385/\Delta E385}$ MDFs were treated with GSK'872 in different concentration for the indicated time respectively. Bars, mean±SD. *P* values above the asterisk (unpaired, two-tailed t-test) *$p<0.05$, **$p<0.01$, ****$p<0.0001$.

(C) Western blotting analysis of protein expression of caspase-8, cleaved caspase-8, cleaved caspase-3 and GAPDH in primary WT and *Casp8*$^{\Delta E385/\Delta E385}$ MDFs which were treated with GSK'872 (20 μM) for the indicated time.

(D) Mouse survival curve of 8- to 12-week old mice after challenged by anti-Fas antibody (Jo-2, 0.5 μg/g, i.v.). F, female. *P* values alongside the asterisk, two-sided Log-rank (Mantel-Cox) test, **$p<0.01$.

(E) The alanine transaminase (ALT) and aspartate transaminase (AST) levels in serum of the 16-week old WT, *Casp8*$^{\Delta E385/\Delta E385}$ mice 2.5h after anti-Fas injection. Bars, mean±SD. *P* values above the asterisk (unpaired, two-tailed t-test), **$p<0.01$.

(F) Primary WT and *Casp8*$^{\Delta E385/\Delta E385}$ MDFs were treated with TNF-α (20 ng/ml), TNF-α+Smac (1 μM) (TS), TNF-α+CHX (20 μg/ml) (TC) for 5 hours. Bars, mean±SD. P values above the asterisk (unpaired, two-tailed t-test) ***$p<0.001$.

(G) Immunoblotting of the indicated protein expression in primary WT and *Casp8*$^{\Delta E385/\Delta E385}$ MDFs which were treated with TNF-α (40 ng/ml) +CHX (40 μg/ml) (TC) for the indicated time.

Figure 3. The CASP8(ΔE385) promotes necroptosis *in vitro.*

(A) Primary WT and *Casp8*$^{\Delta E385/\Delta E385}$ MDFs were treated with TNF-α (20 ng/ml)+Smac (1 μM)+zVAD (20 μM) (TSZ) and TNF-α+Smac+zVAD+Nec-1 (30 μM) (TSZN) for 6.45 hours, TNF-α+CHX (20 μg/ml)+zVAD (20 μM) (TCZ) and TNF-α+CHX+zVAD+Nec-1 (30 μM) (TCZN) for 4.45 hours. Bars, mean±SD. *P* values above the asterisk (unpaired, two-tailed t-test) ****$p<0.0001$.

(B) Primary WT and *Casp8*$^{\Delta E385/\Delta E385}$ bone marrow derived macrophages (BMDMs) were treated with LPS (100 ng/ml), LPS+zVAD (20 μM) (LZ), LPS+zVAD+Nec-1 (30 μM) (LZN), poly(I:C) (100 μg/ml), poly(I:C)+zVAD (20 μM) (PZ), poly(I:C)+zVAD+Nec-1 (30 μM) (PZN), TNF-α+Smac+zVAD (TSZ), TNF-α+Smac+zVAD+Nec-1 (TSZN) for 3 hours. Bars, mean±SD. *P* values above the asterisk (unpaired, two-tailed t-test) ***$p<0.001$, ****$p<0.0001$.

(C) Immunoblotting of the indicated protein expression in primary WT and *Casp8*$^{\Delta E385/\Delta E385}$ MDFs which were treated with TNF-α (20 ng/ml) +Smac (1 μM) +zVAD (20 μM) (TSZ) for the indicated time.

(D) Immunoblotting of primary WT and *Casp8*$^{\Delta E385/\Delta E385}$ MDFs which were treated with TNF-α (20 ng/ml) +Smac (1 μM) +zVAD (20 μM) (TSZ) for the indicated time.

(E) WT and *Casp8*$^{\Delta E385/\Delta E385}$ MDFs were treated with TNF-α (40 ng/ml)+CHX (40μg/ml)+zVAD (50 uM) for the indicated time, complex II was immunoprecipitated using anti-RIPK1, the recruitment of RIPK3, FADD and caspase-8 were detected by western blotting.

(F) Primary WT and *Casp8*$^{\Delta E385/\Delta E385}$ BMDMs were treated with LPS (200 ng/ml)+zVAD (40 μM) followed by western blot and immunoprecipitation.

(G) Primary WT and *Casp8*$^{\Delta E385/\Delta E385}$ MDFs were treated with TNF-α (40 ng/ml)+CHX (40μg/ml) followed by western blot and immunoprecipitation.

Figure 4. The CASP8(ΔE385) promotes necroptosis *in vivo.*

(A) Mouse survival curve of 8- to 16-week old mice after injection by TNF-α (7 μg each mouse, i.v.). M, male, F, female. *P* values alongside the asterisk, by two-sided Log-rank (Mantel-Cox) test. ****p<0.0001.

(B) Body temperature of 8- to 16-week old mice after injection by TNF-α (7 μg each mouse, i.v.). M, male, F, female. Bars, mean±SD. The significance of body temperature between WT and *Casp8*$^{\Delta E385/\Delta E385}$ mice in the indicated time was described by *P* values below the asterisk (unpaired, two-tailed t-test) *p<0.05, **p<0.01, ****p<0.0001.

(C) Representative peritoneal macrophages flow cytometric dot plots along CD11b versus F4/80 parameters. Untreated (UT), LPS+zVAD (LZ).

(D) Dot plots of CD11b+F4/80+ peritoneal macrophages of 8- to 12-week old WT, *Casp8*$^{\Delta E385/\Delta E385}$ and *Ripk1*$^{+/-}$*Ripk3*$^{-/-}$*Casp8*$^{\Delta E385/\Delta E385}$ mice. Bars, mean+SD. *P* values (unpaired, two-tailed t-test) ****p<0.0001.

Figure 5. *Casp8*$^{\Delta E385/\Delta E385}$*Ripk3*$^{-/-}$ mice develop serious lymphopenia and myeloid bias but prevent the postnatal lethality of *Ripk1*$^{-/-}$ mice.

(A) Spleens images (15 week) (left) and total spleen weight (14- to 17-week old) (right) of the indicated genotype mice. Scale bar, 1 cm. Bars, mean±SD. *P* values above the asterisk (unpaired, two-tailed t-test) ****$p<0.0001$.

(B) The immunocytes cell number in spleen and bone marrow (per tibia and femur) of 14- to 17-week old mice. Bars, mean±SD. *P* values (unpaired, two-tailed t-test) *$p<0.05$, **$p<0.01$, ***$p<0.001$, ****$p<0.0001$.

(C) The B cell and T cell subsets cellularity in spleen and bone marrow (per tibia and femur) of 14- to 17-week old mice. Bars, mean±SD. *P* values (unpaired, two-tailed t-test) *$p<0.05$, **$p<0.01$, ***$p<0.001$, ****$p<0.0001$.

(D) The absolute cell number of the white blood cells and their subsets in the peripheral blood of 14- to 17-week old mice. Bars, mean±SD. *P* values (unpaired, two-tailed t-test) *$p<0.05$, **$p<0.01$, ***$p<0.001$, ****$p<0.0001$.

(E) The B cell and T cell subsets cellularity in the peripheral blood of 14- to 17-week old mice. Bars, mean±SD. *P* values (unpaired, two-tailed t-test) **$p<0.01$, ***$p<0.001$, ****$p<0.0001$.

(F) Mouse survival curve of the given genotypes after birth. *P* values alongside the asterisk, two-sided Log-rank (Mantel-Cox) test, ****$p<0.0001$.

Figure 6. Halving the RIPK1 dosage rescues transplantable lymphopenia and myeloid bias in *Ripk3*$^{-/-}$ *Casp8*$^{\Delta E385/\Delta E385}$ mice.

(A) Spleen images (12 week) (left) and total spleen weight (14-17 week) (right) showed normal sized spleen in the *Ripk1*$^{+/-}$ *Ripk3*$^{-/-}$ *Casp8*$^{\Delta E385/\Delta E385}$ mice. Scale bar, 1 cm. Bars, mean$\pm$SD. *P* values above the asterisk (unpaired, two-tailed t-test) ****$p<0.0001$; ns, no significance.

(B) The absolute cell number of indicated immunocytes in spleen and bone marrow (per tibia and femur) of 14- to 17-week old age matched mice. Bars, mean$\pm$SD. *P* values (unpaired, two-tailed t-test) **$p<0.01$, ***$p<0.001$, ****$p<0.0001$; ns, no significance.

(C) The cell number of white blood cells and their subsets in the peripheral blood of 14- to 17-week old mice. Bars, mean$\pm$SD. *P* values (unpaired, two-tailed t-test) *$p<0.05$, ***$p<0.001$, ****$p<0.0001$; ns, no significance.

(D) The absolute cell number and percentage of white blood cells and their subsets in the peripheral blood of 6-month old recipients. Bars, mean$\pm$SD. *P* values (unpaired, two-tailed t-test) *$p<0.05$, **$p<0.01$, ***$p<0.001$, ****$p<0.0001$.

(E) The absolute cell number of the immunocytes and their subsets in the spleen of 6-month old recipients. Bars, mean$\pm$SD. *P* values (unpaired, two-tailed t-test) *$p<0.05$, **$p<0.01$, ***$p<0.001$, ****$p<0.0001$.

(F) The absolute cellularity of the immunocytes and their subsets in the bone marrow per tibia and femur of 6-month old recipients. Bars, mean$\pm$SD. *P* values (unpaired, two-tailed t-test) *$p<0.05$, **$p<0.01$, ***$p<0.001$.

Supplemental Figure Legends

Figure S1. *Casp8$^{ΔE385/ΔE385}$* mice developed normally.

(A) Schematic diagram of wild-type *Casp8* locus and *Casp8$^{ΔE385/ΔE385}$* allele. Three adjacent nucleotides (red AAG and red asterisk labeled in locus) were removed resulted in the deletion of Glutamic acid (E) in the 385 position of caspase-8 protein sequence. The mutation was confirmed by sequencing.

(B) Photograph of an 8-week-old *Casp8$^{ΔE385/ΔE385}$* mouse alongside a WT littermate.

(C) Primary WT and *Casp8$^{ΔE385/ΔE385}$* BMDMs were treated with LPS (200ng/ml)+BV6 (4 μM) followed by western blot.

(D) Western blot of RIPK1, RIPK3, MLKL, FADD, caspase-8, and GAPDH in the indicated organs of WT (1) and *Casp8$^{ΔE385/ΔE385}$* (2) mice.

(E) Representative images of Hematoxylin and eosin-stained liver, lung and skin sections of 12-week old WT, *Casp8$^{ΔE385/ΔE385}$* mice (scale bar, 100 μm).

Figure S2. The CASP8(ΔE385) caspase-8 compromises Fas-induced apoptosis *in vitro* and *in vivo.*

(A) The primary WT and *Casp8$^{ΔE385/ΔE385}$* thymocytes died after prolonged incubation with FasL (Jo-2). FACS analysis of the primary WT (upper panels) and *Casp8$^{ΔE385/ΔE385}$* (lower panels) thymocytes incubated for 24 h with FasL (2μg/ml) and stained with FITC-annexin V and PI.

(B) Representative images (n>3) of Hematoxylin and eosin-stained (H&E) liver sections and cleaved caspase-3 (CC3) immunohistochemistry of the 16-week old WT, *Casp8$^{ΔE385/ΔE385}$* mice treated with anti-Fas i.v. for 2.5h (scale bar, 100 μm).

(C) Western blot of livers of 16-week old WT and *Casp8$^{ΔE385/ΔE385}$* mice which were treated with anti-Fas antibody (Jo-2, 0.5 μg/g, i.v.) for 2.5h. Each number represents a mouse.

Figure S3. The CASP8(ΔE385) switches TNF-*α* induced apoptosis to necroptosis and promotes necroptosis.

(A) Primary WT and *Casp8*$^{\Delta E385/\Delta E385}$ MDFs were treated with TNF-α (40 ng/ml) +Smac (2 μM) for the indicated time.

(B) Immunoblotting of primary WT and *Casp8*$^{\Delta E385/\Delta E385}$ MDFs which were treated with TNF-α (40 ng/ml) +CHX (40 μg/ml) +zVAD (20 μM) (TCZ) for the indicated time.

(C) Immunoblotting of the indicated protein expression in primary WT and *Casp8*$^{\Delta E385/\Delta E385}$ BMDMs which were challenged by LPS (200 ng/ml) (L), LPS+zVAD (40 μM) (LZ) for 6 hours, respectively.

(D) Primary WT and *Casp8*$^{\Delta E385/\Delta E385}$ BMDMs were treated with LPS (200ng/ml)+BV6 (4 μM) followed by western blot and immunoprecipitation.

Figure S4. The *Ripk3*$^{-/-}$*Casp8*$^{\Delta E385/\Delta E385}$ and *Mlkl*$^{-/-}$*Casp8*$^{\Delta E385/\Delta E385}$ mice developed lymphopenia and myeloid bias.

(A) Representative flow cytometric images (n>3) of B cells and T cells in spleen of 16-week old mice.

(B) The percentage of B cells (CD19^{+}), T cells (CD3^{+}) and myeloid-derived cells (CD11b^{+}) in spleen and bone marrow (per tibia and femur) of 14- to 17-week old mice. Bars, mean$\pm$SD. *P* values (unpaired, two-tailed t-test) *$p<0.05$, **$p<0.01$, ***$p<0.001$, ****$p<0.0001$.

(C) The percentage of immunocyte subsets in spleen and bone marrow (per tibia and femur) of 14- to 17-week old mice. Bars, mean$\pm$SD. *P* values (unpaired, two-tailed t-test) *$p<0.05$, **$p<0.01$, ***$p<0.001$, ****$p<0.0001$.

(D) Representative flow cytometric images (n>3) of immature and mature B cells (IgM^{+}B220^{+}/ B220^{hi}CD19^{hi}), progenitor B cells (pro-B) and precursor B cells (pre-B) (B220^{+}IgM^{-}/B220^{low}CD19^{low}) in bone marrow of 16-week old mice.

Figure S5. The *Ripk3*$^{-/-}$*Casp8*$^{\Delta E385/\Delta E385}$ partially rescued perinatal lethality of

***Ripk1$^{-/-}$* mice but developed RIPK1 dosage-dependent lymphopenia.**

(A) Photograph of *Ripk1$^{-/-}$Ripk3$^{-/-}$Casp8$^{ΔE385/ΔE385}$* mouse (P13, post-natal day 13) alongside a control mouse (P13). Straight arrows: several small lesion, loss of hair.

(B) The absolute cell number of B cell and T cell subsets in spleen and bone marrow (per tibia and femur) of 14- to 17-week old mice. Bars, mean±SD. *P* values (unpaired, two-tailed t-test) *p<0.05, **p<0.01, ****p<0.0001.

(C) Representative flow cytometric images (n>3) of B cells and T cells in spleen of 12-week old mice.

(D) Representative flow cytometric images (n>3) of immature and mature B cells (IgM^{+}B220^{+}/ B220^{hi}CD19^{hi}), progenitor B cells (pro-B) and precursor B cells (pre-B) (B220^{+}IgM^{-}/B220^{low}CD19^{low}) in bone marrow of 12-week old mice.

Figure S6. The lethally irradiated mice receiving *Ripk3$^{-/-}$Casp8$^{ΔE385/ΔE385}$* and *Mlkl$^{-/-}$Casp8$^{ΔE385/ΔE385}$* bone marrows developed leucopenia.

(A) Experimental design diagram.

(B) Spleens images (left) and total spleen weight (right) of 6-month old recipients. Scale bar, 1 cm. Bars, mean±SD. *P* values (unpaired, two-tailed t-test) **p<0.01.

(C) The red blood cells number, platelets number and hemoglobin concentration in the peripheral blood of 6-month old recipients. Bars, mean±SD. *P* values (unpaired, two-tailed t-test) *p<0.05.

(D) The percentage of the B cell and T cell subsets in the peripheral blood of 6-month old recipients. Bars, mean±SD. *P* values (unpaired, two-tailed t-test) **p<0.01, ***p<0.001, ****p<0.0001.

Figure S7. Caspase-8 auto-cleavage inhibits necroptosis downstream of TNFR1 by cleaving RIPK1 and negatively regulating complex II formation and associates with RIPK3/MLKL to protect from lymphopenia.

In TNF-α induced apoptosis occurred in *Casp8$^{ΔE385/ΔE385}$* cells, caspase-8 cannot auto-

cleavage between the large and small catalytic subunits which impairs efficient caspase-8 activation. CASP8(ΔE385) attenuates its function of cleaving RIPK1 and caspase-3 which results in impaired apoptosis and increased RIPK1 activation. This abnormal RIPK1 activation brings the stronger RIPK1 phosphorylation and in turn enhanced RIPK1-RIPK3-MLKL cascade, which finally switches caspase-3 dependent apoptosis to necroptosis. In TNF-α induced necroptosis with addition of zVAD, CASP8(ΔE385), unable to auto-cleavage, functions as a scaffold recruiting more FADD, RIPK3, and RIPK1 into complex II and stabilizing complex II, which results in dramatically activated RIPK1-RIPK3-MLKL cascade phosphorylation and in turn excessive necroptosis. Moreover, CASP8(ΔE385) associating with RIPK1 promotes lymphopenia which is inhibited by RIPK3 and MLKL.

Figure 1

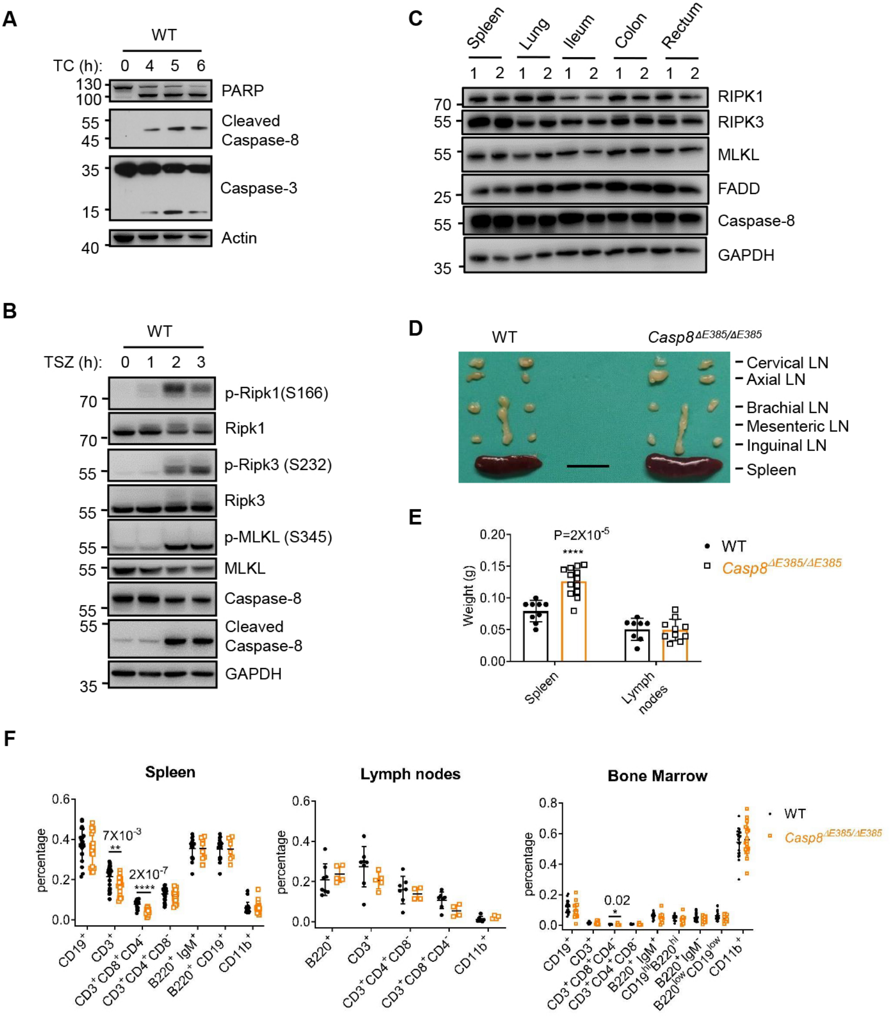
A
WT
TC (h): 0 4 5 6
130
100
PARP
55
45
Cleaved Caspase-8
35
15
Caspase-3
40
Actin
B
WT
TSZ (h): 0 1 2 3
70
p-Ripk1(S166)
70
Ripk1
55
p-Ripk3 (S232)
55
Ripk3
55
p-MLKL (S345)
55
MLKL
55
Caspase-8
55
Cleaved Caspase-8
35
GAPDH
C
Spleen
Lung
Ileum
Colon
Rectum
1 2 1 2 1 2 1 2 1 2
70
RIPK1
55
RIPK3
55
MLKL
25
FADD
55
Caspase-8
35
GAPDH
D
WT
Casp8ΔE385/ΔE385
Cervical LN
Axial LN
Brachial LN
Mesenteric LN
Inguinal LN
Spleen
E
P=2X10-5
****
Weight (g)
0.20
0.15
0.10
0.05
0.00
Spleen
Lymph nodes
WT
Casp8ΔE385/ΔE385
F
Spleen
percentage
0.6
0.4
0.2
0.0
7X10-3
**
2X10-7
****
CD19+
CD3+
CD3+CD8+CD4-
CD3+CD4+CD8-
B220+ IgM+
B220+ CD19+
CD11b+
Lymph nodes
percentage
0.6
0.4
0.2
0.0
B220+
CD3+
CD3+CD4+CD8-
CD3+CD8+CD4-
CD11b+
Bone Marrow
percentage
0.8
0.6
0.4
0.2
0.0
0.02
*
CD19+
CD3+
CD3+CD8+CD4-
CD3+CD4+CD8-
B220+ IgM+
CD19hiB220hi
B220+IgM-
B220lowCD19low
CD11b+
WT
Casp8ΔE385/ΔE385

Figure 2

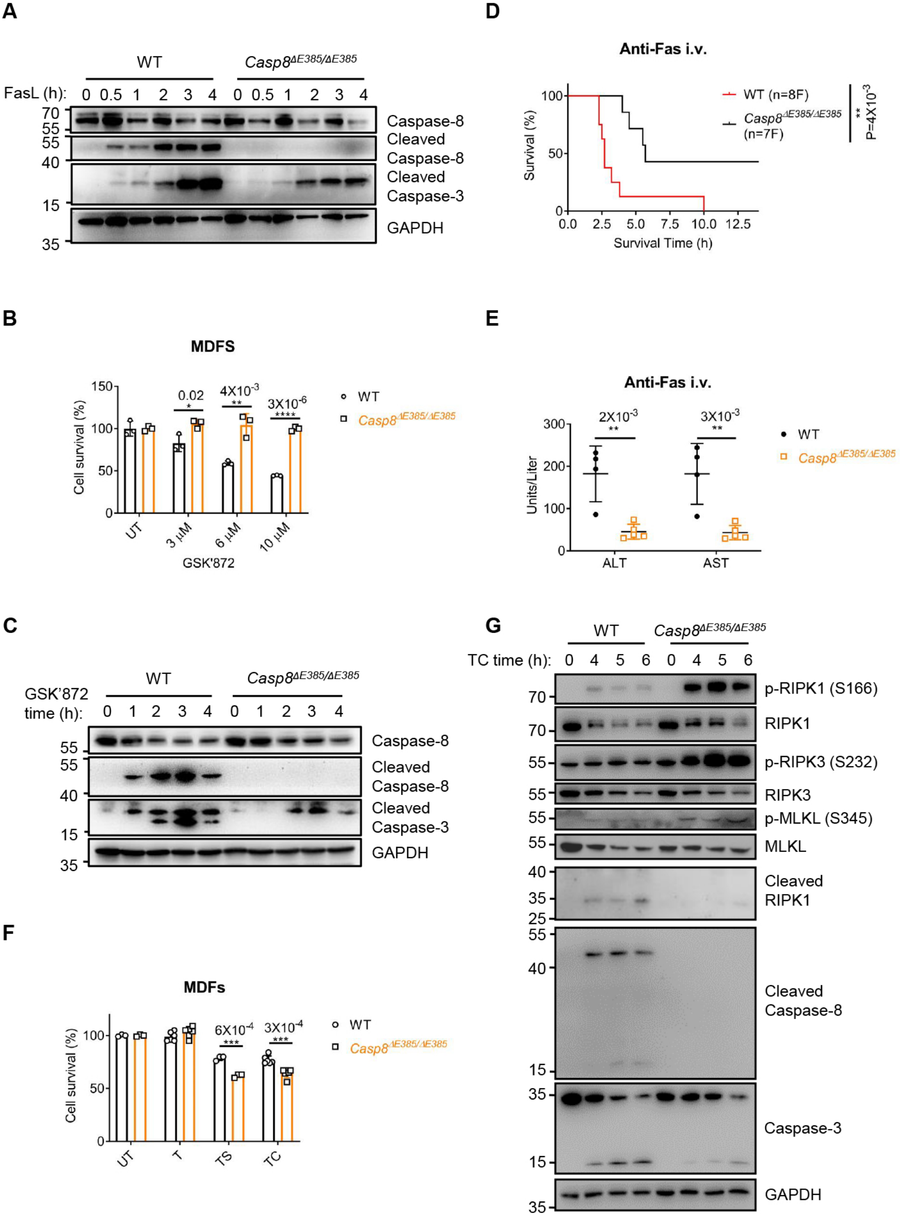

A
WT
Casp8ΔE385/ΔE385
FasL (h): 0 0.5 1 2 3 4 0 0.5 1 2 3 4
70
55
55
40
15
35
Caspase-8
Cleaved Caspase-8
Cleaved Caspase-3
GAPDH
B
MDFS
Cell survival (%)
150
100
50
0
0.02
*
4X10-3
**
3X10-6
****
UT
3 μM
6 μM
10 μM
GSK'872
WT
Casp8ΔE385/ΔE385
C
WT
Casp8ΔE385/ΔE385
GSK'872 time (h): 0 1 2 3 4 0 1 2 3 4
55
55
40
15
35
Caspase-8
Cleaved Caspase-8
Cleaved Caspase-3
GAPDH
D
Anti-Fas i.v.
Survival (%)
100
50
0
0.0 2.5 5.0 7.5 10.0 12.5
Survival Time (h)
WT (n=8F)
Casp8ΔE385/ΔE385 (n=7F)
** P=4X10-3
E
Anti-Fas i.v.
Units/Liter
300
200
100
0
2X10-3
**
3X10-3
**
ALT
AST
WT
Casp8ΔE385/ΔE385
F
MDFs
Cell survival (%)
100
50
0
6X10-4
***
3X10-4
***
UT
T
TS
TC
WT
Casp8ΔE385/ΔE385
G
WT
Casp8ΔE385/ΔE385
TC time (h): 0 4 5 6 0 4 5 6
70
70
55
55
55
55
40
35
25
55
40
15
35
15
35
p-RIPK1 (S166)
RIPK1
p-RIPK3 (S232)
RIPK3
p-MLKL (S345)
MLKL
Cleaved RIPK1
Cleaved Caspase-8
Caspase-3
GAPDH

Figure 3

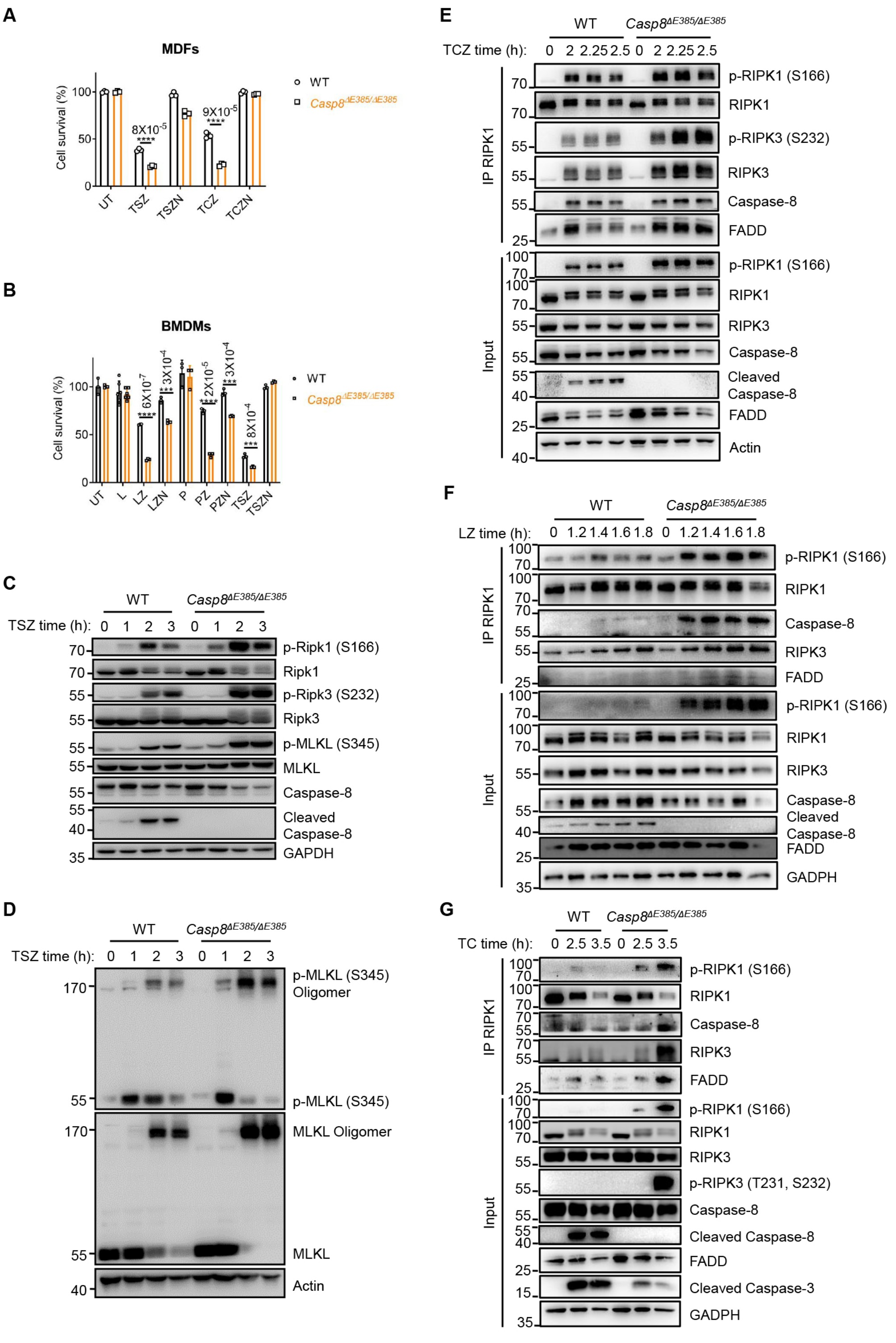
A
MDFs
Cell survival (%)
100
50
0
UT
TSZ
TSZN
TCZ
TCZN
8X10-5
****
9X10-5
****
WT
Casp8ΔE385/ΔE385
B
BMDMs
Cell survival (%)
100
50
0
UT
L
LZ
LZN
P
PZ
PZN
TSZ
TSZN
6X10-7
****
3X10-4
***
2X10-5
****
3X10-4
***
8X10-4
***
WT
Casp8ΔE385/ΔE385
C
WT
Casp8ΔE385/ΔE385
TSZ time (h): 0 1 2 3 0 1 2 3
p-Ripk1 (S166)
Ripk1
p-Ripk3 (S232)
Ripk3
p-MLKL (S345)
MLKL
Caspase-8
Cleaved Caspase-8
GAPDH
D
WT
Casp8ΔE385/ΔE385
TSZ time (h): 0 1 2 3 0 1 2 3
p-MLKL (S345) Oligomer
p-MLKL (S345)
MLKL Oligomer
MLKL
Actin
E
WT
Casp8ΔE385/ΔE385
TCZ time (h): 0 2 2.25 2.5 0 2 2.25 2.5
IP RIPK1
p-RIPK1 (S166)
RIPK1
p-RIPK3 (S232)
RIPK3
Caspase-8
FADD
Input
p-RIPK1 (S166)
RIPK1
RIPK3
Caspase-8
Cleaved Caspase-8
FADD
Actin
F
WT
Casp8ΔE385/ΔE385
LZ time (h): 0 1.2 1.4 1.6 1.8 0 1.2 1.4 1.6 1.8
IP RIPK1
p-RIPK1 (S166)
RIPK1
Caspase-8
RIPK3
FADD
Input
p-RIPK1 (S166)
RIPK1
RIPK3
Caspase-8
Cleaved Caspase-8
FADD
GADPH
G
WT
Casp8ΔE385/ΔE385
TC time (h): 0 2.5 3.5 0 2.5 3.5
IP RIPK1
p-RIPK1 (S166)
RIPK1
Caspase-8
RIPK3
FADD
Input
p-RIPK1 (S166)
RIPK1
RIPK3
p-RIPK3 (T231, S232)
Caspase-8
Cleaved Caspase-8
FADD
Cleaved Caspase-3
GADPH

Figure 4

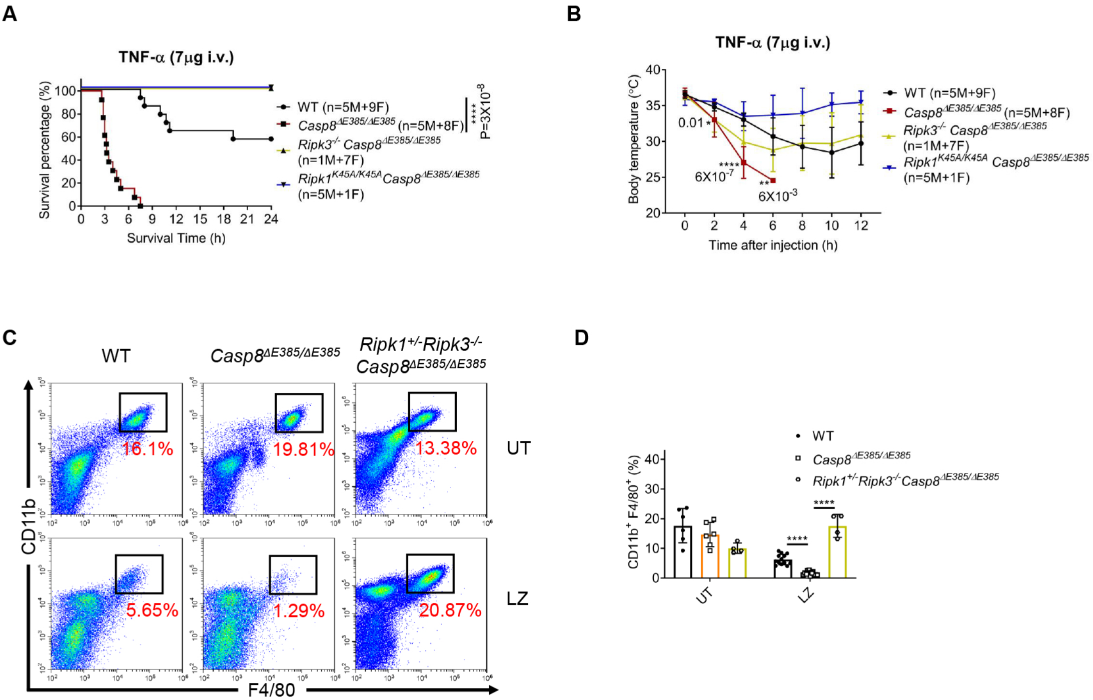
A
TNF-α (7μg i.v.)
Survival percentage (%)
Survival Time (h)
WT (n=5M+9F)
Casp8ΔE385/ΔE385 (n=5M+8F)
Ripk3-/- Casp8ΔE385/ΔE385 (n=1M+7F)
Ripk1K45A/K45A Casp8ΔE385/ΔE385 (n=5M+1F)
****
P=3X10-8
B
TNF-α (7μg i.v.)
Body temperature (°C)
Time after injection (h)
0.01 *
****
6X10-7
**
6X10-3
WT (n=5M+9F)
Casp8ΔE385/ΔE385 (n=5M+8F)
Ripk3-/- Casp8ΔE385/ΔE385 (n=1M+7F)
Ripk1K45A/K45A Casp8ΔE385/ΔE385 (n=5M+1F)
C
WT
Casp8ΔE385/ΔE385
Ripk1+/-Ripk3-/- Casp8ΔE385/ΔE385
CD11b
F4/80
16.1%
19.81%
13.38%
UT
5.65%
1.29%
20.87%
LZ
D
WT
Casp8ΔE385/ΔE385
Ripk1+/-Ripk3-/-Casp8ΔE385/ΔE385
CD11b+ F4/80+ (%)
****
****
UT
LZ

Figure 5

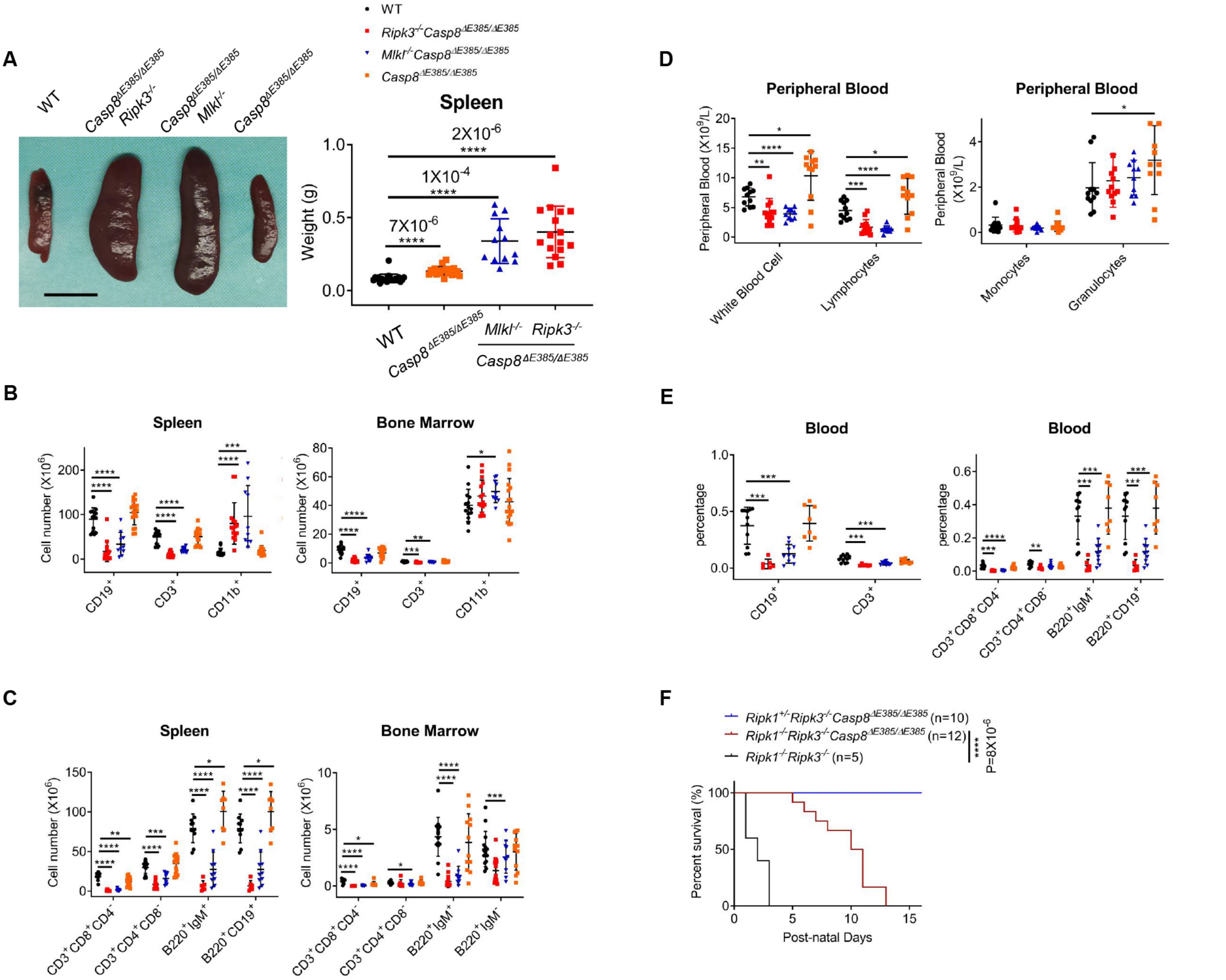

A
WT
Casp8ΔE385/ΔE385 Ripk3-/-
Casp8ΔE385/ΔE385 Mlkl-/-
Casp8ΔE385/ΔE385
Spleen
Weight (g)
2X10-6
1X10-4
7X10-6
Mlkl-/- Ripk3-/-
B
Spleen
Bone Marrow
Cell number (X10^6)
CD19+
CD3+
CD11b+
C
CD3+CD8+CD4-
CD3+CD4+CD8-
B220+IgM+
B220+CD19+
B220+IgM-
D
Peripheral Blood
Peripheral Blood (X10^9/L)
White Blood Cell
Lymphocytes
Monocytes
Granulocytes
E
Blood
percentage
F
Ripk1+/-Ripk3-/-Casp8ΔE385/ΔE385 (n=10)
Ripk1-/-Ripk3-/-Casp8ΔE385/ΔE385 (n=12)
Ripk1-/-Ripk3-/- (n=5)
P=8X10-6
Percent survival (%)
Post-natal Days

Figure 6

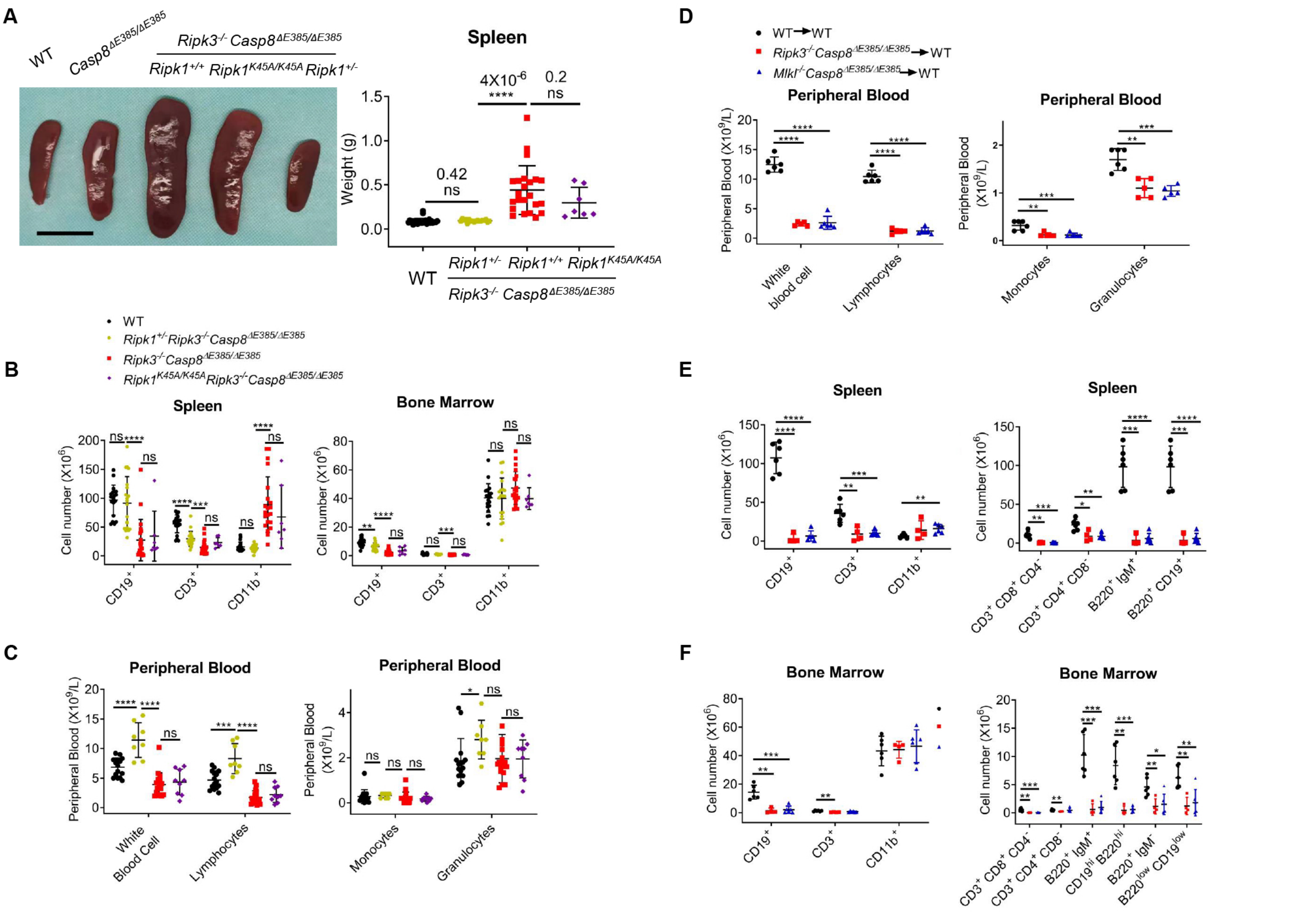

A
WT
Casp8$^{ΔE385/ΔE385}$
Ripk3$^{-/-}$ Casp8$^{ΔE385/ΔE385}$
Ripk1$^{+/+}$ Ripk1$^{K45A/K45A}$ Ripk1$^{+/-}$
Spleen
Weight (g)
0.42 ns
4X10^{-6} ****
0.2 ns
WT
Ripk1$^{+/-}$ Ripk1$^{+/+}$ Ripk1$^{K45A/K45A}$
Ripk3$^{-/-}$ Casp8$^{ΔE385/ΔE385}$
B
WT
Ripk1$^{+/-}$Ripk3$^{-/-}$Casp8$^{ΔE385/ΔE385}$
Ripk3$^{-/-}$Casp8$^{ΔE385/ΔE385}$
Ripk1$^{K45A/K45A}$Ripk3$^{-/-}$Casp8$^{ΔE385/ΔE385}$
Spleen
Bone Marrow
Cell number (X10^6)
CD19$^+$
CD3$^+$
CD11b$^+$
C
Peripheral Blood
Peripheral Blood (X10^9/L)
White Blood Cell
Lymphocytes
Monocytes
Granulocytes
D
WT→WT
Ripk3$^{-/-}$Casp8$^{ΔE385/ΔE385}$→WT
Mlkl$^{-/-}$Casp8$^{ΔE385/ΔE385}$→WT
Peripheral Blood
White blood cell
Lymphocytes
Monocytes
Granulocytes
E
Spleen
CD3$^+$ CD8$^+$ CD4$^-$
CD3$^+$ CD4$^+$ CD8$^-$
B220$^+$ IgM$^+$
B220$^+$ CD19$^+$
F
Bone Marrow
CD19^{hi} B220^{hi}
B220$^+$ IgM$^-$
B220^{low} CD19^{low}

Figure S1

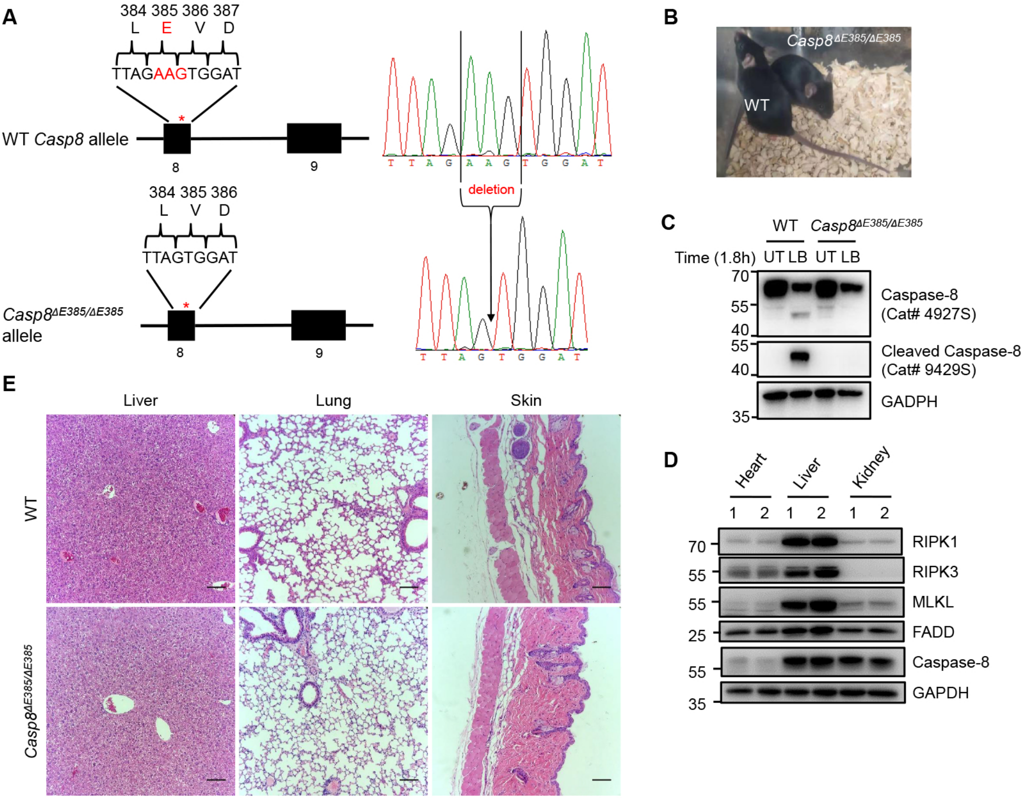
A
384 385 386 387
L E V D
TTAGAAGTGGAT
WT Casp8 allele
8
9
T T A G A A G T G G A T
deletion
384 385 386
L V D
TTAGTGGAT
Casp8ΔE385/ΔE385 allele
8
9
T T A G T G G A T
B
Casp8ΔE385/ΔE385
WT
C
WT Casp8ΔE385/ΔE385
Time (1.8h) UT LB UT LB
70
55
40
Caspase-8 (Cat# 4927S)
55
40
Cleaved Caspase-8 (Cat# 9429S)
GADPH
35
D
Heart Liver Kidney
1 2 1 2 1 2
70 RIPK1
55 RIPK3
55 MLKL
25 FADD
55 Caspase-8
GAPDH
35
E
Liver Lung Skin
WT
Casp8ΔE385/ΔE385

Figure S2

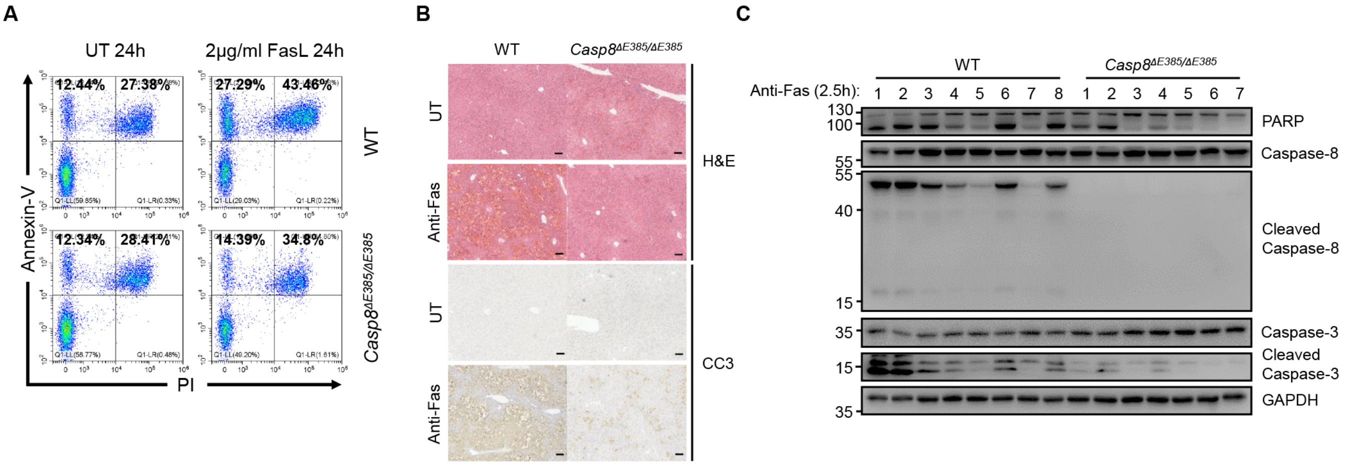

A
UT 24h
2μg/ml FasL 24h
12.44%
27.38%
27.29%
43.46%
12.34%
28.41%
14.39%
34.8%
Q1-LL(59.85%)
Q1-LR(0.33%)
Q1-LL(29.03%)
Q1-LR(0.22%)
Q1-LL(58.77%)
Q1-LR(0.48%)
Q1-LL(49.20%)
Q1-LR(1.61%)
WT
$Casp8^{\Delta E385/\Delta E385}$
Annexin-V
PI
B
WT
$Casp8^{\Delta E385/\Delta E385}$
UT
Anti-Fas
UT
Anti-Fas
H&E
CC3
C
WT
$Casp8^{\Delta E385/\Delta E385}$
Anti-Fas (2.5h): 1 2 3 4 5 6 7 8 1 2 3 4 5 6 7
130
100
55
55
40
15
35
15
35
PARP
Caspase-8
Cleaved Caspase-8
Caspase-3
Cleaved Caspase-3
GAPDH

Figure S3

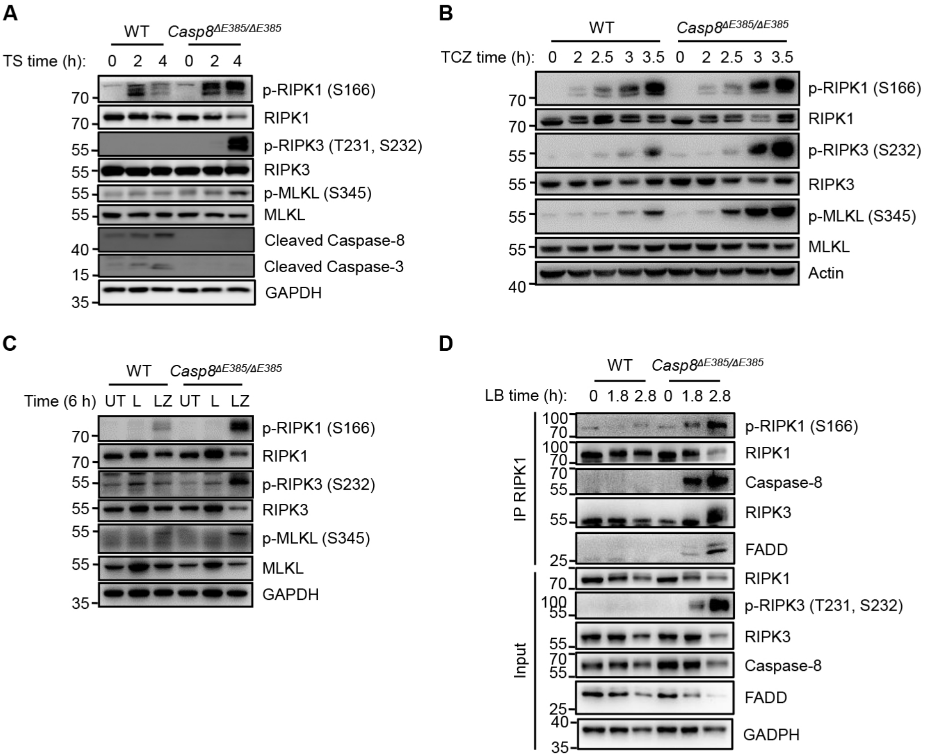
A
WT
Casp8ΔE385/ΔE385
TS time (h): 0 2 4 0 2 4
p-RIPK1 (S166)
RIPK1
p-RIPK3 (T231, S232)
RIPK3
p-MLKL (S345)
MLKL
Cleaved Caspase-8
Cleaved Caspase-3
GAPDH
B
TCZ time (h): 0 2 2.5 3 3.5 0 2 2.5 3 3.5
p-RIPK3 (S232)
Actin
C
Time (6 h) UT L LZ UT L LZ
D
LB time (h): 0 1.8 2.8 0 1.8 2.8
IP RIPK1
Input
Caspase-8
FADD
GADPH

Figure S4

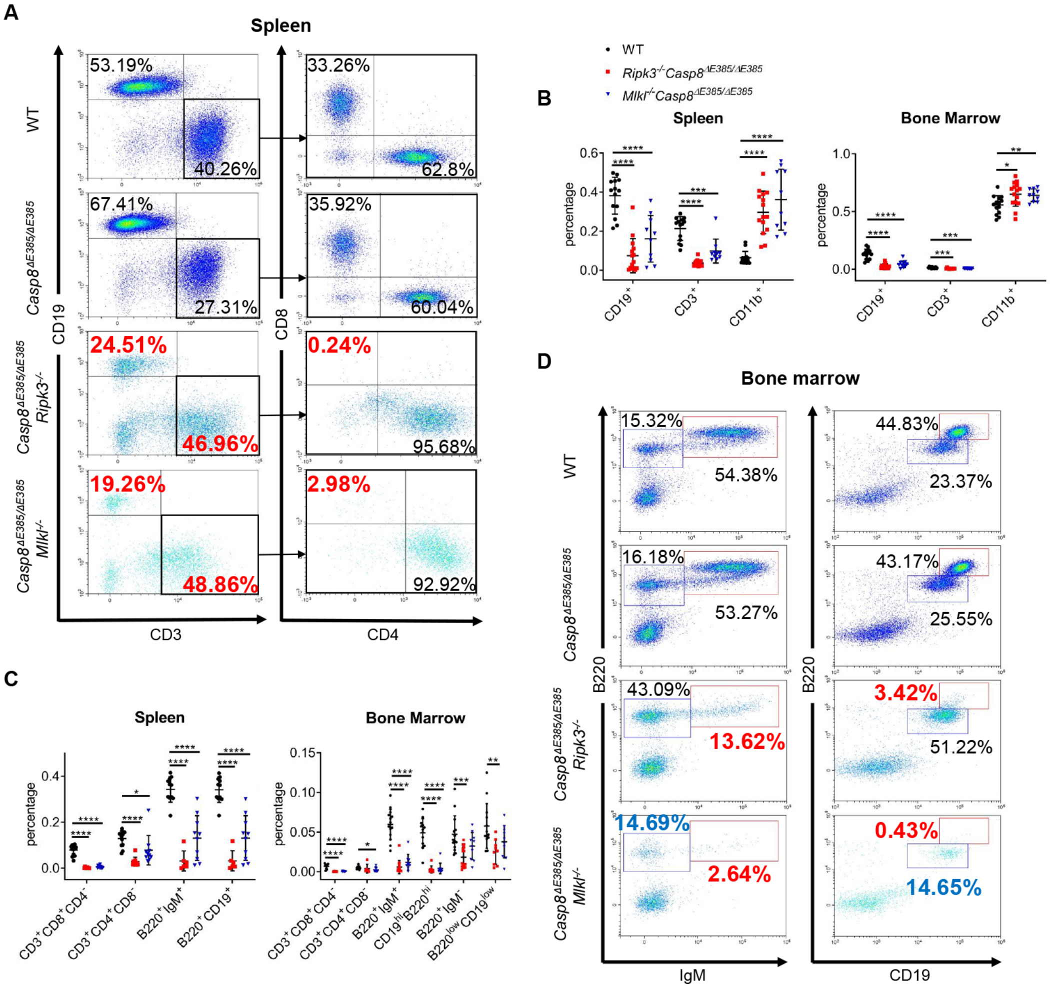
A
Spleen
WT
53.19%
40.26%
33.26%
62.8%
Casp8ΔE385/ΔE385
67.41%
27.31%
35.92%
60.04%
Casp8ΔE385/ΔE385 Ripk3-/-
24.51%
46.96%
0.24%
95.68%
Casp8ΔE385/ΔE385 Mlkl-/-
19.26%
48.86%
2.98%
92.92%
CD19
CD3
CD8
CD4
B
WT
Ripk3-/-Casp8ΔE385/ΔE385
Mlkl-/-Casp8ΔE385/ΔE385
Spleen
Bone Marrow
percentage
CD19+
CD3+
CD11b+
C
Spleen
Bone Marrow
percentage
CD3+CD8+CD4-
CD3+CD4+CD8-
B220+IgM+
B220+CD19+
CD19hiB220hi
B220+IgM-
B220lowCD19low
D
Bone marrow
WT
15.32%
54.38%
44.83%
23.37%
Casp8ΔE385/ΔE385
16.18%
53.27%
43.17%
25.55%
Casp8ΔE385/ΔE385 Ripk3-/-
43.09%
13.62%
3.42%
51.22%
Casp8ΔE385/ΔE385 Mlkl-/-
14.69%
2.64%
0.43%
14.65%
B220
IgM
CD19

Figure S5

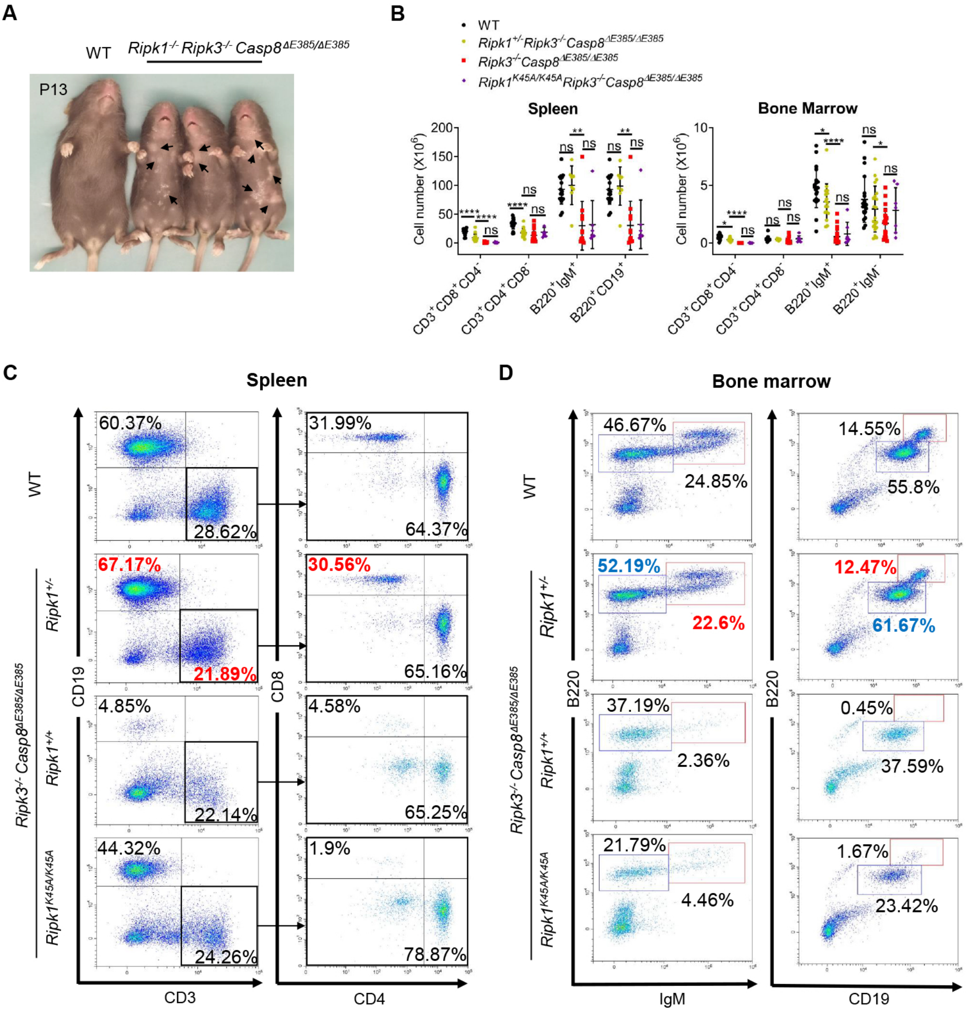

A
WT
Ripk1-/- Ripk3-/- Casp8ΔE385/ΔE385
P13
B
WT
Ripk1+/-Ripk3-/-Casp8ΔE385/ΔE385
Ripk3-/-Casp8ΔE385/ΔE385
Ripk1K45A/K45ARipk3-/-Casp8ΔE385/ΔE385
Spleen
Bone Marrow
Cell number (X10^6)
CD3+CD8+CD4-
CD3+CD4+CD8-
B220+IgM+
B220+CD19+
B220+IgM-
C
Spleen
D
Bone marrow
WT
Ripk1+/-
Ripk1+/+
Ripk1K45A/K45A
Ripk3-/- Casp8ΔE385/ΔE385
CD19
CD3
CD8
CD4
B220
IgM
60.37%
28.62%
31.99%
64.37%
67.17%
21.89%
30.56%
65.16%
4.85%
22.14%
4.58%
65.25%
44.32%
24.26%
1.9%
78.87%
46.67%
24.85%
14.55%
55.8%
52.19%
22.6%
12.47%
61.67%
37.19%
2.36%
0.45%
37.59%
21.79%
4.46%
1.67%
23.42%

Figure S6

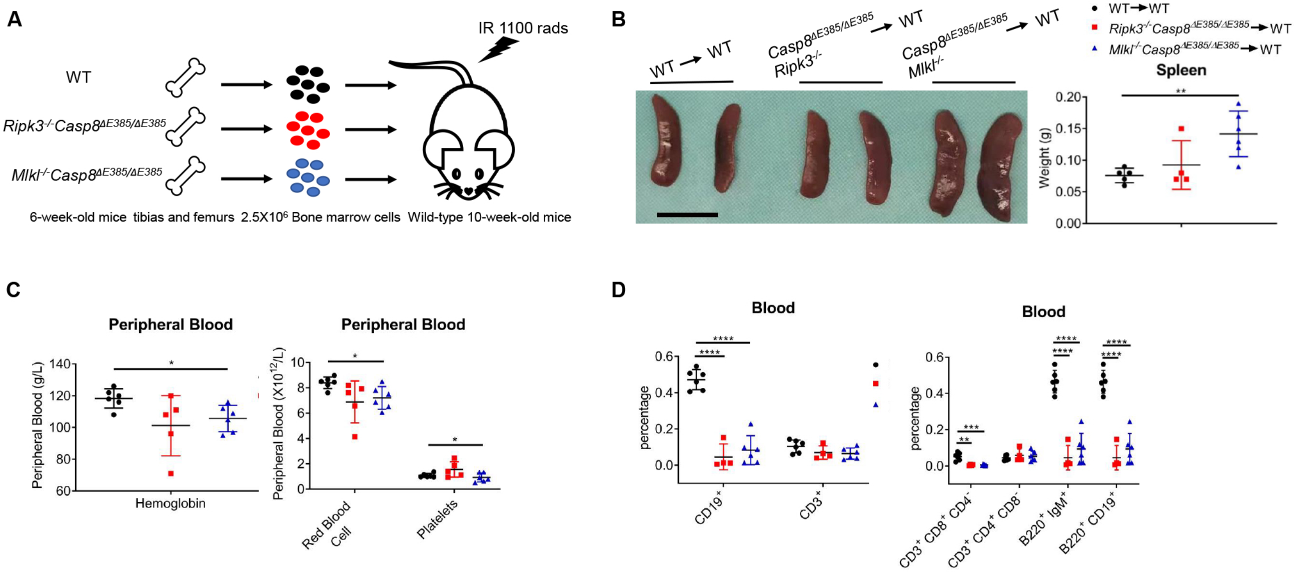
A
IR 1100 rads
WT
Ripk3-/-Casp8ΔE385/ΔE385
Mlkl-/-Casp8ΔE385/ΔE385
6-week-old mice tibias and femurs 2.5X10^6 Bone marrow cells Wild-type 10-week-old mice
B
WT → WT
Casp8ΔE385/ΔE385 Ripk3-/- → WT
Casp8ΔE385/ΔE385 Mlkl-/- → WT
WT→WT
Ripk3-/-Casp8ΔE385/ΔE385→WT
Mlkl-/-Casp8ΔE385/ΔE385→WT
Spleen
Weight (g)
0.20
0.15
0.10
0.05
0.00
**
C
Peripheral Blood
Peripheral Blood (g/L)
140
120
100
80
60
Hemoglobin
*
Peripheral Blood
Peripheral Blood (X10^12/L)
10
8
6
4
2
0
Red Blood Cell
Platelets
*
*
D
Blood
percentage
0.6
0.4
0.2
0.0
CD19+
CD3+
****
****
Blood
percentage
0.6
0.4
0.2
0.0
CD3+ CD8+ CD4-
CD3+ CD4+ CD8-
B220+ IgM+
B220+ CD19+
***
**
****
****
****
****

Figure S7

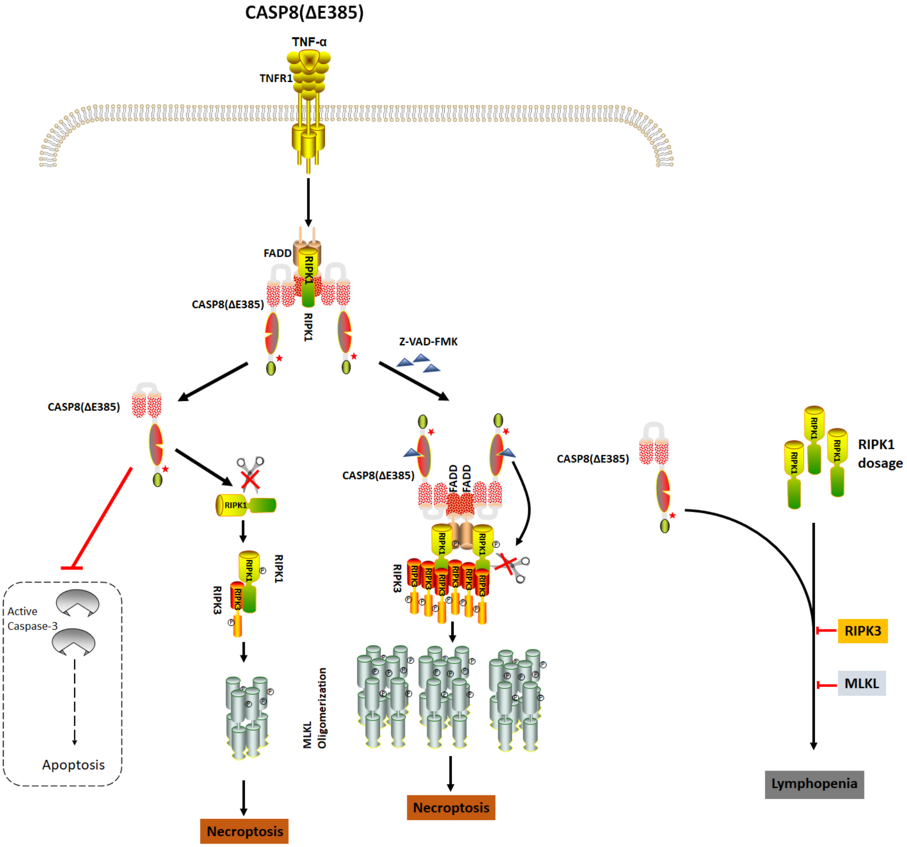

CASP8(ΔE385)
TNF-α
TNFR1
FADD
RIPK1
CASP8(ΔE385)
RIPK1
Z-VAD-FMK
CASP8(ΔE385)
RIPK1
RIPK1
RIPK3
MLKL Oligomerization
Active Caspase-3
Apoptosis
Necroptosis
CASP8(ΔE385)
FADD
FADD
RIPK1
RIPK3
Necroptosis
CASP8(ΔE385)
RIPK1 dosage
RIPK3
MLKL
Lymphopenia
