# Supplementary material for: Caspase-8 auto-cleavage regulates programmed cell death and collaborates with RIPK3/MLKL to prevent lymphopenia
Source: Cell Death Differ. 2022 Jan 21;29(8):1500–12. doi: 10.1038/s41418-022-00938-9 (PMC9345959; doi:10.1038/s41418-022-00938-9)
Supplement: Supplementary file 11 — Supplemental Figure Legends [file 41418_2022_938_MOESM11_ESM.docx]

**Supplemental Figure Legends**

**Figure S1. *Casp8^ΔE385/ΔE385^* mice developed normally.**

**(A)** Schematic diagram of wild-type *Casp8* locus and *Casp8^ΔE385/ΔE385^* allele. Three adjacent nucleotides (red AAG and red asterisk labeled in locus) were removed resulted in the deletion of Glutamic acid (E) in the 385 position of caspase-8 protein sequence. The mutation was confirmed by sequencing.

**(B)** Photograph of an 8-week-old *Casp8^ΔE385/ΔE385^* mouse alongside a WT littermate.

**(C)** Primary WT and *Casp8^ΔE385/ΔE385^* BMDMs were treated with LPS (200ng/ml)+BV6 (4 μM) followed by western blot.

**(D)** Western blot of RIPK1, RIPK3, MLKL, FADD, caspase-8, and GAPDH in the indicated organs of WT (1) and *Casp8^ΔE385/ΔE385^* (2) mice.

**(E)** Representative images of Hematoxylin and eosin-stained liver, lung and skin sections of 12-week old WT, *Casp8^ΔE385/ΔE385^* mice (scale bar, 100 μm).

**Figure S2. The CASP8(ΔE385) caspase-8 compromises Fas-induced apoptosis *in vitro* and *in vivo.***

**(A)** The primary WT and *Casp8^ΔE385/ΔE385^* thymocytes died after prolonged incubation with FasL (Jo-2). FACS analysis of the primary WT (upper panels) and *Casp8^ΔE385/ΔE385^* (lower panels) thymocytes incubated for 24 h with FasL (2μg/ml) and stained with FITC-annexin V and PI.

**(B)** Representative images (n>3) of Hematoxylin and eosin-stained (H&E) liver sections and cleaved caspase-3 (CC3) immunohistochemistry of the 16-week old WT, *Casp8^ΔE385/ΔE385^* mice treated with anti-Fas i.v. for 2.5h (scale bar, 100 μm).

**(C)** Western blot of livers of 16-week old WT and *Casp8^ΔE385/ΔE385^* mice which were treated with anti-Fas antibody (Jo-2, 0.5 μg/g, i.v.) for 2.5h. Each number represents a mouse.

**Figure S3. The CASP8(ΔE385) switches TNF-α induced apoptosis to necroptosis and promotes necroptosis.**

**(A)** Primary WT and *Casp8^ΔE385/ΔE385^* MDFs were treated with TNF-α (40 ng/ml) +Smac (2 μM) for the indicated time.

**(B)** Immunoblotting of primary WT and *Casp8^ΔE385/ΔE385^* MDFs which were treated with TNF-α (40 ng/ml) +CHX (40 μg/ml) +zVAD (20 μM) (TCZ) for the indicated time.

**(C)** Immunoblotting of the indicated protein expression in primary WT and *Casp8^ΔE385/ΔE385^* BMDMs which were challenged by LPS (200 ng/ml) (L), LPS+zVAD (40 μM) (LZ) for 6 hours, respectively.

**(D)** Primary WT and *Casp8^ΔE385/ΔE385^* BMDMs were treated with LPS (200ng/ml)+BV6 (4 μM) followed by western blot and immunoprecipitation.

**Figure S4. The *Ripk3^-/-^Casp8^ΔE385/ΔE385^* and *Mlkl^-/-^Casp8^ΔE385/ΔE385^* mice developed lymphopenia and myeloid bias.**

**(A)** Representative ﬂow cytometric images (n>3) of B cells and T cells in spleen of 16-week old mice.

**(B)** The percentage of B cells (CD19^+^), T cells (CD3^+^) and myeloid-derived cells (CD11b^+^) in spleen and bone marrow (per tibia and femur) of 14- to 17-week old mice. Bars, mean+SD. *P* values (unpaired, two-tailed t-test) **p*<0.05, ***p*<0.01, ****p*<0.001, *****p*<0.0001.

**(C)** The percentage of immunocyte subsets in spleen and bone marrow (per tibia and femur) of 14- to 17-week old mice. Bars, mean+SD. *P* values (unpaired, two-tailed t-test) **p*<0.05, ***p*<0.01, ****p*<0.001, *****p*<0.0001.

**(D)** Representative ﬂow cytometric images (n>3) of immature and mature B cells (IgM^+^B220^+^/ B220^hi^CD19^hi^), progenitor B cells (pro-B) and precursor B cells (pre-B) (B220^+^IgM^-^/B220^low^CD19^low^) in bone marrow of 16-week old mice.

**Figure S5. The *Ripk3^-/-^Casp8^ΔE385/ΔE385^* partially rescued perinatal lethality of *Ripk1^-/-^* mice but developed RIPK1 dosage-dependent lymphopenia.**

**(A)** Photograph of *Ripk1^-/-^Ripk3^-/-^Casp8^ΔE385/ΔE385^* mouse (P13, post-natal day 13) alongside a control mouse (P13). Straight arrows: several small lesion, loss of hair.

**(B)** The absolute cell number of B cell and T cell subsets in spleen and bone marrow (per tibia and femur) of 14- to 17-week old mice. Bars, mean+SD. *P* values (unpaired, two-tailed t-test) **p*<0.05, ***p*<0.01, *****p*<0.0001.

**(C)** Representative ﬂow cytometric images (n>3) of B cells and T cells in spleen of 12-week old mice.

**(D)** Representative ﬂow cytometric images (n>3) of immature and mature B cells (IgM^+^B220^+^/ B220^hi^CD19^hi^), progenitor B cells (pro-B) and precursor B cells (pre-B) (B220^+^IgM^-^/B220^low^CD19^low^) in bone marrow of 12-week old mice.

**Figure S6. The lethally irradiated mice receiving *Ripk3^-/-^Casp8^ΔE385/ΔE385^* and *Mlkl^-/-^Casp8^ΔE385/ΔE385^* bone marrows developed leucopenia.**

**(A)** Experimental design diagram.

**(B)** Spleens images (left) and total spleen weight (right) of 6-month old recipients. Scale bar, 1 cm. Bars, mean+SD. *P* values (unpaired, two-tailed t-test) ***p*<0.01.

**(C)** The red blood cells number, platelets number and hemoglobin concentration in the peripheral blood of 6-month old recipients. Bars, mean+SD. *P* values (unpaired, two-tailed t-test) **p*<0.05.

**(D)** The percentage of the B cell and T cell subsets in the peripheral blood of 6-month old recipients. Bars, mean+SD. *P* values (unpaired, two-tailed t-test) ***p*<0.01, ****p*<0.001, *****p*<0.0001.

**Figure S7. Caspase-8 auto-cleavage inhibits necroptosis downstream of TNFR1 by cleaving RIPK1 and negatively regulating complex II formation and associates with RIPK3/MLKL to protect from lymphopenia.**

In TNF-α induced apoptosis occurred in *Casp8^ΔE385/ΔE385^* cells, caspase-8 cannot auto-cleavage between the large and small catalytic subunits which impairs efficient caspase-8 activation. CASP8(ΔE385) attenuates its function of cleaving RIPK1 and caspase-3 which results in impaired apoptosis and increased RIPK1 activation. This abnormal RIPK1 activation brings the stronger RIPK1 phosphorylation and in turn enhanced RIPK1-RIPK3-MLKL cascade, which finally switches caspase-3 dependent apoptosis to necroptosis. In TNF-α induced necroptosis with addition of zVAD, CASP8(ΔE385), unable to auto-cleavage, functions as a scaffold recruiting more FADD, RIPK3, and RIPK1 into complex II and stabilizing complex II, which results in dramatically activated RIPK1-RIPK3-MLKL cascade phosphorylation and in turn excessive necroptosis. Moreover, CASP8(ΔE385) associating with RIPK1 promotes lymphopenia which is inhibited by RIPK3 and MLKL.
